# Gene Silencing and Over-Expression Studies in Concurrence With Promoter Specific Elicitations Reveal the Central Role of *WsCYP85A69* in Biosynthesis of Triterpenoids in *Withania somnifera* (L.) Dunal

**DOI:** 10.3389/fpls.2019.00842

**Published:** 2019-07-05

**Authors:** Arti Sharma, Gulzar A. Rather, Prashant Misra, Manoj K. Dhar, Surrinder K. Lattoo

**Affiliations:** ^1^Plant Biotechnology Division, CSIR-Indian Institute of Integrative Medicine, Jammu, India; ^2^School of Biotechnology, Faculty of Life Sciences, University of Jammu, Jammu, India

**Keywords:** *Withania somnifera*, brassinosteroids, stigmasterol, withanolides, cytochrome P450 monooxygenase

## Abstract

*Withania somnifera* (Ashwagandha) synthesizes a wide spectrum of triterpenoids that are produced via an intricate isoprenoid pathway whose biosynthetic and regulatory mechanism remains elusive. Their pharmacological examination position them as potent bioactive molecules, hence demanding their copious production. Previous investigations have revealed that P450 monooxygenases are pivotal enzymes involved in the biosynthetic machinery of various metabolites and assist in decorating their core skeletal structures. The present study entails the isolation and functional characterization of castasterone synthase (*CYP85A69*) from *W. somnifera*. The full length *WsCYP85A69*, having an open reading frame of 1413 bp, encodes 470 amino acid residues. Further, *in vitro* conversion of 6-deoxocastasterone into castasterone validated its oxidative functionality. Product formation was confirmed using LC-PDA-MS with a *m/z* value of 506 [M+ACN]^+^. *In planta* transient over-expression of *WsCYP85A69* significantly enhanced castasterone, stigmasterol and withanolides (WS-I, WS-II, WS-III). Artificial micro-RNA mediated silencing of *WsCYP85A69* resulted in the reduced accumulation of castasterone, stigmasterol and withanolides (WS-I, WS-II, WS-III). Altogether, these non-complementary approaches plausibly suggest a key role of *WsCYP85A69* in the biosynthesis of castasterone and the accumulation of withanolides and stigmasterol. Furthermore, a promoter analysis of *WsCYP85A69* resulted in the identification of several potential *cis*-regulatory elements. Elicitations, given on the basis of identified *cis*-regulatory elements, demonstrated methyl jasmonate as an effective inducer of *WsCYP85A69*. Overall, these empirical findings suggest that functional characterization of *WsCYP85A69* may conceivably be helpful to unravel the mechanism of brassinosteroids biosynthesis and could also pave the way for targeted metabolic engineering.

## Introduction

Triterpenoids are 30-carbon compounds that have fascinating structural frameworks with indispensable pharmacological properties (Sawai and Saito, 2011; Knoch et al., 2018). These are pervasive in the plant kingdom and have become significant targets for metabolic engineering because of their diverse pharmacological properties (Sandjo and Kuete, 2013; Seki et al., 2015). They are widely distributed in various forms like phytosterols, withanolides, phytosteroids including brassinosteroids (Haralampidis et al., 2002; Sandjo and Kuete, 2013).

*Withania somnifera* (L.) Dunal (Solanaceae) is a small shrub which grows copiously in various climatic conditions in India including tropical, sub-tropical and semi-temperate climates (Misra et al., 2010). It is a reputed multipurpose medicinal plant and is unique in synthesizing various types of secondary metabolites including withanolides, alkaloids and steroids, and phytosterols etc., (Bhat et al., 2012; Gupta et al., 2015). Steroids regulate growth and development of *W. somnifera* and also possess certain putative therapeutic applications. They might induce the apoptosis of prostate and breast cancerous cells (Steigerová et al., 2010, 2012), and have antiviral effects against measles, herpesvirus and arenavirus (Wachsman et al., 2000). Further sterols serve as a universal precursor for withanolides production (Singh et al., 2015). Withanolides possess an array of therapeutic properties and as such have attracted significant scientific attention to exploit these compounds for pharmacological purposes. Chemical analysis has revealed that withanolides are produced in minute amounts (0.001–0.5% dry weights) (Singh et al., 2015) and consequently biotechnological interventions are required for their copious production. Owing to the endemic and therapeutic potential of *W. somnifera*, a metabolic engineering program is being executed for a higher and purposeful production of its characteristic phytoconstituents.

Brassinosteroids (BRs) are naturally occurring polyhydroxylated steroids which are involved in growth-promoting activities (Bartwal et al., 2013). They regulate plant growth and developmental processes involving germination, cell elongation, photo-morphogenesis etc., and also play a significant role in combating stress related conditions (Gudesblat and Russinova, 2011; Tang et al., 2016). Reduction in the content of BRs lead to altered leaf morphology, extreme dwarfism, delayed flowering and senescence, abnormal vascular development and reduced male fertility (Clouse, 2011; Wei and Li, 2016). Therefore, regulation and maintenance of BR levels in plants are crucial for various biological functions (Tanaka et al., 2003). From previous reports, it has been suggested that the distribution of BRs vary among different parts of plants (0.01–100 ng/g fw) (Takatsuto, 1994). Furthermore, a plethora of studies have been executed on plant responses mediated by BRs in relation to biotic and abiotic stresses (El-Mashad and Mohamed, 2012). From previous studies, exogenous application of brassinolide enhanced resistance against various pathogens in tobacco and rice, suggesting their role in the innate immunity system of higher plants (Nakashita et al., 2003). BRs also regulate various flavonoid and phenol biosynthetic pathway enzymes such as phenylalanine ammonia-lyase in *Vitis vinifera* (Ahammed et al., 2013; Xi et al., 2013). Likewise, the alteration in total withanolides production occurs due to various stress conditions. For instance, heat stress had increased 2.6 and 4.9% of withanolides’ content at 48 and 58°C, respectively, compared to the control (Sharma and Puri, 2017). Likewise, under various phytohormone treatments including MeJA, salicylic acid, and gibberellic acid, a higher production of withanolides have been observed (Bhat et al., 2012; Rana et al., 2014). These experimental findings suggest that exogenous stress may probably produce brassinosteroids that might be involved in the synthesis of isoprenoids, phenols, or alkaloids for environmental adaptation and defense. Although BRs have been identified from various members of the solanaceae family, the genes involved in their biosynthesis are yet to be deciphered. So, in the present study, we have focused on the biosynthetic pathway of brassinosteroids in *Withania somnifera*.

**FIGURE 1:**
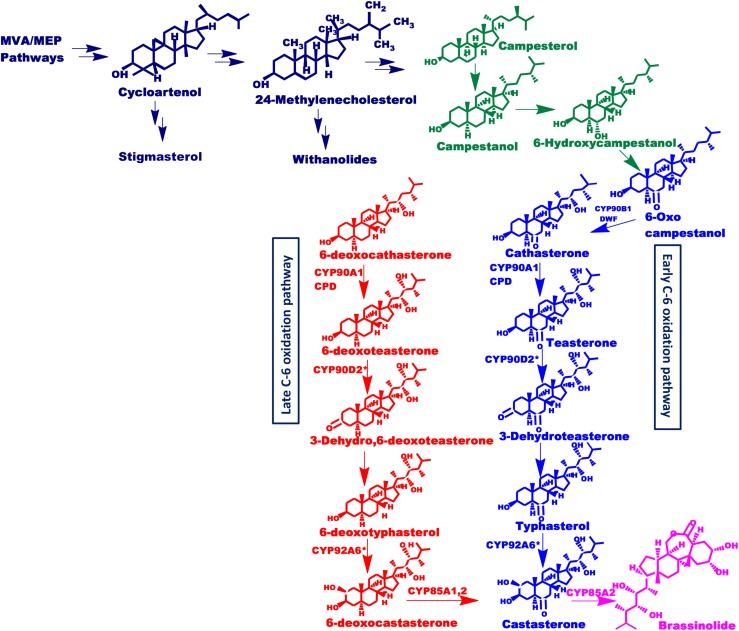
Putative brassinosteroids biosynthetic pathway. Schematic representation of brassinosteroids biosynthetic pathway showing early and late C-6 oxidation pathways. Mevalonate and non-mevalonate pathways synthesize cycloartenol which leads to the synthesis of isofucosterol and 24-methylenecholesterol which further synthesize stigmasterol and withanolides as well as brassinolide respectively, driven via separate routes. This 24-methylenecholesterol then undergoes various reactions to form active brassinosteroids. Enzymes involved are: Dwarf4 (DWF4): DET2, CYP85A1, CYP85A2, CYP90A1, and CYP90B1 (found in Arabidopsis) and CYP90D2 (Hong et al., 2003) and CYP92A6 designated with an asterisk present in rice and pea, respectively.

Brassinolide (BL) and castasterone are the most active form of brassinosteroids. However, more than 70 different analogs of brassinosteroid have been identified and characterized to date (Tarkowská et al., 2016). The biosynthetic pathway of BL from campesterol has been elucidated in suspension cell cultures of *Catharanthus roseus*, using isotope-labeling of intermediates and their identification via gas chromatography-mass spectrometry (GC-MS) (Sakurai, 1999). The analysis of BR biosynthetic enzymes and perception components is done using BR-deficient and -insensitive dwarf mutants from Arabidopsis (*Arabidopsis thaliana*), pea (*Pisum sativum*), tomato (*Lycopersicon esculentum*) and rice (*Oryza sativa*) (Fujioka and Yokota, 2003; Li and Peng, 2003). Such studies, using these mutants, have revealed that cytochrome P450 monooxygenases (P450s) oxidize C-2, C-3, C-6, C-22, C-23, and C-26 of brassinosteroids shown in Figure 1. *P450s* are heme-thiolate monooxygenase enzymes that constitute a large family of proteins present in the plant kingdom (Nelson and Werck-Reichhart, 2011). They catalyze important biochemical reactions in the metabolism of vitamins, steroids, fatty acids, and other chemicals including oxidation, hydroxylation, isomerization, and dehydration (Werck-Reichhart and Feyereisen, 2000). Various steps in brassinosteroids biosynthesis are catalyzed by members of the *CYP85A* family (Wang et al., 2017). The *CYP85* clan is a larger clan that makes up ∼13% of total plant P450s and includes *CYP85, CYP87, CYP88*, *CYP90*, *CYP702*, *CYP707*, *CYP708, CYP716, CYP718, CYP720, CYP722, CYP724*, and *CYP728* families (Bak et al., 2011). This clan involves different genes that have similar yet distinctive functions. *CYP85A* encodes BR-C6-oxidases which catalyzes the synthesis of brassinolide and castasterone (CS), the two most active BRs (Castle et al., 2005; Nomura and Bishop, 2006; Cheon et al., 2013). *CYP85A1* is known to catalyze the conversion of 6-DeoxoCS to CS in *Arabidopsis* whereas, *CYP85A2* and *CYP85A3* convert 6-DeoxoCS into brassinolide via castasterone (Bishop et al., 1996; Nomura et al., 2005). Besides their role in the BR activation, enzymes of the *CYP85A* family also catalyze the C-6 oxidation of 6-deoxotyphasterol, 3-dehydro-6-deoxoteasterone, and 6-deoxoteasterone (Shimada et al., 2001). Therefore, *CYP85A* is an important target for genetic interventions and modulation toward enhanced production of brassinosteroids.

Against this backdrop, we have successfully isolated, cloned and characterized *CYP85A69*, from *Withania somnifera*. Its heterologous expression in *S. cerevisiae* WAT 11 strain and *in vitro* enzymatic assay confirmed its oxidative functionality via efficiently converting 6-deoxocastasterone into castasterone in presence of NADPH. It was confirmed via LC-PDA-MS analysis. Further, its transient over-expression assay in the homologous host revealed an upsurge in the transcript levels of the *CYP85A69* gene with a concomitant increase in castasterone, stigmasterol and withanolides levels. In addition, amiRNA mediated silencing resulted in the reduction of *WsCYP85A69* mRNA transcript levels vis-à-vis castasterone, stigmasterol and withanolides (WS-I, WS-II and WS-III) content. Both these non-complimentary approaches facilitated the functional characterization of *CYP85A69* from *W. somnifera*. Moreover, the promoter was isolated and potential *cis*-acting regulatory elements were identified for elicitation studies. Further, MeJA was found to be strong inducer of *CYP85A69* expressions as compared to ABA and CT.

## Materials and Methods

### Chemicals

6-deoxocastasterone, castasterone were purchased from BOC SCI INc., β-nicotinamide adenine dinucleotide 2′-phosphate reduced tetrasodium salt hydrate (NADPH) methanol, chloroform, and ethanol were procured from Sigma-Aldrich (St. Louis, MO, United States). All solvents used were of HPLC grade and were bought from Renkem, Inc., (Phillipsburg, NJ, United States).

### Plant Material, RNA Isolation and cDNA Synthesis

Withaferin A (WS-3) rich genetic stock of *W. somnifera* designated as WS-Y-08, raised via inter-varietal hybridization (Lattoo et al., 2009) was grown at the IIIM experimental farm (Council of scientific and industrial research, Indian Institute of Integrative Medicine, Jammu, India, 32° 44′ N longitude, 74° 55′ E latitude; 305 m in altitude). Plants grown under controlled conditions of a growth chamber (Percival Scientific IntellusUltraConnect C9, United States) were used for experimental procedures including elicitation, aMIR and transient assay studies. Treated samples were collected and processed for RNA isolation or HPLC analysis. RNA isolation was performed using the SV Total RNA Isolation System (Promega, Madison, WI, United States) as per the manufacturer’s protocol and instantly kept at −80°C till further proceedings. Quantification of RNAs was performed using a Nanodrop spectrophotometer (AstraAuriga, Cambridge, United Kingdom) by analyzing the absorbance at 260 nm as well as ratio of absorbance at 260 nm and 280 nm (A260/280). However, assessment of the quality was also determined by loading RNA on 1% formaldehyde denatured agarose gel electrophoresis. Further RNA was used for first strand cDNA synthesis by using the Revert Aid First Strand cDNA Synthesis Kit (Thermo-scientific, Vilnius, Lithuania). The reaction included a total volume of 20 μl with 4 μg total RNA, 10 mM oligo (dT) primers, the reaction was incubated at 65°C for 5 min afterward 10 mM dNTPs, 1 μl M-MuLV reverse transcriptase (200 U/ml) and 4 μl of 5× first strand buffer (250 mM KCl, 20 mM MgCl_2_, 50 mM DTT, 250 mM Tris–HCl, pH 8.3) was added in the reaction which was incubated at 42°C for 60 min followed by inactivation of reverse transcriptase at 70°C for 5 min.

### Amplification of *WsCYP85A69*

Cytochrome P450 monooxygenase sequences from different plant species were retrieved from the GenBank database at the National Centre for Biotechnology Information (NCBI) and further aligned using clustalW to determine their conserved regions. This conserved region was then used to design the degenerate primers (Supplementary Table S1 and Supplementary File S5). Amplification of the degenerate fragment of *WsCYP85A69* from the cDNA was performed using an optimized polymerase chain reaction under the following cycling conditions: 95°C for 5 min (one cycle), 95°C for 35 s (35 cycles), 50°C for 40 s (35 cycles) and 72°C for 50 s (35 cycles) followed by a final extension of 72°C for 10 min in a thermal cycler (Bio-Rad Laboratories, Hercules, CA, United States). The resulted amplicons were analyzed on 1.2% agarose gel electrophoresis followed by ligation into the pTZ57R/T vector (Fermentas, Burlington, Canada), and transformed into the *Escherichia coli* DH5α host strain. The construct so generated was sequenced using a big terminator cycle sequencing kit (Applied Biosystems, Foster City, CA, United States) with an ABI PRISM® 3130×L genetic analyzer (Applied Biosystems, Foster City, CA, United States). Further, the BLASTn program was used for a similarity search in the obtained nucleotide sequence of the putative *CYP85* degenerate construct and subsequently used for designing RACE primers.

### 5′ and 3′ RACE PCR

An RLM-RACE kit was used to isolate the remaining 5′ as well as 3′ cDNA ends of the putative *WsCYP85A69* gene according to the product manual (Ambion, Austin, TX, United States). For this, synthesis of 5′ and 3′ RACE-ready first-strand cDNA was performed using the protocol provided by the manufacturer. The resultant cDNAs were amplified using RACE primers, as listed in Table 1, in two rounds of PCR to amplify 5′ and 3′ ends, respectively. The first round of PCR included a 5′ RACE-OUT primer complementary to the 5′ RACE adapter sequence attached with cDNA and 5′ *CYP85A69*-OUT primers, followed by nested PCR using 5′ RACE-IN as an inner adapter specific primer and a 5′ CYP85*A69*-IN primer. For both rounds of PCR, reaction mixtures included 1.0 μl cDNA as a template (except for nested PCR in which amplified products of outer PCR were used as a template), 2 μl of each primer (5′ *CYP85A69*-OUT, 5′ RACE-OUT for first round and *5*′*CYP85A69*-IN, 5′ RACE-IN in the nested reaction), 45 μl master Mix (34.5 μl PCR-grade water, 2.5 mM MgCl_2_, 200 μM dNTPs, 2.5 U Taq DNA polymerase and amplified under the following thermocycling conditions: 95°C for 3 min (one cycle), 95°C for 35 s (35 cycles), 58°C for 30 s (35 cycles), 72°C for 2 min (35 cycles) with a final extension at 72°C of 10 min. The amplified products obtained after nested PCR of both 5′ and 3′ RACE PCR were analyzed in 1.2% agarose gel, purified by gel extraction and ligated into a pTZ57R/T vector (Fermentas, Burlington, Canada). These mixtures were transformed into DH5α cells (New England Biolabs, Herts, United Kingdom). The positive clones were confirmed via colony PCR further subjected to plasmid isolation using a DNA plasmid Miniprep Kit (Promega, Madison, WI, United States) and further subjected to sequencing using M13 primers.

**TABLE 1:** *Cis*-regulatory elements present in the promoter region of *CYP85* gene as predicted via PlantCare and PLACE server tool.

***Cis*-Elements**	**Position**	**Signal Sequence**	**Putative Function**
TATA-box	167 (+), 169 (+), 164 (+), 168 (+), 420 (−), 459 (−)	TATA, TAATA	Core promoter element around -30 of transcription start
CAAT-box	96 (+), 120 (+), 140 (+), 184 (+), 284 (+), 346 (+), 200 (−), 553 (−)	CAAT, CAATT, CAAAT, CCAAT	Common *cis*-acting element in promoter and enhancer regions
MYB2 CONSENSUSAT	178 (+) 276 (+) 573 (+)	YAACKG	Binding site for all animal MYB and at least two plant MYB proteins ATMYB1 and ATMYB2
ABRE motif	102 (−), 256 (+), 257 (+), 521 (−)	ACGTG, CACGTG	*Cis*-acting element involved in the abscisic acid responsiveness
MYC CONSENSUSAT	120 (−)	CANNTG	MYC recognition site found in the promoters of the dehydration-responsive gene rd22
TATC-box	296 (−)	TATCCCA	*Cis*-acting element involved in gibberellin-responsiveness
WRKY Box	146 (−), 327 (−), 226 (+)	TGAC, TTGAC	Recognized by WRKY proteins
CGTCA motif	145 (+)	CGTC(G)A	*Cis*-Acting regulatory element involved in the MeJa responsiveness
P-Box, TATC-Box	424 (+), 270 (−)	CCTTTTG, TATCCCA	Gibberellin-responsive element
G-Box	102 (−), 256 (+), 521 (−)	CACGTA	*Cis*-acting regulatory element involved in light responsiveness
TC-rich repeats	554 (+)	ATTTTCTTCA	*Cis*-acting element involved in defense and stress responsiveness
ANAERO3 CONSENSUS	483 (+)	TCATCAC	Anaerobic genes involved in the fermentative pathway

### Full-Length Cloning of *WsCYP85A69*

After isolating the core fragment, 5′ and 3′ RACE sequences, these were further compared and aligned to generate the full-length ORF of *WsCYP85A69.* Consequently, full length gene-specific primers [GSPF, GSPR (Supplementary Table S1)] were generated to amplify the full-length coding sequence of the gene. For its amplification, high fidelity proof-reading DNA polymerase (New England Biolabs, Herts, United Kingdom) was used with the following PCR conditions; 95°C for 5 min (one cycle), 95°C for 35 s (35 cycles), 55°C for 40 s (35 cycles), 72°C for 90 s (35 cycles) and a final extension of 10 min at 72°C. The amplified PCR product was loaded on 1.5% agarose gels and UV light was used for its visualization. Consequently, the resultant amplified product was purified using a gel extraction method, further ligated in a blunt ended pJET vector. The ligation mixture obtained was used to transform the *E. coli* DH5α strain to produce the positive *WsCYP85A69*-pJET containing colonies. These colonies were confirmed via colony PCR and subsequently used for plasmid isolation.

### *In silico* Analysis

The obtained nucleotide sequence was subjected to BLAST^1^ for a similarity search. Further, for the prediction of the ORF of *WsCYP85A69*, the Expasy translation tool^2^ was used. The translation tool was used to predict the amino acid sequence of *WsCYP85A69*, and further homologous sequences of taxonomically diverse plant species were aligned using the ClustalW program^3^ with default parameters (Larkin et al., 2007). In addition to these, ORFs were searched for retrieving homologous sequences from different plant species using BLASTp^4^, followed by aligning them using the log-expectation (MUSCLE) alignment tool^5^ with the default parameters. Phylogenetic analysis was performed to determine the degree of evolutionary relatedness using the maximum-likelihood method. In order to attain the confidence levels for the branches of the phylogenetic tree, a bootstrap analysis using 100 replicates was performed. The phylogenetic tree was constructed and visualized using MEGA7 software (Kumar et al., 2016). Moreover, determination of the protein secondary structure was performed using the SOPMA program (Geourjon and Deleage, 1995). In order to determine the three-dimensional structure of *WsCYP85A69*, the Phyre^2^ server (Protein Homology/analogy Recognition Engine V 2.0) was used (Shin et al., 2014). The prediction of the 3D structure was performed using the crystal structure of cytochrome P450 1a2 (PDB: 2hi4) as a template. The prediction of ligand binding sites was performed using the GALAXYWEB server^6^ (Lovell et al., 2002). Subsequently, the ConSurf server tool^7^ was used to predict the structurally, evolutionary and functionally important regions of the deduced amino acid sequences (Celniker et al., 2013).

### Differential Gene Expression Pattern in Different Parts of Plant

To detect the expression levels of the *CYP85* gene in different parts of *Withania*, qRT-PCR was performed. For this analysis, total RNA was isolated from five different parts of the plant viz. stalk, roots, leaf, berries, and inflorescence. Further, first strand cDNA was synthesized using about 1 μg of the total RNA using Revert-aid Premium M-MuLV reverse transcriptase (Fermentas, Burlington, Canada) according to the manufacturer’s instructions. The reaction mixture contained a total volume of 20 μl with 0.2 μl cDNA template, 200 nM each of the primers, and 10 μl SYBR Green Universal Master Mix (Applied Biosystems, United Kingdom). The PCR was performed under the following cycling conditions: one cycle of 95°C for 1 min, 40 cycles of 95°C for 10 s, 60°C for 20 s and 72°C for 20 s. Real time PCR was performed in triplicate with 48-well optical plates using the ABI StepOne Real-time qPCR system (Applied Biosystems, Foster City, CA, United States). Actin gene was used as the standardization control. The obtained results were examined in triplicate and a dissociation curve was used to validate the specificity of each primer pair. The quantitative variation between the replicates were examined using a relative quantification method (2^–ΔΔCT^) (Livak and Schmittgen, 2001). Experiments were repeated thrice each with three technical replicates.

### Quantification of Castasterone Content by HPLC

Extraction of phytosterol from *W. somnifera* was performed using a modified protocol from Ding et al. (2013) and Singh et al. (2017). Briefly, leaf samples were dried at a temperature of 25 ± 2°C and with a relative humidity of 65 ± 5% and ground to a fine powder by using pestle and mortar. The extraction was performed using 2 mL chloroform:MeOH (2:1 v/v) as extraction solvents for 1 h at 75°C followed by evaporation of solvents using a vacuum concentrator. Saponification of dried residue was done in 500 μL of 6% KOH in MeOH at 90°C for 1 h, followed by the cooling of samples at room temperature. Further, a mixture of hexane:water (1:1) was added, mixed properly and centrifuged briefly to separate the phases. Separated hexane-phase was transferred into a clean Eppendorf tube and the remaining aqueous phase was re-extracted using n-hexane. The hexane-phase was dried and resuspended in HPLC grade ethanol and subjected to HPLC analysis. The standards of castasterone (1 mg/ml) were used as a marker and HPLC-grade ethanol was used to dissolve the marker as well as dried extracts (20 mg/ml) and subsequently given to HPLC for quantitative analysis. HPLC analysis was performed with the Shimadzu HPLC system (Shimadzu, Tokyo, Japan) equipped with a 515 quaternary gradient pump, 717 Rheodyne injector, 2996 PDA detector and CLASS-VP software v 6.14. These samples were filtered through 0.45 μM filters (Millipore, Bedford, MA, United States) and were separated on a RP-18e (4.6 × 100 mm, 5 μm) (Merck, Bangalore, India) column. The mobile phase consisted of methanol-water (60:40; v/v) delivered at a flow rate of 0.5 ml/min. The samples were analyzed at 45°C to provide efficiency to the peaks. The UV chromatograms were recorded at 205 nm.

### Construction of Yeast Expression Vector of *WsCYP85A69*

Full length ORF of *WsCYP85A69* was PCR amplified using expression primers harboring restriction sites of the appropriate enzymes (Table 1). The amplified product was successively cloned into the pJET vector and positive colonies were further used for plasmid isolation. The isolated plasmid was first restricted with *Kpn*1 enzymes and purified followed by partial digestion with the *BamH*1 restriction enzyme. The restricted gene was purified and sub-cloned into a pYeDP60 expression vector. Subsequently, the *WsCYP85A69*-pYeDP60 construct was transformed into the *S. cerevisiae* having NADPH: cytochrome P450 reductase gene from *Arabidopsis* engineered for cytochrome expression (Pompon et al., 1996). Transformed yeast colonies were grown on a selection medium, were incubated at 30°C and positive colonies were further used for induction of P450 expression as described in Kim et al. (2005). Yeast cells expressing the P450 protein were harvested by centrifugation and yeast cell walls were broken by zymolyase followed by manually breaking them with 0.45 mm glass beads in 50 mM Tris–HCl buffer (pH 7.5) having 600 mM sorbitol and 1 mM EDTA. The mixture that formed was centrifuged for 10 min. at 10,000 *g* and the subsequent supernatant was subjected to ultra-centrifugation for 90 min. at 100,000 *g*. The pellet which formed contained microsomes which were resuspended in 50 mM Tris–HCl (pH 7.4), 1 mM EDTA and 5% (v/v) glycerol and stored at –80°C whereas the supernatant was discarded. Microsomal protein isolation was performed at 0–4°C and differential spectrophotometry was used to measure their cytochrome P450 content according to Omura and Sato (1964). Further, the quantification of isolated microsomal was performed using the Bradford colorimetric protein assay.

### Enzymatic Assay of *WsCYP85A69*

Oxidase functionality of *WsCYP85A69* was confirmed by scrutinizing product formation using LC-PDA-MS analysis. Typical enzymatic activity was performed in 100 μl volume using phosphate citrate buffer (20 mM; pH 7.4) with the P450 enzyme (isolated microsomal protein) and 6-deoxocastasterone as a substrate and NADPH (0.6 mM) (as cofactor). Consequently, the reaction mixture was incubated at 25°C for 35 min (Kim et al., 2005). The reactions were quenched by acidification (10 μl of 20% HCl) and further extracted with 200 μl ethyl acetate. After centrifugation, the ethyl acetate phase was transferred to a new vial and extraction was repeated with another 200 μl ethyl acetate. These ethyl acetate extracts were evaporated to dryness and re-dissolved in methanol. The reaction was repeated five times.

### LC-PDA-MS Analysis

Products obtained after the incubation of the substrate with the enzyme, were analyzed using LC-PDA-MS. The Shimadzu LC-PDA-MS (ESI) system (Tokyo, Japan) was used for the analysis. The examination was performed on a LiChrospher® RP-18 (4.6 × 250 mm inner diameter, 5 μm) column. The mobile phase A composed of 0.1% (v/v) formic acid in water and mobile phase B composed of acetonitrile, flow rate was kept at 0.5 mL/min and column oven temperature was maintained at 27°C. The analysis was executed in +ESI mode and the scanning of mass ranged from 100 and 1000 amu. Other common MS conditions were as follows: DL temperature 225°C, nebulizer gas flow 3 L/min and drying gas flow were 15 L/min. Total run time was 35 min. 10 μL of the sample volume was injected onto the LC-PDA-MS system. Data were obtained and processed using LabSolutions software. This LC-PDA-MS method was used to determine the presence of castasterone.

### Generation of Over-Expression Construct

Further, to examine the role of *CYP85A69* in brassinosteroids biosynthesis, an over-expression construct was prepared by amplifying the full length ORF of *WsCYP85A69* using expression primers harboring restriction sites of the appropriate enzymes (Supplementary Table S1). The amplified product was successively cloned into the pJET vector and positive colonies were further used for plasmid isolation. The isolated plasmid was restricted with suitable enzymes (*Bgl*II and *Spe*I) and the purified gene was sub-cloned into a GFP expressing pCAMBIA-1302 vector. Subsequently, the *WsCYP85A69*-pCAMBIA1302 construct was transformed into the *A. tumefaciens* for agroinfiltration of *Withania* plants. The bacterial cultures were grown in LB medium harboring desired antibiotics for 48 h until OD_260_ reached 2.0, harvested and resuspended in infiltration buffer [10 mM MgCl2; 10 mM 2-(4-morpholino)-ethane sulfonic acid (MES); 300 μM acetosyringone pH 5.6]. Suspension mixture containing *A. tumefaciens* culture harboring both an empty vector and expression constructs were infiltrated separately into the leaves of *W. somnifera* using a 1 ml needleless syringe. After 72 h of post-infiltration, leaf samples were harvested and processed for quantitative real time analysis and phytochemical evaluation as described above. Wild plants were taken as the control. Experiments were repeated thrice each with three technical replicates.

### Construction of aMIR Constructs

The expression constructs for aMIR constructs were developed to study the effect of suppressed transcript levels of the *CYP85A69* gene on castasterone synthesis. For this, the design of potential aMIRs targeting *WsCYP85A69* was performed using the WMD3 tool hosted at^8^ Briefly, the ORF of *WsCYP85A69* was submitted to the WMD3 tool which then offered a list of several potential aMIRs. Two putative sequences were chosen for the construction of the plant expression vector to express artificial miRNA in *Withania*. Artificial pre-aMIR constructs were synthesized by mutagenizing the backbone of *Arabidopsis thaliana* pre-miRNA159a, and subsequently cloned in the pBI121 vector at the *Xba*I and *Sac*I sites as described by Niu et al. (2006) and Rather et al. (2018). Primers used for PCR mutagenesis as well as amplification are provided in the Supplementary Table S1. Further, pBI121-aMIR1 and pBI121-aMIR2 constructs were transformed into the *A. tumefaciens* which were used for agro-infiltration of *W. somnifera*. The agro-infiltered leaf samples were collected on the third day post-agro-infiltration and subsequently used for qRT-PCR analysis to evaluate the alteration in gene expression levels of *CYP85A69*. Moreover, phytochemical analysis of the samples was done as described above. Experiments were repeated thrice each with three technical replicates.

### Genome Walking Method for Isolation of *WsCYP85A69* Promoter

To study the regulatory components of the *WsCYP85A69* gene, a genome-walking method was used to isolate its upstream promoter region using the Genome Walker Universal Kit (Clontech). Concisely, for retrieving promoter sequence, isolation of genomic DNA from the leaves *W. somnifera* was done using the Wizard Genomic DNA Purification Kit (Promega, Madison, WI, United States) according to the manufacturer’s instruction. Further isolated DNA was digested using four different blunt-ended restriction endonucleases (*Dra*I, *Pvu*II, *EcoR*V, and *Stu*I) in four distinct aliquots for the construction of Genome Walker DNA libraries. For the generation of DNA libraries, digested DNA samples in each set were extracted and ligated to the GenomeWalker AP adaptor (provided with the kit). These libraries were used as a template in the PCR reaction which was performed using a gene specific-out primer in combination with AP1 (provided with the kit) under following thermo-cycling conditions: 7 cycles at 94°C for 25 s and 72°C for 3 min; 35 cycles at 94°C for 25 s, 67°C for 3 min; and at 67°C for 7 min. The resulting amplified product was diluted 10-fold and further used as a template in the nested PCR. This PCR was performed using a gene specific-in primer with AP2 (provided with the kit) under these PCR conditions: 5 cycles at 94°C for 25 s and 72°C for 3 min; 20 cycles at 94°C for 25 s and 67°C for 3 min, and followed by 67°C for 7 min. The products obtained were loaded on 1.2% agarose gel, major bands were purified and ligated into the pMD20-T cloning vector, and the ligation mixture was transformed into the *E. coli* DH5α strain. The positive colonies were confirmed via colony PCR, subjected to plasmid isolation and subsequently sequenced. Sequencing revealed the presence of various *cis*-acting regulatory components upstream of the ATG codon which were identified using the PLACE^9^, AtPAN^10^, and PlantCare^11^ databases.

### Plant Treatments for Elicitor Assays

To scrutinize the effect of abiotic elicitations on the transcript levels of *WsCYP85A69*, growth chamber grown (25 ± 2°C with a 16 h photoperiod with light intensity of 80–100 μM sq m^–1^ sec^–1^, relative humidity: 50–60%) *Withania* plants were sprayed with 0.1 mM MeJA and 0.1 mM ABA in congruence with the MeJA responsive and ABRE elements identified in the promoter region. However, CT (4°C) was given to some plants chosen on the basis of stress related defensive elements present in the promoter region, to study the effect on relative transcript levels of *WsCYP85A69*. Untreated plants were kept as control for CT and plants treated with the same amount of ethanol were kept as controls for MeJA and ABA elicitations. Samples were harvested post-elicitor treatment at different time intervals (3, 6, 12, and 24 h) for qRT-PCR and phytochemical analysis for castasterone quantification. However, CT was also given to some plants to study the effect on relative transcript levels of *WsCYP85A69*. Untreated plants were used as controls. For qRT-PCR analysis, RNA was extracted from all the treated and control samples and was subsequently used for cDNA preparation as discussed above. These cDNA were further used for real-time PCR analysis to study the effects of elicitor treatments. Experiments were repeated thrice each with three technical replicates.

## Results

### Isolation of cDNA Clone of *WsCYP85A69* and *in silico* Analysis

Cytochrome P450 enzymes catalyze diverse functions in both the primary as well as secondary metabolism of plants. Structurally, they are heme-thiolate proteins with iron atoms coordinated to a proximal cysteine and receive electrons from NAD(P)H via a FAD-domain of auxiliary reductase. The present investigation entails the isolation of complete coding sequences of *WsCYP85A69*, using degenerate primers followed by a RACE PCR strategy. A full-length ORF of the *WsCYP85A69* (MK410296) gene included 1,413 bp nucleotides that codes for a protein of 470 amino acids. This sequence was then submitted to BLASTx for a similarity search and revealed similarities with homologs of the *CYP85A1* gene of *Capsicum annum* (GenBank accession number PHT91631.1) *Solanum lycopersicum* (GenBank accession number NP_001234263.1) and *Nicotiana tabacum* (GenBank accession number NP_001312136.1). This analysis revealed that the *CYP85A69* gene from *W. somnifera* is a homolog of the *CYP85A1* gene and showed a close resemblance with the same gene of other plant species. Furthermore, ConSurf server depicted the presence of various conserved residues in *WsCYP85A69* (Supplementary Figure S1). Furthermore, the secondary structure of *WsCYP85A69* was also predicted using the Self-Optimized Prediction Method with Alignment (SOPMA) online tool. It showed that *WsCYP85A69* is present predominantly in α-helical form with a respective percentage of 51.3% of random coils (30.12%), whereas β-turns (4.76%), and extended strands (10.82%) were also observed (Supplementary Figure S2A). PROSITE sequence analysis revealed the presence of cytochrome P450 cysteine heme-iron ligand signature sequence at 407–416 amino acid position (Supplementary Figure S2A). This sequence pattern was found to be FGGGTRQCPG rich in unique amino acid residues and was present toward C-terminus. This typical sequence was also displayed when the same gene from taxonomically diverse species were aligned using multiple sequence alignment tools. Further, active residues in ligand binding sites were predicted using the GALAXY web server and displayed as I^112^, H^120^, M^244^, T^269^, L^270^, S^273^, T^277^, E^340^, V^344^, R^346^, L^403^, F^404^, R^409^, C^411^, P^412^, G^413^, L^416^, G^417^ (Supplementary Figure S2). These entire features substantiate that *WsCYP85A69* belongs to the cytochrome P450 superfamily which mediates the biosynthesis of various secondary metabolites.

### Phylogenetic Analysis of *WsCYP85A69*

**FIGURE 2:**
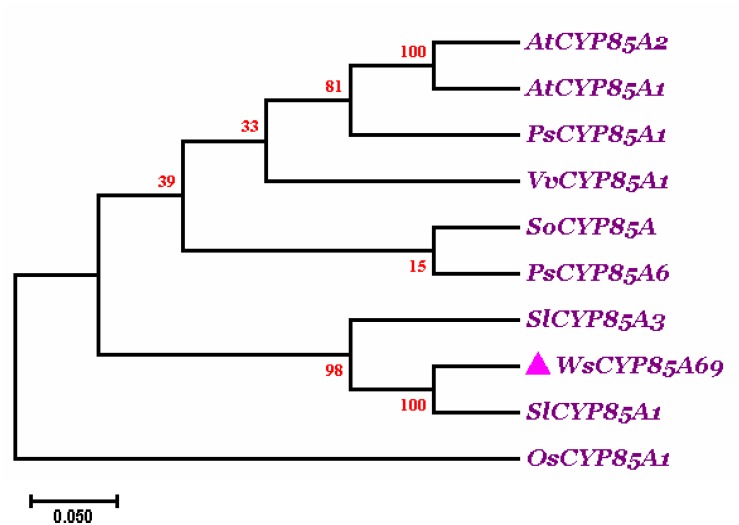
Phylogenetic tree of *WsCYP85A69*. The phylogenetic analysis was executed using the ClustalW program as well as MEGA7 software. The numbers on the nodes indicate the bootstrap values after 100 replicates. The bar represents an evolutionary distance of 0.05%. Poisson correction method was used to compute the evolutionary distances. The analysis was performed by aligning *CYP85A* amino acid sequences chosen by scrutinizing available data related to characterized *CYP85A* gene from different plant species from NCBI database. Sequences were selected based on the complete coding sequence. The tree was rooted using homolog of *CYP85A1* gene from *Oryza sativa* as an outgroup, seeing that it belongs to the Poaceae family. The accession numbers of used plant *CYP85A* sequences are as follows: *Withania somnifera* (*WsCYP85A69*: MK410296); *Arabidopsis thaliana CYP85A1* (AT5G38970.1); *A. thaliana CYP85A2* (AT3G30180.1); *Solanum lycopersicum* (NP_001234263.1; homolog of *CYP85A1*); *Solanum lycopersicum* (NP_001234520.1; CYP85A3); *Oryza sativa* (AC092778.2; homolog of *CYP85A1)*; *Vitis vinifera* (DQ235273.1; homolog of *CYP85A1)*; *Pisum sativum* (AB218759; homolog of *CYP85A1*); *Pisum sativum* (AB218760; *CYP85A6*); *Spinacia oleracea* (KT900949; homolog of *CYP85A1*).

Phylogenetic analysis of *WsCYP85A69* was performed with characterized *CYP85A* from other plant species to elucidate the degree of evolutionary relatedness. *CYP85* amino acid sequences belonging to different plant species were retrieved from the National Centre for Biotechnology Information (NCBI) database (Supplementary Figure S4). These sequences were subjected to multiple alignments using ClustalW followed by phylogenetic analysis using Mega 7.0 software. A phylogenetic tree was rooted using *Oryza sativa* of the Poaceae family as an outgroup. The phylogenetic tree displayed that *CYP85A69* of *W. somnifera* falls within the same clade as *CYP85A1* of *Solanum lycopersicum*, revealing their orthologous nature. Moreover, *SlCYP85A3* may plausibly have evolved as a multifunctional enzyme due to duplication events during evolutionary processes resulting in its early divergence from the close orthologs (Figure 2). These results support the functional relatedness of *WsCYP85A69* with other functionally characterized *CYP85* genes.

### Analyses of Expression Pattern of *WsCYP85A69* and Castasterone Accumulation

**FIGURE 3:**
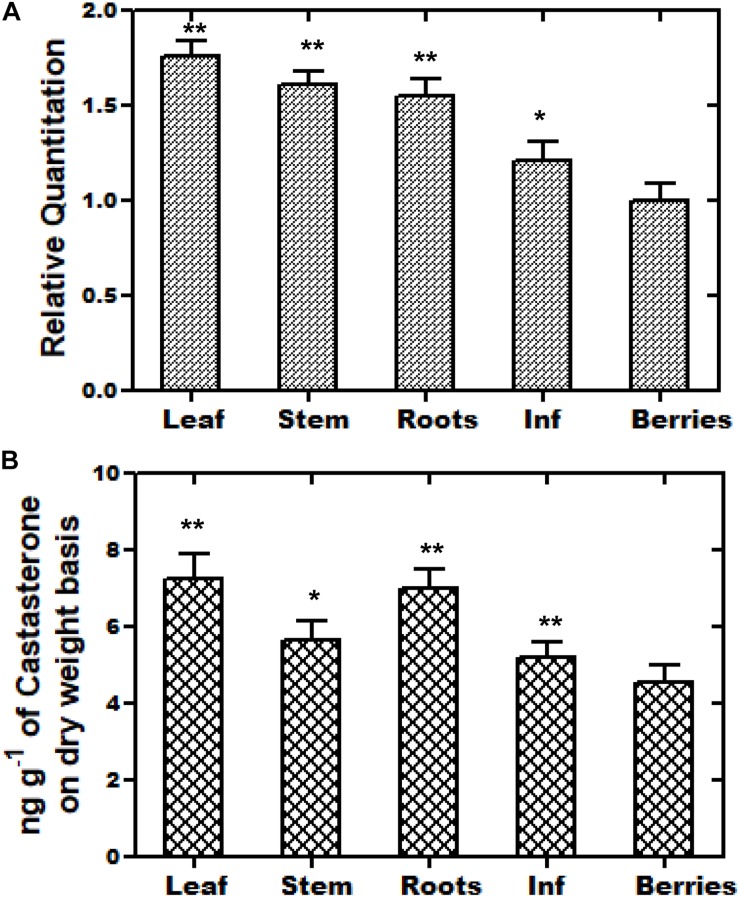
Tissue-specific real-time expression analysis and HPLC analysis of castasterone. **(A)** Quantitative assessment of the expression levels of *WsCYP85A69* in different plant parts of *W. somnifera* viz. leaf, stalk, roots, inflorescence (abbreviated as Inf) and berries were performed using quantitative real-time PCR (qRT-PCR). For the normalization of the expression of *WsCYP85A69*, β-Actin was used as an endogenous control and berries were used as internal control. Values are means ± SE of three independent biological replicates each with three technical replicates. Standard errors (SE) are represented by bars. Differences were scored as statistical significance at ^∗∗^*P* < 0.01 and ^*^P < 0.05 levels. Asterisks indicate the comparison of expression levels of *WsCYP85A69* in berries with other plant parts. **(B)** HPLC analysis of castasterone production in different parts of *W. somnifera* plants viz. leaves, stalk, roots, inflorescence and berries. The contents were higher in young leaves followed by stalk, roots, inflorescence. Berries showed the least levels and were used as control. Values are means ± SE of three independent biological replicates each with three technical replicates. SE are represented by bars. Differences were scored as statistical significance at ^∗∗^*P* < 0.01 and ^*^P < 0.05 levels. Asterisks indicate the comparison of castasterone content in berries with other plant parts.

*The WsCYP85A69* gene was further investigated at the transcriptional level to determine its role in the differential accumulation of brassinosteroids (castasterone). Using relative qRT-PCR, the expression pattern of *WsCYP85A69* was performed in five different tissues viz. leaf, stalk, inflorescence, berries, and the root. All the scrutinized samples showed the distinctive expression pattern of *WsCYP85A69*. Juvenile leaves showed the highest expression level of *WsCYP85A69* followed by the stalk, roots and inflorescence, while berries exhibited the least expression (Figure 3A). In addition to this, phytochemical analysis revealed that young leaves accumulated the highest amount of castasterone in comparison to the stalk, roots and inflorescence, whereas berries showed the least (Figure 3B and Supplementary File S2a).

### Characterization of CS Synthase Activity of BR C6-Oxidase Enzyme by LC-PDA-MS

**FIGURE 4:**
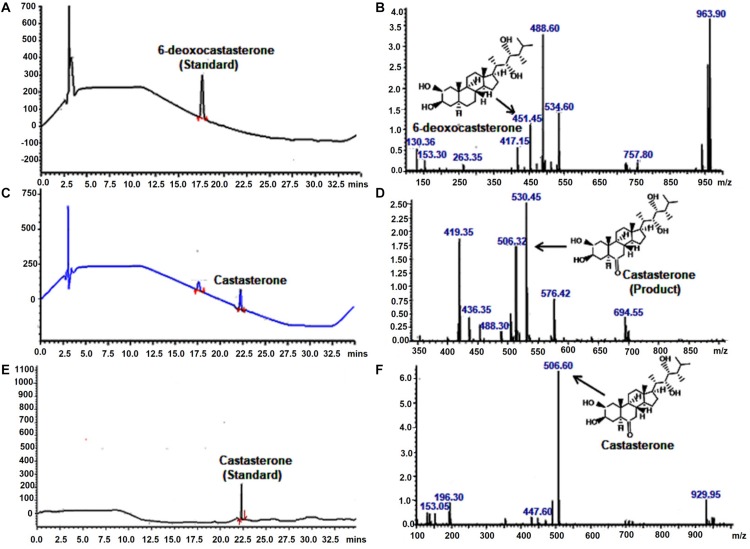
LC-PDA-MS analysis of the product formed by enzymatic activity of C-6 oxidase activity of *WsCYP85A69*. Liquid chromatography equipped with photo-diode array detection (LC-PDA) chromatogram **(A)** and mass spectrometry spectra **(B)** of authentic standard 6-deoxocastasterone which was eluted at retention time of 17.61 min with mass m/z 451.45 LC-PDA **(C)** and mass spectrometry **(D)** spectra of product formed (castasterone) by the enzymatic activity of C-6 oxidase (*WsCYP85A69*), eluted at retention time of 22.1 with mass m/z 506. *In vitro* reaction was performed by incubating microsomes (containing *WsCYP85A69* protein) with 6-deoxo-castasterone (as substrate) and NADPH (0.6 mM) (as cofactor) at 25°C for 35 min in potassium citrate buffer. Further, reaction was stopped by adding HCl and product was extracted twice with ethyl acetate, evaporated to dryness, finally mixed with methanol and subjected to LC-PDA-MS analysis LC-PDA chromatogram **(E)** and mass spectra **(F)** of authentic standard castasterone eluted at retention time of 22.17 with mass m/z 506 the reaction was repeated five times.

To investigate the catalytic function of *WsCYP85A69*, its ORF was cloned into a pYeDP60 vector and transformed into a *S. cerevisiae* WAT11 strain with *Arabidopsis* NADPH P450 reductase 1. It was further expressed in a yeast medium (YPEG) under the control of a galactose inducible promoter. Expression was observed at various time intervals and the maximum expression level was observed at 1 M galactose for 18 h at 30°C. This optimum expression level was then chosen for the isolation of microsomes as described in Pompon et al. (1996). Microsomes were purified from transformed yeast and the *in vitro* reaction was performed by incubating microsomes with 6-deoxo-castasterone at 25°C for 35 min in a potassium citrate buffer. Furthermore, the reaction was stopped by adding HCl and the product was extracted twice with ethyl acetate, evaporated to dryness, finally mixed with methanol and subjected to LC-PDA-MS analysis (Kim et al., 2005). LC-MS analysis of the reaction product showed the presence of castasterone which was eluted at the retention time of 22.19 min with a calculated mass of 506 [M+ACN]^+^ (Figures 4C,D). These results functionally validate the C-6 oxidase activity of *WsCYP85A69* as it efficiently converted 6-deoxocastasterone (substrate) to castasterone (product). However, no activity was observed in the yeast transformed with an empty vector as the control.

### Transient Over-Expression of *WsCYP85A69* Up-Regulates Castasterone, Stigmasterol, and Withanolides Accumulation

**FIGURE 5:**
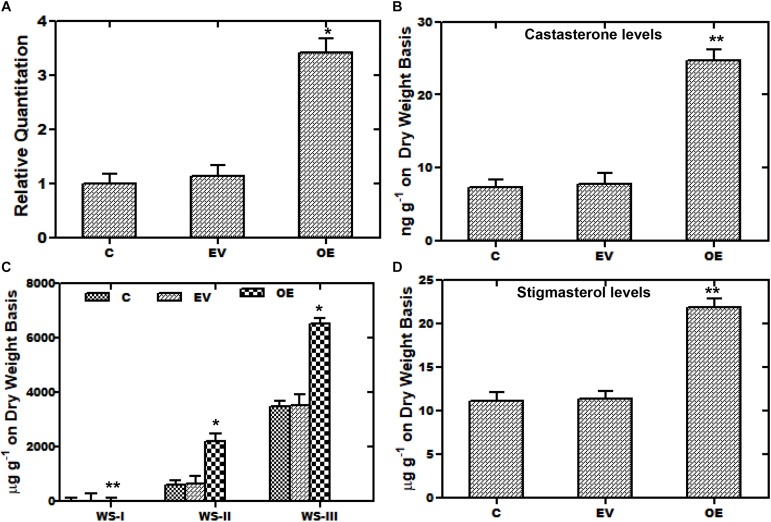
Transient over-expression assay of *WsCYP85A69.* **(A)** Quantitative real-time expression of *WsCYP85A69* gene showing enhanced transcript levels of *WsCYP85A69* in infiltered leaves (OE) compared to control (C). Obtained values are compared and analyzed with one-way ANOVA using GraphPad Prism 6 software. Values are means ± SE of three independent biological replicates each with three technical replicates. SE are represented by bars. Differences were scored as statistical significance at ^∗∗^*P* < 0.01 and ^*^P < 0.05 levels. Asterisks show the difference between infiltered and control plants. **(B)** HPLC analysis of castasterone in leaves infiltered with *WsCYP85A69*-pCAMBIA1302 showed increased castasterone content compared to control **(C,D)** HPLC analysis of transformed leaves for elevated levels of withanolides and stigmasterol, respectively. The levels of metabolites in transformed leaves were compared to those in the control plants. Obtained values were compared and analyzed with one-way ANOVA using GraphPad Prism 6 software. Values are means ± SE of three independent biological replicates each with three technical replicates. SE are represented by bars. Differences were scored as statistical significance at ^∗∗^*P* < 0.01 and ^*^P < 0.05 levels. Asterisks show the difference between infiltered and control plants.

A transient over-expression assay was performed to investigate the role of *WsCYP85A69* in the biosynthesis of steroids and withanolides. For this analysis, leaves of *W. somnifera* were agro-infiltered with the *A. tumefaciens* Gv3101 strain harboring an empty pCAMBIA1302 vector. *WsCYP85A69* was fused toward the 5′-terminal of the green fluorescent protein (GFP) gene in the pCAMBIA1302 vector to form a *WsCYP85A69- pCAMBIA1302* construct, under the control of the 35S-CaMV promoter (Supplementary Figure S5J). The third day after post-infiltration, the transformed leaf samples were harvested for GFP detection, quantitative RT-PCR and phytochemical analysis. Expression of *WsCYP85A69*-GFP in infiltered leaves was confirmed by fluorescent microscopy (Supplementary Figures S5A–I). Moreover, qRT-PCR examination showed 2.42-fold increase in *CYP85A69* transcript levels in infiltered leaf samples harvested after the third day (Figure 5A). Furthermore, chemo-profiling of infiltered leaves presented a substantial increase in castasterone content. Transformed leaves over-expressing the *WsCYP85A69* gene showed a 2.3-fold increase in castasterone levels determined by HPLC (Figure 5B and Supplementary File S2b). Additionally, the effect of the increased expression of the *WsCYP85A69* gene was studied on end products of other triterpenoids i.e., withanolides and phytosterol. HPLC analysis displayed a 2-fold increase in WS-I, 2.69-fold increase in WS-II- and 0.86-fold increase in WS-III contents compared to the control (Figure 5C and Supplementary File S4a). However, leaves infiltered with *Agrobacterium* containing pCAMBIA1302 (empty vector) showed a slight increase in withanolides accumulation. It could be due to the biotic stress induced on plants during agroinfiltration. Moreover, the increased *WsCYP85A69* gene also had an impact on the stigmasterol accumulation as it showed a 0.9-fold increase in its levels (Figure 5D and Supplementary File S3a). Over-expression of the *WsCYP85A69* gene suggested that increased concentrations of brassinosteroids may have a cascading effect on the modulation of multiple pathway genes leading to the increased sterol precursors and thus the enhanced production of stigmasterol and three of the withanolides namely, WS-I, WS-II, and WS-III.

### Artificial Micro RNA (aMIR) Constructs of *WsCYP85A69* Resulted in Decreased Castasterone, Stigmasterol, and Withanolides Content

**FIGURE 6:**
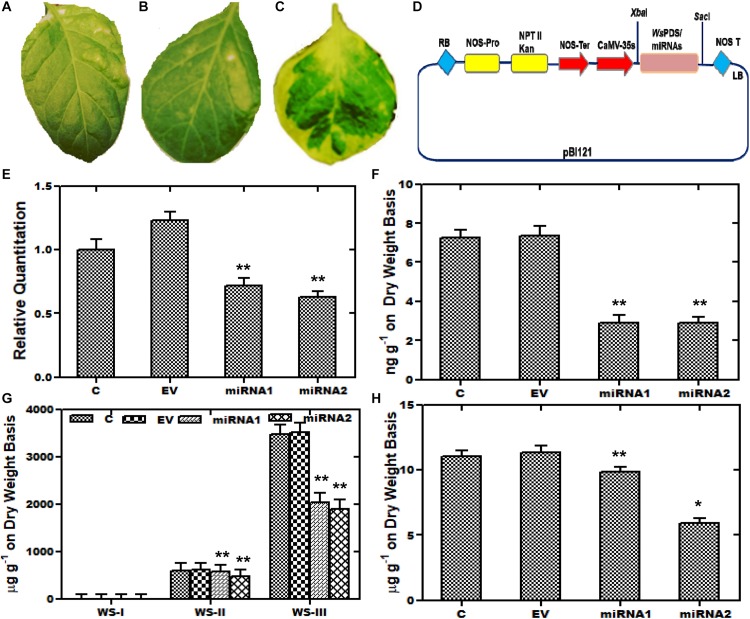
Artificial micro-RNA mediated silencing (aMIR) of *WsCYP85A69.* **(A–C)** Phytoene desaturase was used as marker whose silencing resulted in white spots on infiltered leaves. **(D)** Vector map showing PDS-pBI121/aMIR-pBI121 **(E)** aMIR1-*WsCYP85A69* and aMIR2-*WsCYP85A69* constructs infiltered in leaves illustrating reduced expression levels of *WsCYP85A69* compared to control after third day of post-agro-infiltration. Values are means ± SE of three independent biological replicates each with three technical replicates. SE are represented by bars. Obtained values were compared and analyzed with one-way ANOVA using GraphPad Prism 6 software. Differences were scored as statistical significance at ^∗∗^*P* < 0.01 and ^*^P < 0.05 levels. **(F)** HPLC analysis of castasterone levels in agro-infiltered leaves showed decrease in its content compared to control. **(G)** Phytochemical analysis of reduced levels of withanolides in transformed leaves via HPLC **(H)** HPLC analysis of stigmasterol levels in infiltered leaves also showed decreased stigmasterol content compared to control. The contents of metabolites in transformed leaves were compared to those in the control plants. Obtained values were compared and analyzed with one-way ANOVA using GraphPad Prism 6 software. Values are means ± SE of three independent biological replicates each with three technical replicates. SE are represented by bars. Differences were scored as statistical significance at ^∗∗^*P* < 0.01 and ^*^P < 0.05 levels. Asterisks show the difference between infiltered and control plants.

Furthermore, aMIR mediated silencing of *WsCYP85A69* was also performed to confirm its functional role in triterpenoids biosynthesis. For the establishment of aMIR mediated silencing in *W. somnifera*, four-leaf-staged plants grown in a growth chamber, were used. Since phytoene desaturase is extensively used as a marker in silencing studies, in our present study, a 200-bp PDS fragment was therefore cloned into *XbaI* and *SacI* sites of the pBI121 vector to generate a pBI121-*Ws*PDS construct. This construct was also transformed into an *A. tumefaciens* and agro-infiltered in *W. somnifera* leaves. These infiltered plants showed a photo-bleaching phenotype and appeared in varied patches distributed on the leaf surface. In maximum cases, leaves displayed a mild phenotype as albino and green patches and stunted growth. In PDS-pBI121 infiltered plants, repression of the green pigment was started with white patches and distributed on the whole leaf clearly showing the inhibition (Figures 6A–C). After the successful infiltration of *Ws*PDS-pBI121 in leaves (Figures 6A–C), *CYP85A69*-aMIR1 and *CYP85A69*-aMIR2 were also generated and infiltered into leaves (Figure 6D). Samples were harvested after the third day post-infiltration for qRT-PCR analysis vis-à-vis castasterone evaluation. qRT-PCR analysis showed that the aMIR2 construct was the most effective in down regulating the transcript levels of *WsCYP85A69* compared to aMIR1. The aMIR1 construct showed a 0.28-fold reduction in the *WsCYP85A69* transcript levels whereas, aMIR2 showed a 0.37-fold reduction in transcript levels of *WsCYP85A69* at the third day post-infiltration, compared to the control (Figure 6E and Supplementary File S2c). Furthermore, a phytochemical evaluation of aMIRs infiltered leaves was also performed to explicate the effect of reduced levels of *WsCYP85A69* transcript on castasterone accumulations. aMIR1 displayed a 0.594-fold whereas aMIR2 showed a 0.62-fold decrease in castasterone levels (Figure 6F). Moreover, to study the effect of the decreased *CYP85A69* gene on other metabolites accumulation, an HPLC analysis was performed. Chemo-profiling displayed 0.1- and 0.2-fold decrease in WS-I, 0.04 and 0.211-fold in WS-II and 0.41- and a 0.45-fold decrease in WS-III content (Figure 6G and Supplementary File S4b). Similarly, chemo-profiling analysis also showed a 0.16- and 0.48-fold reduction in stigmasterol content at the third day post-infiltration (Figure 6H and Supplementary File S3b). Inclusively, the over-expression analysis along with aMIR mediated downregulation of *WsCYP85A69* strongly suggests its significant regulatory role in castasterone and withanolides biosynthesis.

### Isolation of the Promoter Region and Identification of *cis*-Regulatory Elements

In order to study the transcriptional regulation of *WsCYP85A69*, its 5′ upstream flanking regions of 610 bp (GenBank accession no. MK611931) was isolated using a genome walking approach. Further, the upstream region was analyzed to predict the location of the transcription initiation site which was found to be positioned at −84 bp upstream of the ATG initiator codon, whereas putative TATA box was shown to be located −35 bp upstream of the transcription initiation. *In silico* investigation of the promoter region was performed using PLACE and PlantCare databases, which revealed the presence of several significant *cis*-acting regulatory components (Supplementary Figure S6 and Table 1). These embraced the MYC binding site, three MYB binding sites, three WRKY-boxes, a GA_3_ responsive element, light-responsive, hormone-responsive elements and various other stress-related elements. Altogether, our analysis suggested that the transcriptional regulation of the *WsCYP85A69* expression might be mediated by different transcription factors, which integrates different spatial and temporal cues including severe environmental conditions.

### Elicitation Studies on *WsCYP85A69* Expression vis-à-vis Castasterone Biosynthesis

**FIGURE 7:**
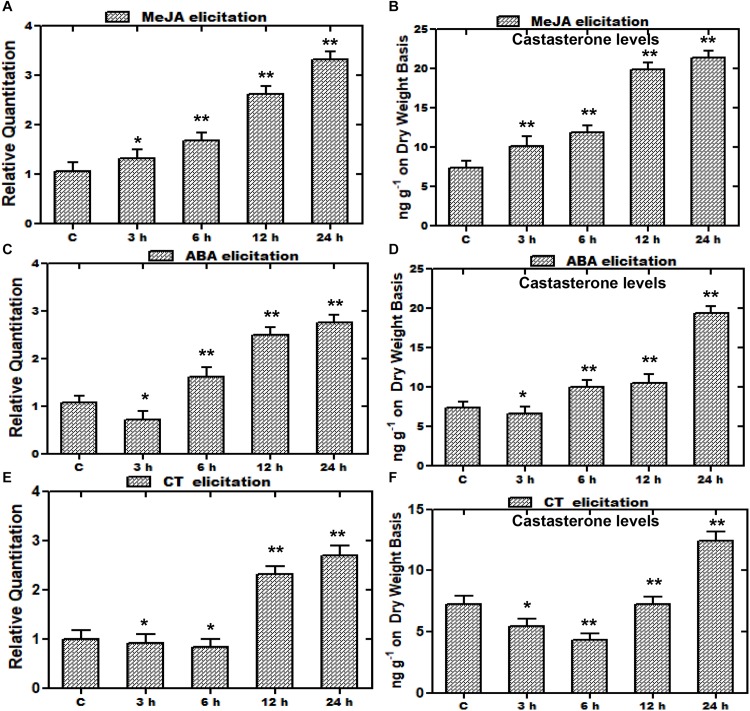
Effect of elicitor treatments on transcript profiles of *WsCYP85A69* and castasterone accumulation at different time intervals. Time course expression profiling of *WsCYP85A69* and castasterone accumulation pattern in response to elicitations by 0.1 mM methyl jasmonate (MeJA) **(A,B)**; 0.1 mM abscisic acid (ABA) **(C,D)** and cold treatment (CT) **(E,F)**. Actin was used as an endogenous control. Values are means ± SE of three independent biological replicates each with three technical replicates. SE are represented by bars. Obtained values were compared and analyzed with one-way ANOVA using GraphPad Prism 6 software. The time-course accumulation of castasterone was statistically significant at ^∗∗^*P* < 0.01 and ^*^P < 0.05 levels. Asterisks show the difference between elicitor-treated and control plants.

In recent years, there has been significant interest in the effect of various elicitors on gene expressions, and many of the studies have been conducted on elicitations to prompt variations in metabolite production. Such studies entail the exclusive indication about the way, induction or repression of a gene is executed by various elicitors (Zhao et al., 2005). Furthermore, an increase in the expression of various biosynthetic genes by such elicitors leads to enhanced metabolite production for better survival persistence and competitiveness of a plant. Consequently, plants subjected to stress including elicitation or signaling molecules leads to the increased accumulation of metabolites in plants (Thakur and Sohal, 2013; Singh and Dwivedi, 2018). Elicitors selected on the basis of presence of the MeJA responsive element and stress related defensive elements were assessed with regard to the *WsCYP85A69* expression pattern vis-à-vis castasterone levels to understand its regulatory role. Treatments were subjected to 1.5-month-old greenhouse grown plants and the harvesting of treated samples was performed after 3, 6, 12 and 24 h intervals. Plants treated with equal amounts of water and ethanol, were kept as the control. In addition, earlier findings in *Spinacia oleracea* showed that ABA, CT, and PEG1000 elicitations resulted in altered metabolite accumulation (Duan et al., 2017). Therefore, a similar attempt was executed in *W. somnifera* by exogenous application of MeJA, CT, and ABA as elicitors to examine their effect on the expression of *WsCYP85A69*. Following various treatments, the transcript level of *WsCYP85A69* was assessed through quantitative RT-PCR vis-à-vis metabolite analysis by HPLC at different time intervals post-treatment (3, 6, 12, and 24 h) (Figures 7A–C and Supplementary File S2d). After examining all the treated samples, MeJA was considered to be the pre-eminent inducer of *WsCYP85A69* expression levels as it led to a 0.3–2.3-fold increase in *WsCYP85A69* transcript levels up to 24 h concomitant with a 0.38–1.92-fold increase in castasterone content (Figures 7A,B). Additionally, ABA treatment initially showed a drop in mRNA levels up to 3 h, followed by an upsurge in *WsCYP85A69* expression levels up to 24 h (0.6–1.75) (Figure 7C). The same deviation was also concordant with the gradual increase in castasterone content up to 24 h (1.65-fold) (Figure 7D). Similarly, CT also led to the gradual decline in *WsCYP85A69* transcription up to 6 h with a significant increase in expression up to 24 h (1.7-fold) (Figure 7E) with corresponding increase in castasterone (0.69-fold) (Figure 7F). Consequently, in our study, MeJA elicitation resulted in a higher accumulation of castasterone when compared with the effect of ABA and CTs.

## Discussion

Plants synthesize a diverse array of polyhydroxylated steroidal compounds which are regarded as a sixth plant hormone. They play fundamental roles in controlling the activity of various metabolic pathways and also regulate the overall plant growth and development processes leading to morphogenesis (Saini et al., 2015; Tang et al., 2016). Therefore, keeping in view the importance of brassinosteroids, we have targeted one of the pivotal genes of the brassinosteroids biosynthetic pathway, BR C-6 oxidase, *CYP85A69*, which catalyzes the conversion of deoxocastasterone to castasterone. This gene is a homolog of *CYP85A1* from *Arabidopsis* and we have successfully cloned and characterized it to scrutinize its role in the brassinosteroid biosynthetic pathway. The functional validation, quantitative real-time expression profiling in corroboration with metabolite evaluation, confirmed the oxidative nature of *WsCYP85A69*.

Recent development in computational techniques has become a significant tool for the prediction of the structure and function of a protein that further helps in the metabolic engineering of its biosynthetic pathway (Westfall et al., 2012). Consequently, the present work also entails the bio-informatic analysis for the interpretation of the catalytic and ligand binding sites. The presence of a cysteine heme-iron ligand signature, FGGGTRQCPG, which is a characteristic signature of P450 sequences, has been confirmed by the PROSITE sever tool. The heme sequence was discernible at the 407–416 amino acid position (Supplementary Figure S3). Furthermore, like all eukaryotic monooxygenases, a series of amino acid residues that assist in anchoring monooxygenase to the endoplasmic reticulum membrane was identified toward the N-terminal. This anchor region is crucial for normal interaction of P450s with their redox partners. Additionally, phylogenetic clustering grouped *WsCYP85A69* in the same clade with a homolog of the *CYP85A1* gene from *S. lycopersicum*, depicting their close homology.

Furthermore, recent development in the field of metabolic engineering and expression systems have paved a way for the characterization of key pathway genes which further enable the fine-tuning of biosynthetic pathways for improved efficiency and their reconstruction in heterologous hosts (Ehrenberg, 2015). Accordingly, in the synthesis of high value bioactive metabolites including amorphadiene (Lau and Sattely, 2015) opiates (Kim and DellaPenna, 2006) and aglyconic etoposide (Singh et al., 2014) in homologous and/or heterologous hosts, various combinatorial biosynthetic approaches have been used. With this viewpoint, an ingenious yeast expression system was used for efficacious expression as well as functional characterization of *WsCYP85A69* in heterologous host *S. cerevisiae*. Functional validation of *WsCYP85A69* in *S. cerevisiae* revealed its oxidative functionality. The LC-MS profile of the reaction product was similar to its authentic standard, (Figures 4E,F), displaying a peak whose retention time was similar to that of the standard (Figures 4C,D). These results clearly demonstrate that *WsCYP85A69* encode proteins committed for biosynthesis of the defined product and are thus favorable gene targets for future pathway engineering endeavors.

*CYP85A1* performs the oxidation of deoxocastasterone at the C-6 position to synthesize castasterone (Figure 1). *CYP85A1* has been reported to be a rate limiting enzyme in the biosynthesis of brassinosteroids which play a significant role in growth and development and are thus an important target for metabolic engineering (Tiwari et al., 2014). With this viewpoint, a transient over-expression assay was performed to monitor its effect on the secondary metabolism of *W. somnifera*. In the present study, the transient expression of *WsCYP85A69* in a leaf showed significant enhancement of mRNA transcript levels (2.42-fold) along with a 2.3-fold increase in castasterone content (Figures 5A,B). However, increased expression of the *WsCYP85A69* gene also had an impact on the accumulation of other triterpenoids. It resulted in a 2-fold increase in WS-I, a 2.69-fold increase in WS-II content, and a 0.86-fold increase in WS-III in comparison to the control (Figure 5C). Interestingly, increased *WsCYP85A69* transcript levels also have an impact on stigmasterol accumulation as it led to a 0.9-fold increase in stigmasterol content in comparison to the control (Figure 5D). The observed metabolic response is plausibly due to the synchronization and interaction between multiple biosynthetic machineries. The coordinated expression of various genes of specific pathways regulates the synthesis of various metabolites and this could serve as a prognostic tool for biotechnological interventions. Furthermore, the metabolic networking in nearly all organisms is quite large and complex and therefore, models of metabolic networks are needed to identify specific genes or transcription factors that drive various metabolic responses.

In the post-genomic era, amiRNAs mediated gene silencing has been used as a potent reverse genetic tool to study gene functions. It is a robust technique which could be used to unravel new insights of gene functions and to manipulate metabolic pathways in very short time, compared to conventional methods (Hernandez-Garcia and Finer, 2014). For example, in *N. tabacum*, silencing of the *Nt*FLS (flavonol synthase) gene resulted in an alteration of flavonoid biosynthesis (Mahajan et al., 2011). Because of the affectivity of amiRNA technology along with its minimum off-targets attribution, it was used to suppress the *WsCYP85A69* gene expression in order to elucidate its effect in castasterone accumulation. For the induction of gene silencing, two silencing constructs of *WsCYP85A69*-aMIRs (*WsCYP85A69*-aMIR1 and *WsCYP85A69*-aMIR2) in pBI121 were generated and agro-infiltrated in the leaves of *W. somnifera.* Our results presented that *WsCYP85A69*-aMIR2 exhibited the highest suppression of *WsCYP85A69* transcript levels (0.37-fold) as compared to *WsCYP85A69*-aMIR1 (0.28-fold) (Figure 6E). Further, these results were corroborated with a phytochemical analysis of castasterone contents that also demonstrated their low accumulation (0.6-fold) in transformed leaves as compared to the control (Figure 6F) depicting the crucial role of *WsCYP85A69* in castasterone biosynthesis. However, the silenced *CYP85A69* gene also had an impact on withanolides and stigmasterol accumulation (Figures 6G,H) showing the complexity of the regulation of these pathways through various factors. These results suggest that the reduction of castasterone levels may result in decreased amounts of withanolides, demonstrating their application in studying withanolides biosynthesis in *W. somnifera*. Therefore, it seems that the suppression of the *WsCYP85A69* gene in *W. somnifera* might result in the depletion of sterol precursors that are required for withanolides biosynthesis. It certainly demonstrates the pivotal role of *WsCYP85A69* in the significant turnover of withanolides.

To understand the regulation of gene expressions, promoter isolation and analysis is significant as it has the potential to provide the useful information regarding the activation and suppression of gene expressions in response to various developmental and environmental cues (Dhar et al., 2014). This could open more ways for the use of available promoters in plant biotechnology to increase the production of metabolites by altering gene expression. In our examination, various *cis*-acting regulatory elements were identified to be present in the promoter region of *WsCYP85A69* (Table 1). Further, to elucidate the inducible/repressible nature of the *WsCYP85A69* promoter, various *cis*-regulatory elements were analyzed and subsequently used to assay the modulation in relative transcript levels of *WsCYP85A69*, along with a change in the metabolic flux (Bhat et al., 2012; Dhar et al., 2014). MeJA has been known to be a key regulator of defense as well as developmental processes and plays an important role in improving the transcriptional machinery of plant metabolism, thereby leading to intensive augmentation in the expression of genes of specific metabolic circuits (Ahmad et al., 2016). Scrutinizing the elicitor treated plant samples showed that the expression levels were significantly increased after 6 h and reached the maximum at 24 h post MeJA treatment (up to 2.3-fold) (Figure 7A). Our findings are in agreement with the transcriptional levels of *WsCYP76A* and *WsCYP98A* in *W*. *somnifera* whose expressions were strongly induced in response to MeJA treatment (Rana et al., 2014). However, during the low temperature and ABA stress treatment, its expression levels initially decreased, followed by a 1.75- and 1.7-fold increase respectively, compared to the control (Figures 7C,E). These are in agreement with *SoCYP85A1* from *S. olcerata*, whose expression levels follow the same pattern (Misra et al., 2010).

## Conclusion

In conclusion, the key pathway gene of brassinosteroids, *WsCYP85A69*, from *W. somnifera* has been isolated and functionally validated in the *S. cerevisiae* WAT11 strain. Moreover, its oxidative functionality as well as catalytic potential has been confirmed using LC-PDA-MS and further corroborated through a bio-informatic analysis. These findings have implications to increase the metabolite levels, homologously in *W. somnifera*. In addition to this, a transient over-expression assay ensued elevation in expression levels concomitant with an increase in castasterone, stigmasterol and WS-I, WS-II, WS-III levels. Also, aMIR of *WsCYP85A69* led to the impairment in its normal functioning and resulted in reduced production of all these metabolites. These non-complementary approaches, involving over-expression as well as silencing studies, confirmed the functional contours of *WsCYP85A69* and further directed an explicit understanding of its biosynthetic role vis-à-vis castasterone biosynthesis. Furthermore, the variation in castasterone levels in different tissues were concurred with the gene expression levels of *WsCYP85A69*, depicting a positive relationship of metabolite production with gene expression patterns. Additionally, an analysis of isolated promoter elucidated the presence of several potential upstream *cis*-regulatory elements that facilitated better insights regarding its regulation. Moreover, elicitor studies revealed MeJA as a potent inducer of *CYP85A69* expression leading to a 2.3-fold increase in its relative mRNA levels which corroborated well with the increased metabolic levels. Thus, molecular and functional characterization of *WsCYP85A69* provides a fresh prospective for the manipulation/modulation of the increased production of metabolites in *W. somnifera*.

## Author Contributions

SL and MD conceived and designed the study. AS and GR performed the experiments. SL, MD, and PM analyzed the data. SL and PM contributed the reagents, materials, and analysis tools. AS prepared the original draft. SL, MD, and PM improved the content and edited the manuscript.

## Conflict of Interest Statement

The authors declare that the research was conducted in the absence of any commercial or financial relationships that could be construed as a potential conflict of interest.

## Footnotes

1 http://www.ncbi.nlm.nih.gov

2 http://www.expasy.ch/tools/dna.html

3 http://www.ebi.ac.uk

4 http://www.ncbi.nlm.nih.gov/BLAST/

5 http://www.ebi.ac.uk/Tools/msa/muscle

6 http://galaxy.seoklab.org/index.html

7 http://consurf.tau.ac.il/

8 http://wmd.weigelworld.org/cgi-bin/mirnatools.pl

9 http://www.dna.affrc.go.jp/PLACE/

10 http://atpan.itps.ncku.edu.tw/index.php?id=promoter_analysis

11 http://bioinformatics.psb.ugent.be/webtools/plantcare/html/

## Supplementary Material

FIGURE S1 Prediction of conserved amino acid residues for *WsCYP85A69.* The analysis of conserved residues for *WsCYP85A69* was done using ConSurf and ConSeq web servers. Conservation scale used for residues analysis, from variable to conserved region, is shown in- blue (1) to purple colors (9). Abbreviations used are: e = An exposed residue according to the neural-network algorithm; b = buried residue according to the neural-network algorithm; f = predicted functional residue (highly conserved and exposed); s = predicted structural residue (highly conserved and buried); and X = insufficient data, the calculation for this site was performed on less than 10% of the sequences.

FIGURE S2 Three-dimensional model and ligand-binding site prediction for *WsCYP85A69*. **(A)** Display of ribbon model of three-dimensional structure of *WsCYP85A69* predicted via Phyre2 web server, using crystal structure of lanosterol 14-alpha Demethylase as template for modeling by achieving 100% coverage score, **(B)** ligand-binding sites (zoom view) as predicted by GALAXY web server displays the presence of heme-binding site and presence of I^112^, H^120^, M^244^, T^269^, L^270^, S^273^, T^277^, E^340^, V^344^, R^346^, L^403^, F^404^, R^409^, C^411^, P^412^, G^413^, L^416^, G^417^ residues in its ligand binding sites.

FIGURE S3 Multiple sequence alignment of amino acid sequence of *WsCYP85A69* with its homologs. T-COFFEE web server was used to align the sequences and gave a score of 99. Consensus residues are shaded in maroon color. Sequences used were from *Withania somnifera* (*WsCYP85A69*: MK410296), *Capsicum annuum* (PHT91631.1), *Nicotiana tabacum* (NP_001312136.1), *Solanum tuberosum* (NP_001234263.1), *Sesamum indicum* (XP_011099036.1), *Ipomoea nil* (XP_019195337.1). However, the presence of cysteine heme-iron ligand signature sequence is predicted by PROSITE server which is positioned at 407–416 amino acid sequence. The signature sequence is shaded in yellow.

FIGURE S4 Phylogenetic analysis of *CYP85A69* from *W. somnifera* with *CYP85* genes of other plant species.

FIGURE S5 Analysis of Green Fluorescent protein in infiltered leaves: **(A–I)** Green Fluorescent Protein detection in infiltered leaves of *W. somnifera* performed using fluorescent microscope. *WsCYP85A69* is fused with N-terminal fragment of GFP in pCAMBIA1302. Construct was infiltered into leaves of *W. somnifera*. GFP fluorescence was detected after third day of post-infiltration. Experiment was repeated three times. **(J)** Vector map citing the position of *WsCYP85A69* in pCAMBIA1302.

FIGURE S6 Analysis of promoter region: Isolation of promoter region of *WsCYP85A69* was performed using genome walker kit and analysis was performed using PlantCare and PLACE server tools to reveal the presence of various putative *cis*-acting regulatory elements.

TABLE S1 List of primers used in experiments.

FILE S2 **(a)** HPLC chromatogram of castasterone in different parts of plant. The chromatogram represents chemo-profile of one sample only. The experiment was repeated three times. **(b)** HPLC chromatogram of infiltered leaves with overexpression construct. The chromatogram represents chemo-profile of one sample only. The experiment was repeated three times. **(c)** HPLC chromatogram of infiltered leaves with silencing construct. The chromatogram represents chemo-profile of one sample only. The experiment was repeated three times. **(d)** HPLC chromatogram of leaves treated with methyl jasmonate (MeJA), abscisic acid (ABA) and cold treatment (CT). The chromatogram represents chemo-profile of one sample only. The experiment was repeated three times.

FILE S3 **(a)** HPLC chromatogram of stigmasterol levels in infiltered leaves with overexpression construct. The chromatogram represents chemo-profile of one sample only. The experiment was repeated three times. **(b)** HPLC chromatogram of stigmasterol levels in infiltered leaves with silencing constructs. The chromatogram represents chemo-profile of one sample only. The experiment was repeated three times.

FILE S4 **(a)** HPLC chromatogram of withanolides levels in infiltered leaves with overexpression construct. The chromatogram represents chemo-profile of one sample only. The experiment was repeated three times. **(b)** HPLC chromatogram of withanolides levels in infiltered leaves with silencing constructs. The chromatogram represents chemo-profile of one sample only. The experiment was repeated three times.

FILE S5 Multiple sequence alignment of homologs of *CYP85A1* gene from different plant species used for designing degenerate primers.

## Abbreviations

*A. tumefaciens*: *Agrobacterium tumefaciens* Gv3101 strain
ABA: Abscisic acid
aMIR: artificial micro-RNA mediated silencing
CT: Cold treatment
GFP: Green fluorescent protein
GSPs: Gene specific primers
HPLC: High performance liquid chromatography
LC-PDA-MS: Liquid chromatography equipped with photodiode array and mass spectroscopy analysis
MeJA: Methyl jasmonate
ORF: Open reading frame
qRT-PCR: Quantitative real-time polymerase chain reaction
RACE: Rapid amplification of cDNA ends
*S. cerevisiae*: *Saccharomyces cerevisiae* WAT11 strain.
