# Supplementary material for: Gene Silencing and Over-Expression Studies in Concurrence With Promoter Specific Elicitations Reveal the Central Role of WsCYP85A69 in Biosynthesis of Triterpenoids in Withania somnifera (L.) Dunal
Source: Front Plant Sci. 2019 Jul 5;10:842. doi: 10.3389/fpls.2019.00842 (PMC6624744; doi:10.3389/fpls.2019.00842)
Supplement: FIGURE S1 — Prediction of conserved amino acid residues for WsCYP85A69. The analysis of conserved residues for WsCYP85A69 was done using ConSurf and ConSeq web servers. Conservation scale used for residues analysis, from variable to conserved region, is shown in- blue (1) to purple colors (9). Abbreviations used are: e = An exposed residue according to the neural-network algorithm; b = buried residue according to the neural-network algorithm; f = predicted functional residue (highly conserved and exposed); s = predicted structural residue (highly conserved and buried); and X = insufficient data, the calculation for this site was performed on less than 10% of the sequences. [file Data_Sheet_1.PDF]

Supplementary Figure 1: Prediction of conserved amino acid residues for *WsCYP85A69*: The analysis of conserved residues for *WsCYP85A69* was done using ConSurf and ConSeq web servers. Conservation scale used for residues analysis, from variable to conserved region, is shown in- blue (1) to purple colours (9). Abbreviations used are: e= An exposed residue according to the neural-network algorithm; b= buried residue according to the neural-network algorithm; f= predicted functional residue (highly conserved and exposed); s= predicted structural residue (highly conserved and buried); and X= insufficient data, the calculation for this site was performed on less than 10% of the sequences.

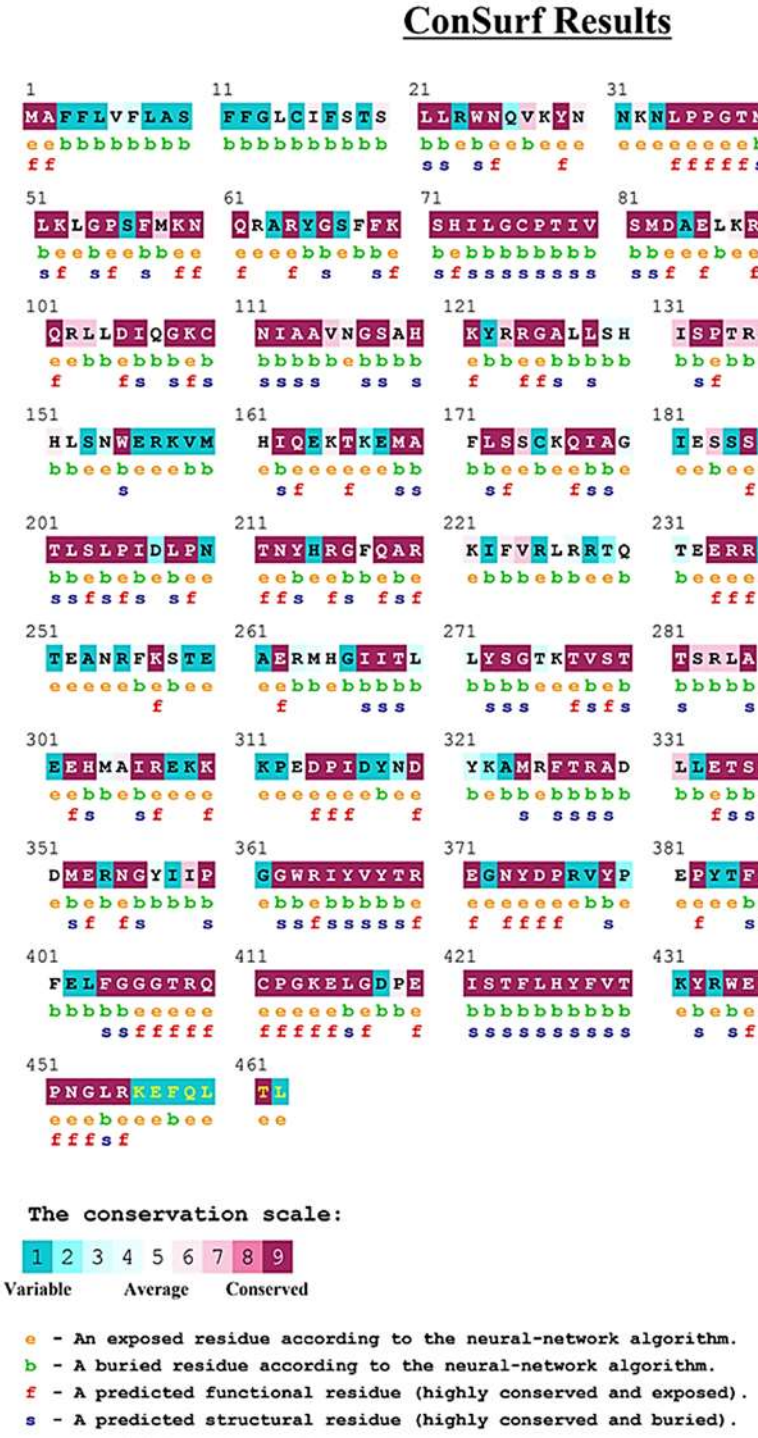

Supplementary Figure 2: Three-dimensional model and ligand-binding site prediction for *WsCYP85A69*: A- Display of ribbon model of three-dimensional structure of *WsCYP85A69* predicted via Phyre2 web server, using crystal structure of lanosterol 14-alpha demethylase as template for modelling by achieving 100% coverage score, **B-** Ligand-binding sites (zoom view) as predicted by GALAXY web server displays the presence of heme-binding site and presence of I^{112}, H^{120}, M^{244}, T^{269}, L^{270}, S^{273}, T^{277}, E^{340}, V^{344}, R^{346}, L^{403}, F^{404}, R^{409}, C^{411}, P^{412}, G^{413}, L^{416}, G^{417} residues in its ligand binding sites.

A

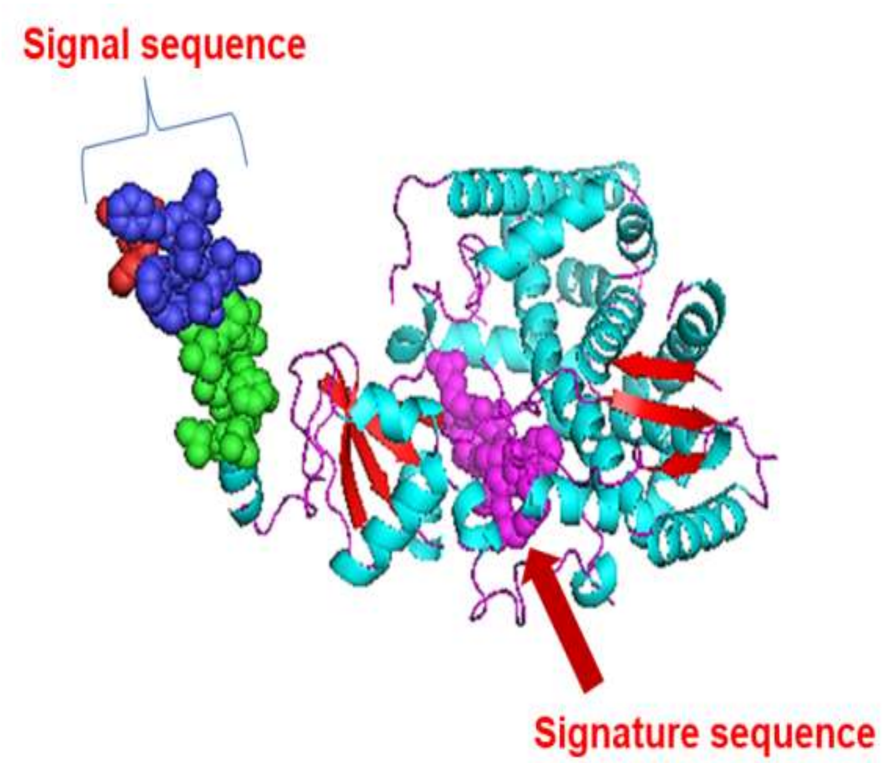

B

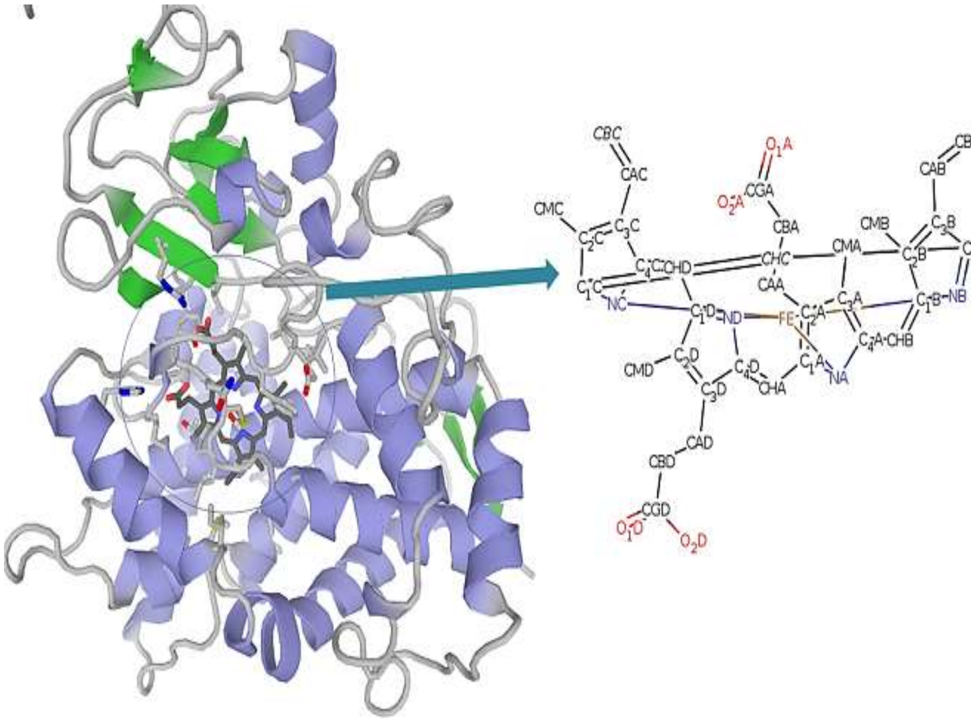

Supplementary Figure 3: Multiple sequence alignment of amino acid sequence of *WsCYP85A69* with its homologs: T-COFFEE web server was used to align the sequences and gave a score of 99. Consensus residues are shaded in maroon colour. Sequences used were from *Withania somnifera* (*WsCYP85A69*), *Capsicum anuum* (PHT91631.1), *Nicotiana tabacum* (NP_001312136.1), *Solanum tuberosum* (NP_001234263.1), *Sesamum indicum* (XP_011099036.1), *Ipomoea nil* (XP_019195337.1). However, the presence of cysteine heme-iron ligand signature sequence is predicted by PROSITE server which is positioned at 407-416 amino acid sequence. The signature sequence is shaded in yellow.

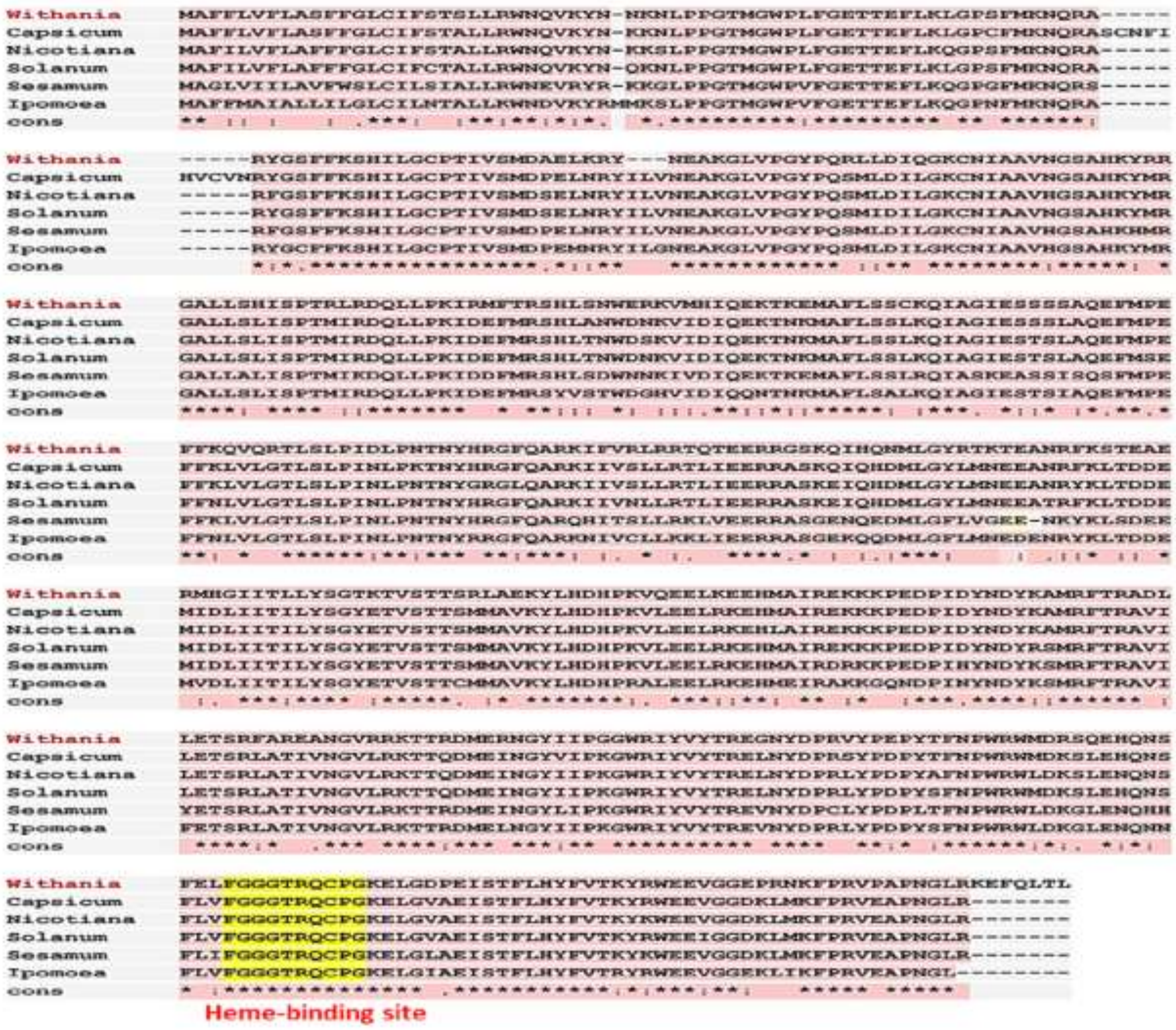

Supplementary Figure 4: Amino acid sequences of *CYP85A69* gene from *Withania somnifera* and its characterized homologs from different plant species used for phylogenetic analysis.

> *WsCYP85A69*

MAFFLVFLAS FFGLCIFSTS LLRWNQGQVE QQKLAPWYYR LATFWGDYEV FFKLGPSFMK
NQRARYGSFF KSHILGCPTN SFNGCRVEKI YISERGKRVG PRDTHRDCLI FKGNAILQQS
MVQLTSNGGV HCYLTSALPD SETNFCPKFG CRRQDPTCPN GERSKVMHIQ EKTKEMAFLS
SCKQIAGIES SSSAQDFMPE FFKQVHRTLS LPIDLPNTNY HRRFQARKIF VRLRRTQTEK
RRGSKQIHQN MLGYRTKTEA NRFKSTEAER MHGIITLLYS GTKTVSTTSR LAEKYLHDHP
KVQEQLKEEH MAIREKKKPE DPIDYNDYKE MRFTRADLLE TSIFAREANG VQRKTTRDME
RNGYIIPGGW RIYVYTREGN YDPRVYPEPY TFNPWRWMDR SQEHQNSFEL FGGGTLQCPG
KELGDPEIST FLHYFVTKYR WEEVGGEPRN KFPRVPAPNG LRKEFQLTLL

>*SlCYP85A1*

MAFFLIFLSSFFGLCIFCTALLRWNQVKYNQKNLPPGTMGWPLFGETTEFLKLGPSFMKNQRARYGSFFK
SHILGCPTIVSMDSELNRYILVNEAKGLVPGYPQSMIDILGKCNIAAVNGSAHKYMRGALLSLISPTMIRD
QLLPKIDEFMRSHLTNWDNKVIDIQEKTNKMAFLSSLKQIAGIESTSLAQEFMSEFFNLVLGTLSLPIN
LPNTNYHRGFQARKIIVNLLRTLIEERRASKEIQHDMLGYLMNEEATRFKLTDDEMIDLIITILYSGYET
VSTTSMMAVKYLHDHPKVLEELRKEHMAIREKKKPEDPIDYNDYRSMRFTRAVILETSRLATIVNGVLR
KTTQDMEINGYIIPKGWRIYVYTRELNYDPRLYPDPYSFNPWRWMDKSLEHQNSFLVFGGGTRQCPGKE
LGVAEISTFLHYFVTKYRWEEIGGDKLMKFPRVEAPNGLRIRVSAH

>SlCYP85A3
MAIFLIIFVVFFGFCILSTPLFRWIDIVYNKKNLPPGTMGWPIFGETREFLNQGPNFMKN
QRARYGNFFKSHILGCPTVVSMDAGLNVYILNNEAKGLIPGYPQSMLDILGKCNIAAVHG
ATHKYIRGALLSLINPTMIKDHILPKIDKFMRSHLSGWDNCNVIDIQQMTKEMAFFSSLD
QIGGFATSSSIAQEFRAGFLNIALGTISLPINFPTTNYYRGLQGRKTIVKLLRKIIEDRR
GSKKIQQDMLGLMMNEEAKNRYTLSDEELIDQIITIMYSGFETVSTTSMMAVKYLHDHPK
ALEEIRKEHFAIREKKSLEDPIDYNDFKAMRFTRAVIYETLRLATIVNGVLRKTTQDMEL
NGYMIPKGWRIYVYTRELNYDPLIYPDPYTFNPWRWLENNLDHQSSFLMFGGGTRLCPGK
ELGVAEISTFLHYFVTRYRWEEVGGNKLMKFPRVEALNGLWIKVSAY
>SoCYP85A
MAVFMVVFAVIFSLFCFSSALLRWNELRYRKKGLPPGTMGWPIFGETTEFLKQGPNFIKNQRSRYGNFFK
SHILGCPTIVSMDAELNRFILMNESKGLVPGYPQSMLDILGKCNIAAVHGSTHKYMRGALLSLVSPTMIRD
HILPKVDQFMRSHLSNWQNHVIDIQQKTKEMAFLSSLKQIAGIESSSTAQLFMSEFFKLVEGTLSLPIDLPG
TNYRRGFQARKVIVNILTQLIKERRASKTKDVDILNCLLKEEENKYKLSDEEIIDLIITLAYSGYET
VSTTSMMAVKYLHDHPHVLEELRKEHLAIRAKKKPEDPINWEDYKAMKFTRAVIFETSRLATIVNGVLR
KTTKEMEINGFVIPEGWRIYVYTREVNYDPYLYPDPLVFNPWRWLDRSLESKNYFLIFGGGTRQCPGKEL
GIAEISTFLHYFVTRYRWEEEEGNKLVKFPRVEAPNGLRIRVSSY
>*AtCYP85A2*
MGIMMMILGLLVIIVCLCTALLRWNQMRYSKKGLPPGTMGWPIFGETTEFLKQGPDFMKNQRLRYGSFF
KSHILGCPTIVSMDAELNRYILMNESKGLVAGYPQSMLDILGTCNIAAVHGPSHRLMRGSLLSLISPTMM
KDHLLPKIDDFMRNYLCGWDDLETVDIQEKTKHMAFLSSLLQIAETLKKPEVEEYRTEFFKLVVGTLSVP
IDIPGTNYRSGVQARNNIDRLLTELMQERKESGETFTDMLGYLMKKEDNRYLLTDKEIRDQVVTILYSGY
ETVSTTSMMALKYLHDHPKALEELRREHLAIRERKRPDEPLTLDDIKSMKFTRAVIFETSRLATIVNGVLR
KTTHDLELNGYLIPKGWRIYVYTREINYDTSLYEDPMIFNPWRWMEKSLE
SKSYFLLFGGGVRLCPGKELGISEVSSFLHYFVTKYRWEENGEDKLMVFPRVSAPKGYHLKCSPY
>*AtCYP85A1*
MGAMMVMMGLLLIIVSLCSALLRWNQMRYTKNGLPPGTMGWPIFGETTEFLKQGPNFMRNQRLRYGSF
FKSHLLGCPTLISMDSEVNRYILKNESKGLVPGYPQSMLDILGTCNMAAVHGSSHRLMRGSLLSLISSTM
MRDHILPKVDHFMRSYLDQWNELEVIDIQDKTKHMAFLSSLTQIAGNLRKPFVEEFKTAFFKLVVGTLSV
PIDLPGTNYRCGIQARNNIDRLLRELMQERRDSGETFTDMLGYLMKKEGNRYPLTDEEIRDQVVTILYSG
YETVSTTSMMALKYLHDHPKALQELRAEHLAFRERKRQDEPLGLEDVKSMKFTRAVIYETSRLATIVNG
VLRKTTRDLEINGYLIPKGWRIYVYTREINYDANLYEDPLIFNPWRWMKKSLESQNSCFVFGGGTRLCPG
KELGIVEISSFLHYFVTRYRWEEIGGDELMVFPRVFAPKGFHLRISPY
>OsCYP85A1
MVLVAIGVVVAAAVVVSSLLLRWNEVRYSRKRGLPPGTMGWPLFGETTEFLKQGPSFMKARRLRYGSV
FRTHILGCPTVVCMEAELNRRALASEGRGFVPGYPQSMLDILGRNNIAAVQGPLHRAMRGAMLSLVRPA
MIRSSLLPKIDAFMRSHLAAWSSSSSSAVVDIQAKTKEMALLSALRQIAGVSAGPLSDALKAELYTLVLG
TISLPINLPGTNYYQGFKARKKLVAMLEQMIAERRSSGQVHDDMLDALLTGVEGTREKLTDEQIIDLIITLI
YSGYETMSTTSMMAVKYLSDHPKALEQLRKEHFDIRKGKAPEDAIDWNDFKSMTFTRAVIFETLRLATV
VNGLLRKTTQDVEMNGYVIPKGWRIYGYTREINYDPFLYPDPMTFNPWRWLEKNMESHPHFMLFGGGS
RMCPGKEVGTVEIATFLHYFVTQYRWEEEGNNTILKFPRVEAPNGLHIRVQDY
>PsCYP85A1
MVFFMVIFGV FFILCLCSAL LRWNEVRYRK KGLPPGTMGW PVFGETTEFL KQGPNFMKNQ
RLRFGSFFKS HILGCPTIVS MDAEVNRYIL MNESKGLVPG YPQSMLDILG KCNIAAVHGS
THKYLRGALL SIISPTMIRD QILPKIDEFM SFQLSHWDDK IINIQEKTKE MVFLSSLKQI
ASMDSTSKTA DSFKTEFFKL VLGTISLPIN LPGTNYHRGF QARKNIVNIL RELLKERRAS
NETHKDILGC LMENEENKYK LSDEEIIDLV ITLMYSGYET VSTTSMMAVK YLHDHPKALE
EIRKEHLAIR ERKKPNEPID FNDIKSMRFT RAVIFETSRL ATIVNGVLRK TTQDMELNGY
LIPKGWRIYV YTREINYDPF LYPEPLAFNP WRWMDKSLES SNYFLIFGGG TRLCPGKEAG
ITEISTFLHY LLTRYRWEET GGDKLMKFPR VQAPNGLHMK FSSFNN
>PsCYP85A6
MAIFIAILAFFFVFCFFSALLKWNEVRYRRKGLPQGTMGWPVFGETTEFLKQGPNFMINQ
RSRYGNIFKSHILGCPTIVSMDPELNRYILMNEAKGFVPGYPQSMLDILGKCNIAAVHGS
THKYMRGTLLSIISPTLIRNQLLPKIDQFMRTHLSHWENKVINIQDKTKQMAFLSSLKQI
AGMETSSISQPFMTEFFKLVLGTLSLPLNLPGTNYRRGLQARKSIISILSKLLKERRESK
EKYEDMLSCLMRGNNDNNRCKLNDEELIDLIITIMYSGYETISTTSMMAVKYLHDHPKVL
EEMRKEHFAIRERKKPEDPIDCNDLKSMRFTRAVIFETSRLATIVNGVLRKTTHDMELNG
YLVPKGWRIYVYTREINYDPFLYHDPLTFNPWRWLGNSLESQSHFLIFGGGTRQCPGKEL
GIAEISTFLHYFVTRYRWEEVGGDKLMKFPRVVAPNGLHIRVSSY
>VvCYP85A1
MAVFGVVLIGLCICTALLRWNEVRYRKKGLPPGTMGWPVFGETTEFLKQGPSFMKNQRAR
YGKFFKSHLLGCPTTVSMDPELNRYILMNEAKGLVPGYPQSMLDILGKCNIAAVHGSTHK
YMRGALLALISPTMIRGQLLPKIDEFMRSHLNKWDTKIINIQEKTKEMALLSSLKQIAGI

ESGTISKEFMPEFFKLVLGTISLPIDLPGTNYRRGFQARKNIVGMLRQLIEERKASQETH
NDMLGCLMRTNENRYKLSDEEIIDLIITILYSGYETVSTTSMMAVKYLHDHPRVLDELRK
EHLAIRERKRPEDPIDWNDYKLMRFTRAVIFETSRLATIVNGVLRKTTKDMELNGFVIPK
GWRIYVYTREINYDPLLYPDPLAFNPWRWLDKSLESQNYFLLFGGGTRQCPGKELGIAEI
STFLHYFVTRYRWEEVGGDKLMKFPRVEAPNGLHIRVSAY

Supplementary Figure 5: Analysis of Green Fluorescent protein in infiltered leaves: (A- I) Green Fluorescent Protein detection in infiltered leaves of *W. somnifera* performed using fluorescent microscope. *WsCYP85A69* is fused with N-terminal fragment of GFP in pCAMBIA1302. Construct was infiltrated into leaves of *W. somnifera*. GFP fluorescence was detected 3 d post infiltration. Experiment was repeated three times. (J) Vector map citing the position of *WsCYP85A69* in pCAMBIA1302

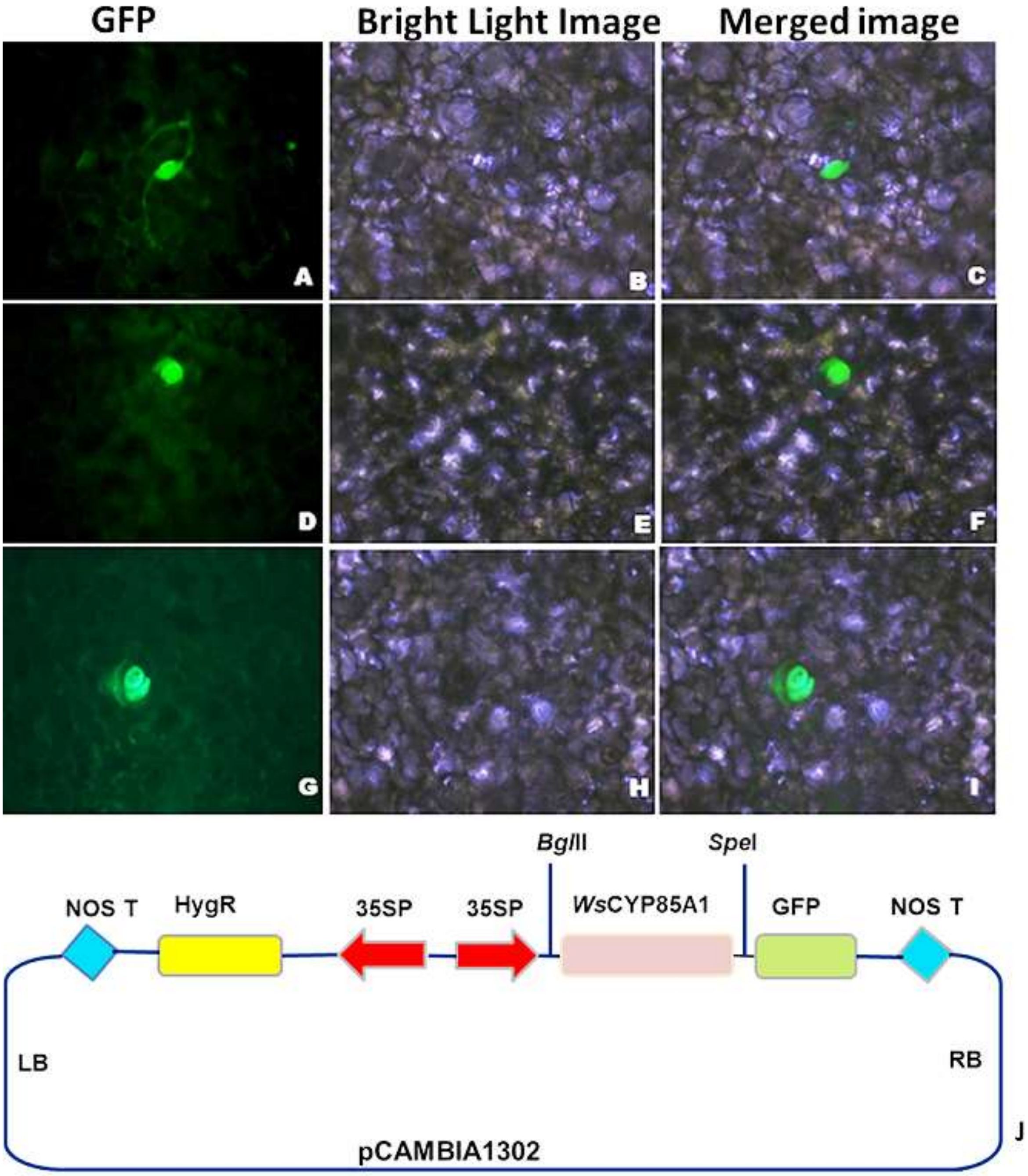

Supplementary Figure 6: Analysis of promoter region: Isolation of promoter region of *WsCYP85A69* was performed using genome walker kit and analysis was performed using PlantCare and PLACE server tools to reveal the presence of various putative *cis*-acting regulatory elements

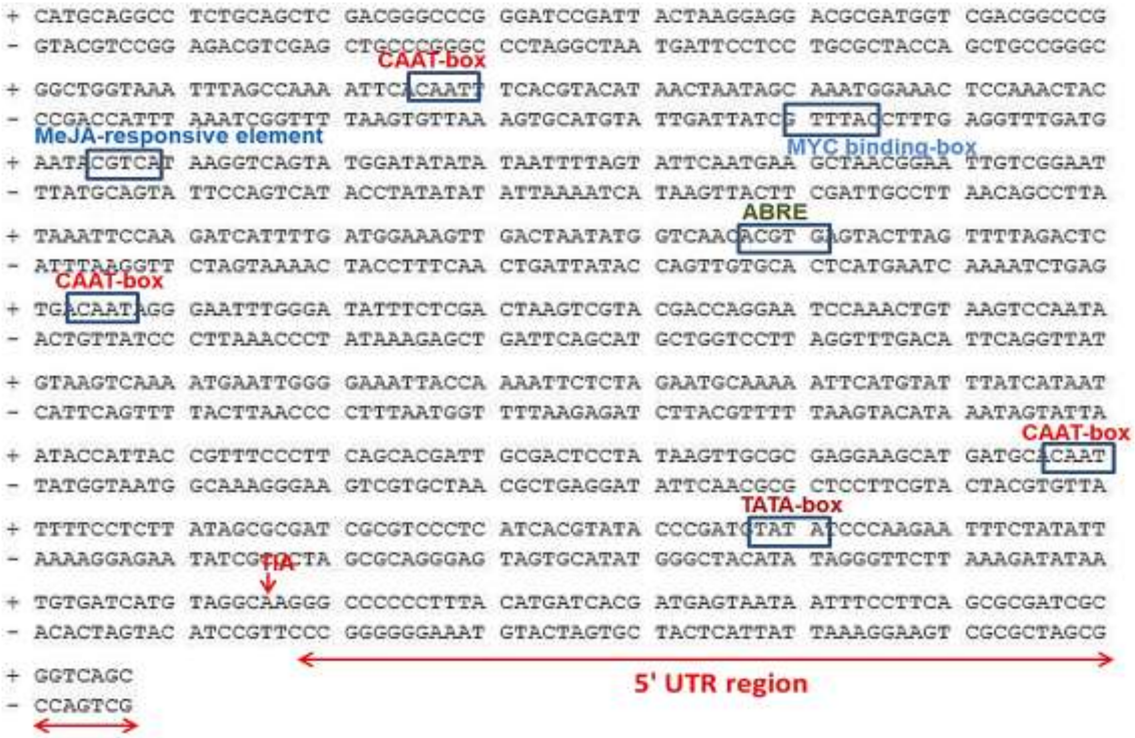

Supplementary Table 1: Primers used in the experiment.

S.No.	Primer´s Name	Primer´s Sequences	Application
**1.**	**CYP85 deg Fwd**	5´GGGAAATGCAATATTGCAGCAGTC 3´	Core amplification
**2.**	**CYP85 deg Rev**	5´GTTTTTCGTCGAACTCCATTCGC 3´	Core amplification
**3.**	**CYP85 5´ OUT**	5´GTTGGACACCCAAAAAATGTGTG 3´	RACE amplification
**4.**	**CYP85 5´ INN**	5´CCAACCCTTTTGCCTCGTTCAC 3´	RACE amplification
**5.**	**CYP85 3´ OUT**	5´GGAATTCTTCAAGCTAGTACTAGG 3´	RACE amplification
**6.**	**CYP85 3´ INN**	5´GCTGGGATTGAATCTAGCTCTTTAGC 3´	RACE amplification
**7.**	**CYP85 FL Fwd**	5´ATGGCTTTCTTCTTAGTTTTTCTTGCTTCC 3´	Full length cloning
**8.**	**CYP85 FL Rev**	5´TTATAATAGAGTGAGTTGAAACTCCTTCCG3´	Full length cloning
**9.**	**CYP85 Pro OUT**	5´TGGCCAACCCATAGTACCAGGGGGCAA 3´	Promoter analysis
**10.**	**CYP85 Pro INN**	5´AGAAGTACTAAAGATGCACAGCCC 3´	Promoter analysis
**11.**	**Walker AP1***	5´GTAATACGACTCACTATAGGGC 3´	Promoter analysis
**12.**	**Walker AP2***	5´ACTATAGGGCACGCGTGGT 3´	Promoter analysis
**13.**	**CYP85 RT Fwd**	5’TTGGTCCCAGGATACCCACAGAGATT 3’	Real-time analysis
**14.**	**CYP85 RT Rev**	5’ TTCTTGGATGTGCATGACTTTGCGTTC 3’	Real-time analysis
**15.**	**Actin RT F**	5´ATGACATGGAGAAGATCTGGCATCA 3´	Real-time analysis
**16.**	**Actin RT R**	5´AGCCTGGATGGCAACATACATAGC 3´	Real-time analysis
**17.**	**CYP85 Exp F**	5´*ACCGGATCC*ATGGCTTTCTTCTTAGTTTTTCTTGC 3’	Gene expression
**18.**	**CYP85 Exp R**	5´*AAGGGTACC*TTATAATAGAGTGAGTTGAAACTCCTT3´	Gene expression
**19.**	**CYP85 BglII F**	5´ *ATAGATCTA*ATGGCTTTCTTCTTAGTTTTTCTTG 3´	Transient overexpression
**20.**	**CYP85 SpeI R**	5´*ATAACTAGT*TAATAGAGTGAGTTGAAACTCCTT 3´	Transient overexpression
**21.**	**CYP85mi1F**	5´AAGATAGATCTAGATCTGACGATGGAAGTAGCGAAATGCAA TATTGCAACATGAGTTGAGCAGGGTA 3´	Artificial micro-RNA mediated silencing
**22.**	**CYP85mi1R**	5´AAAATAGAGCTCGTGAAAGAAGTAGCGAAATGCAATATTGC AAAAAGAAGAGTAAAAGCCATTA 3´	Artificial micro-RNA mediated silencing
**23.**	**CYP85mi2F**	5´AAGATAGATCTAGATCTGACGATGGAAGAAGGTAGTACTAG GCACTCTACATGAGTTGAGCAGGGTA 3´	Artificial micro-RNA mediated silencing
**24.**	**CYP85mi2R**	5´AAAATAGAGCTCGTGAAAGAAGAAGGTAGTACTAGGCACTC TAAAAGAAGAGTAAAAGCCATTA 3´	Artificial micro-RNA mediated silencing

***Primers provided with the kit # Start/stop codon’s are in bold and enzyme sites are italicised**
