# Supplementary material for: Gene Silencing and Over-Expression Studies in Concurrence With Promoter Specific Elicitations Reveal the Central Role of WsCYP85A69 in Biosynthesis of Triterpenoids in Withania somnifera (L.) Dunal
Source: Front Plant Sci. 2019 Jul 5;10:842. doi: 10.3389/fpls.2019.00842 (PMC6624744; doi:10.3389/fpls.2019.00842)
Supplement: FILE S2 [file Data_Sheet_2.PDF]

a) **HPLC chromatograms of different parts of *Withania somnifera***

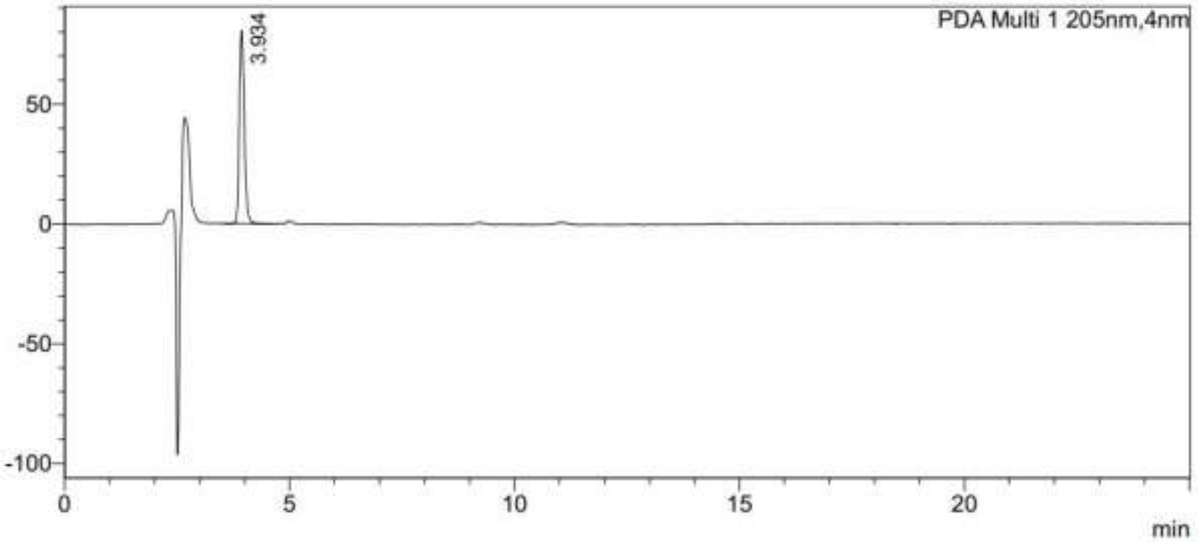

Figure 1: HPLC chromatogram of marker (castasterone)

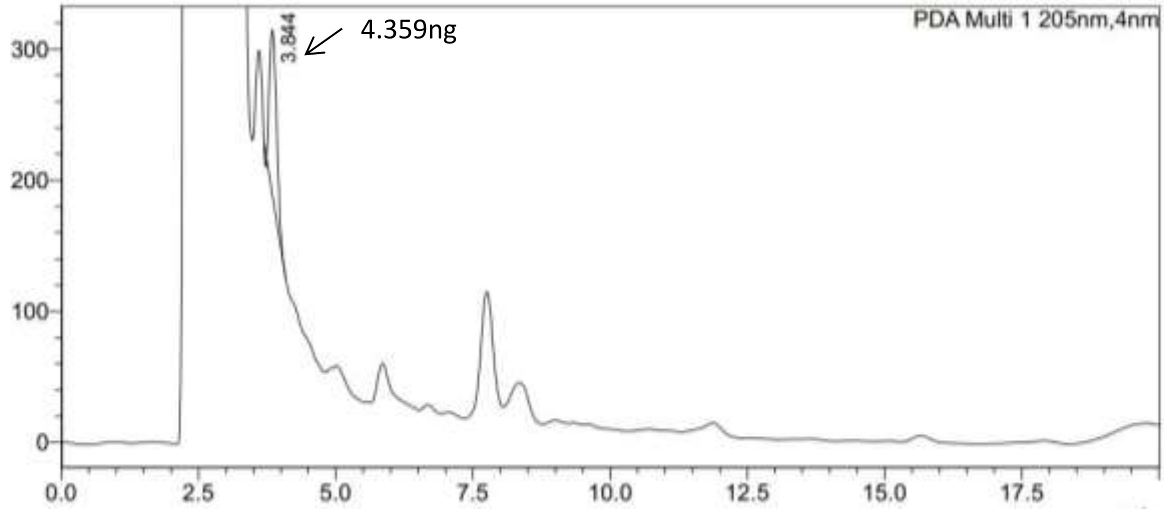

Figure 2: HPLC chromatogram of leaves of *W. somnifera.* The chromatogram represents chemo-profile of one sample only. The experiment was repeated three times.

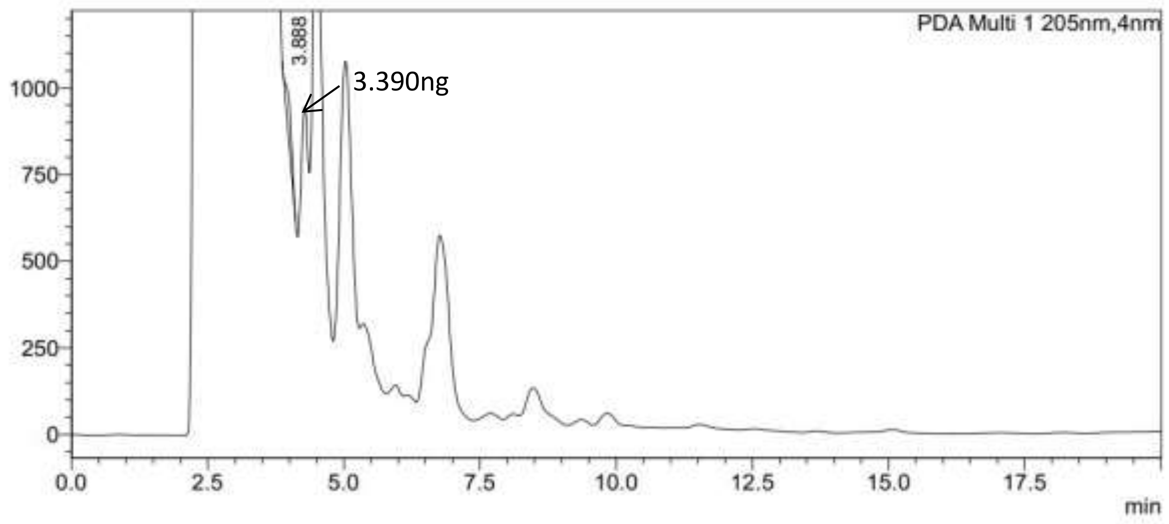

Figure 3: HPLC chromatogram of stem of *W. somnifera.* The chromatogram represents chemo-profile of one sample only. The experiment was repeated three times.

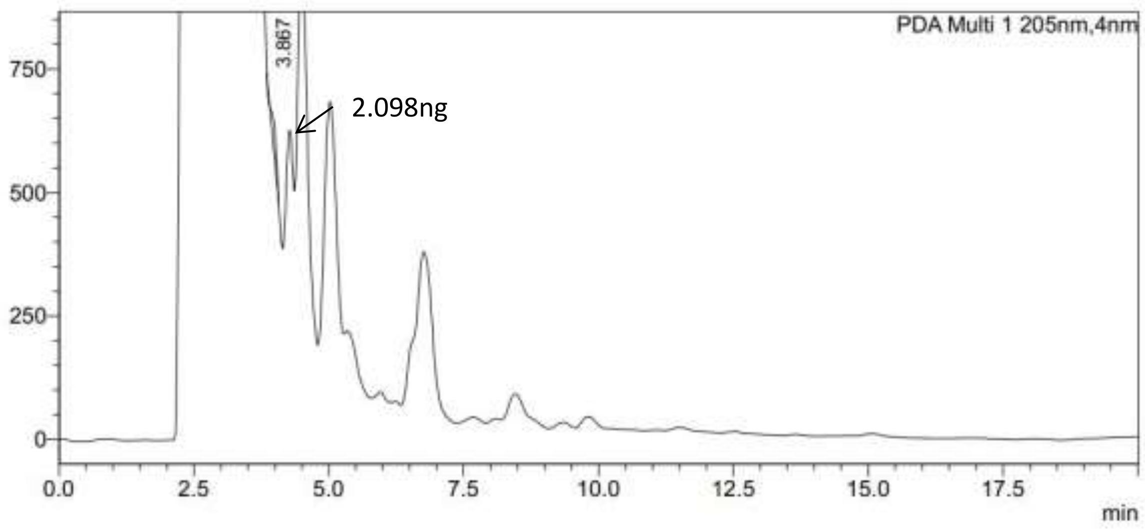

Figure 4: HPLC chromatogram of roots of *W. somnifera.* The chromatogram represents chemo-profile of one sample only. The experiment was repeated three times.

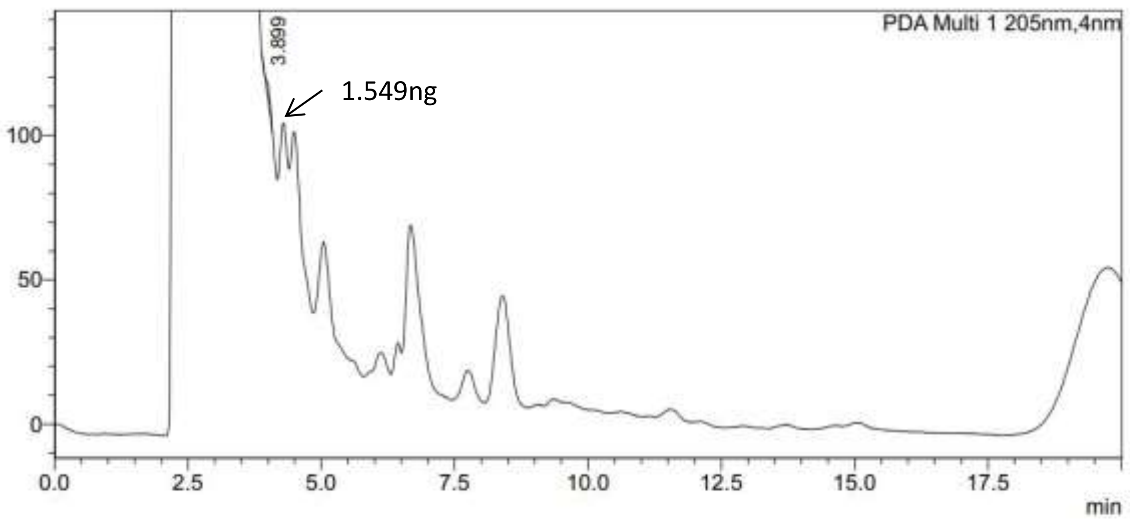

Figure 5: HPLC chromatogram of inflorescence of *W. somnifera*. The chromatogram represents chemo-profile of one sample only. The experiment was repeated three times.

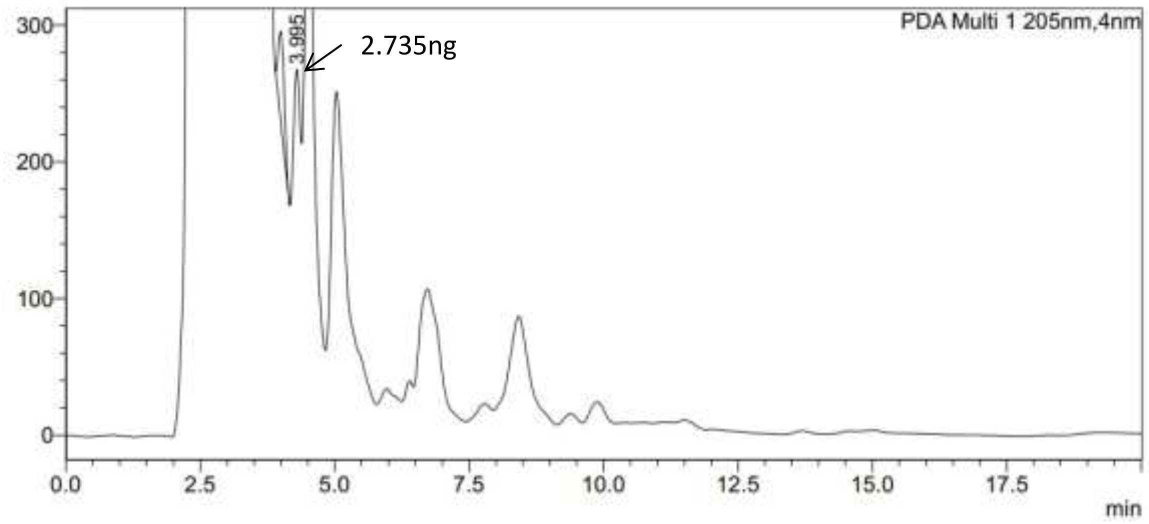

Figure 6: HPLC chromatogram of berries of *W. somnifera*. The chromatogram represents chemo-profile of one sample only. The experiment was repeated three times.

b) Chromatograms of leaves infiltered with over-expression construct

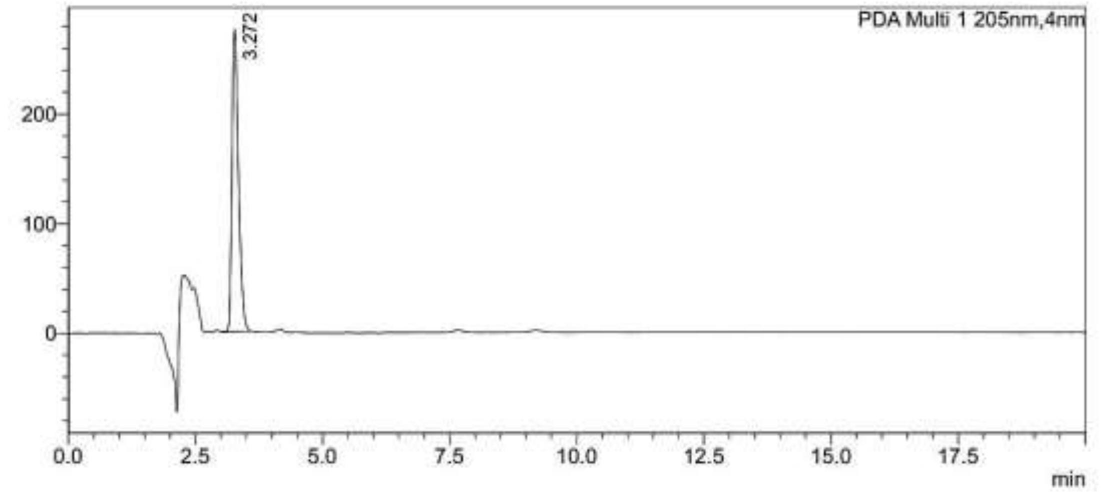

Figure 1: HPLC chromatogram of marker (castasterone)

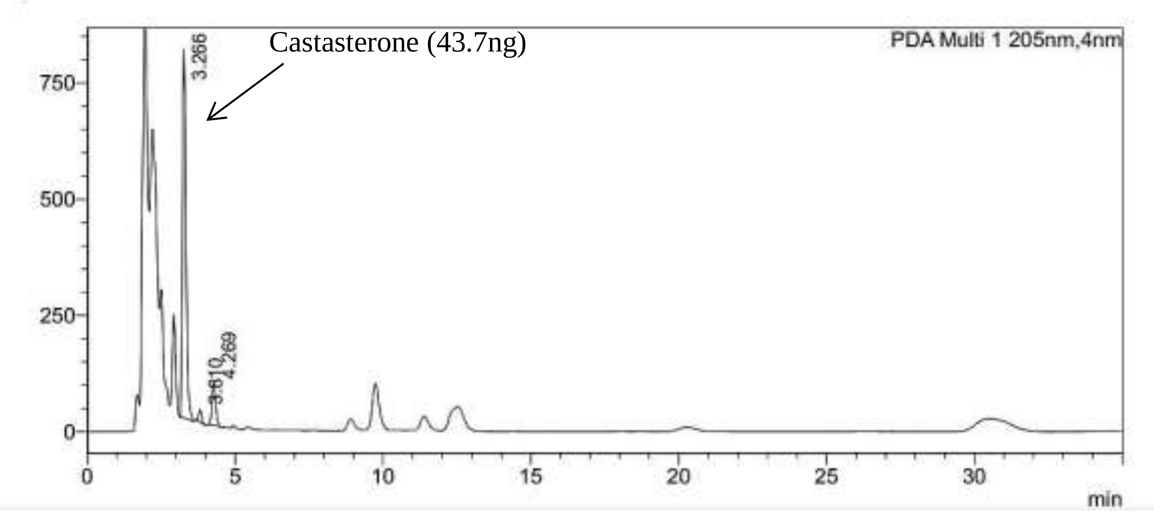

Figure 2: HPLC chromatogram of leaves infiltered with *WsCYP85A69*-pCAMBIA1302 construct, displayed higher accumulation of castatsterone. The chromatogram represents chemo-profile of one sample only. The experiment was repeated three times.

c) Chromatograms of leaves infiltered with silencing constructs

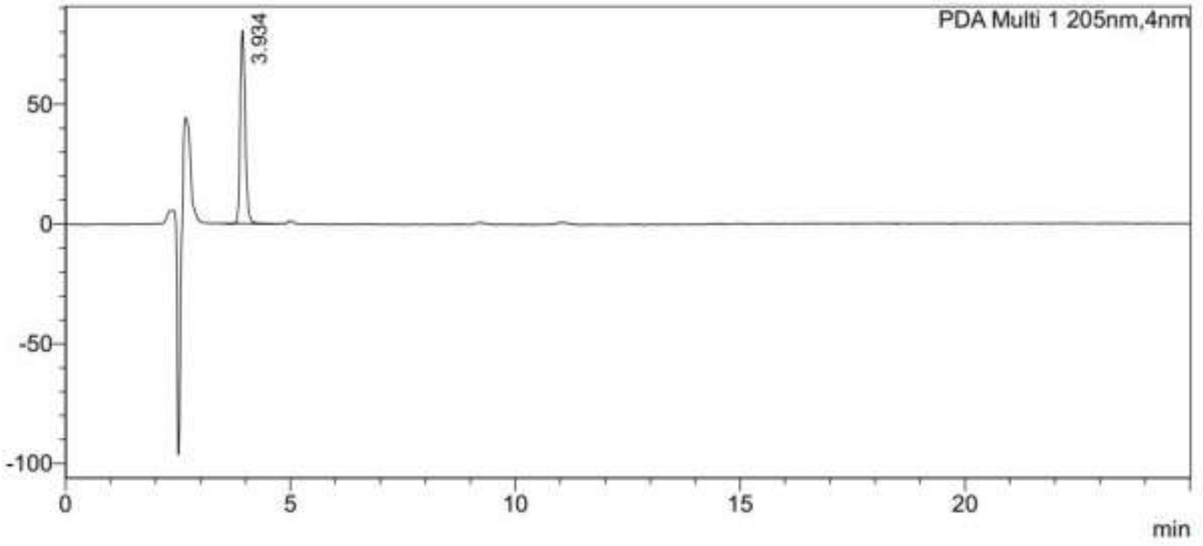

Figure 1: HPLC chromatogram of marker (castasterone)

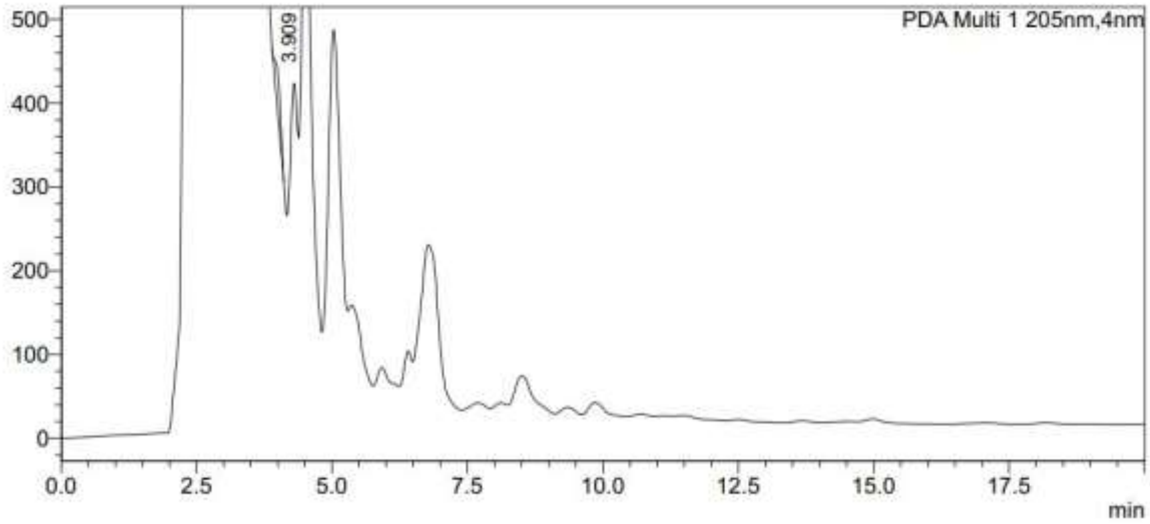

Figure 2: HPLC chromatogram of leaves infiltered with pBI121 (empty vector).

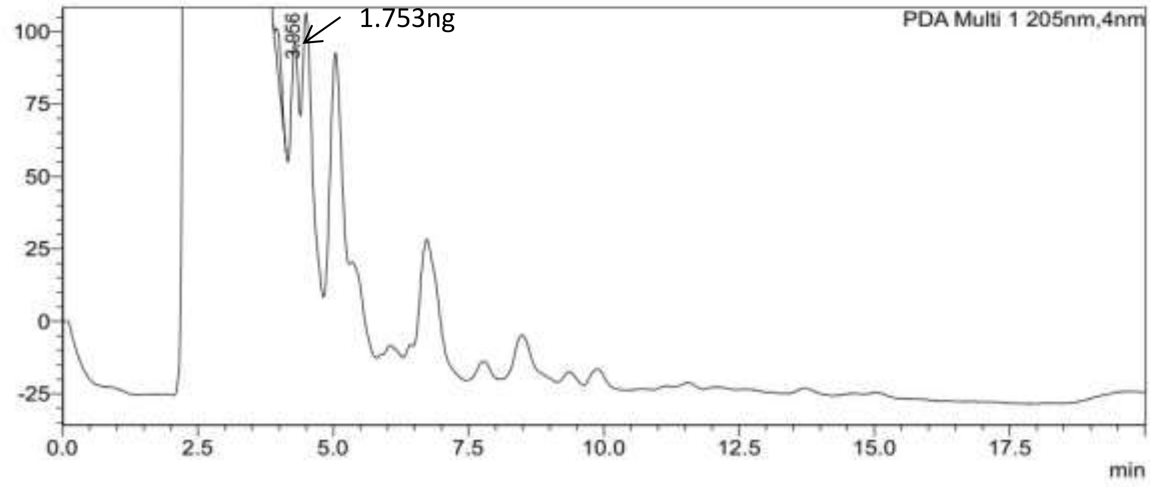

Figure 3: HPLC chromatogram of leaves infiltered with CYP85miRNA1-pBI121. The chromatogram represents chemo-profile of one sample only. The experiment was repeated three times.

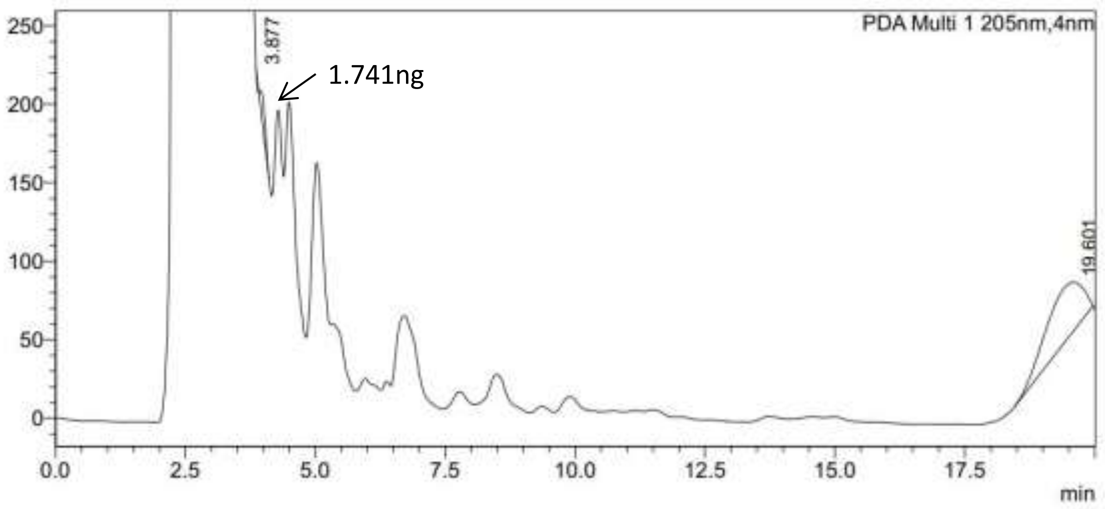

Figure 4: HPLC chromatogram of leaves infiltered with CYP85miRNA2-pBI121. The chromatogram represents chemo-profile of one sample only. The experiment was repeated three times.

d) Chromatograms of leaves treated with methyl jasmonate (MeJA), abscisic acid (ABA) and cold treatment

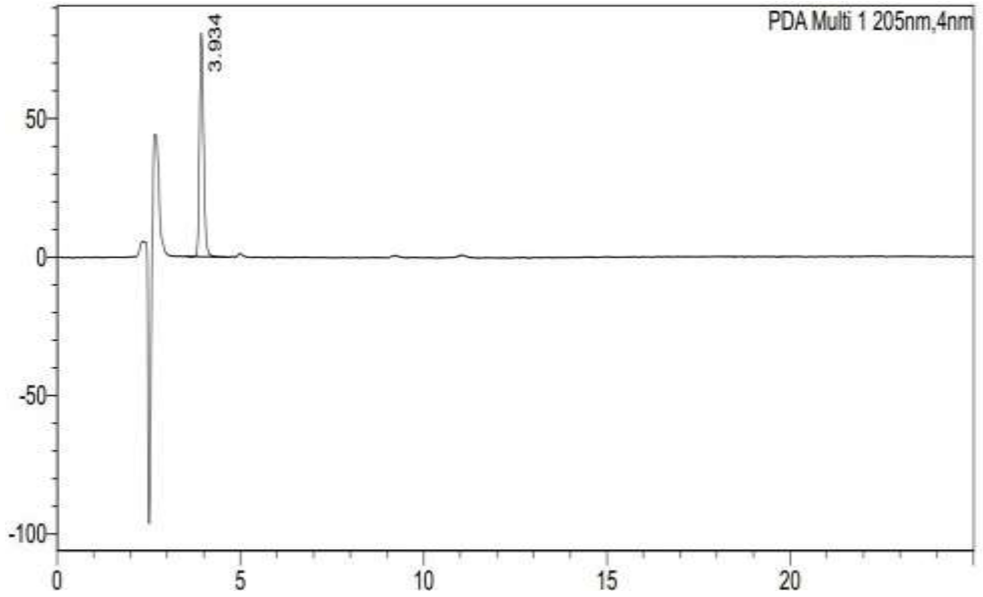

Figure 1: HPLC chromatogram of marker (castasterone)

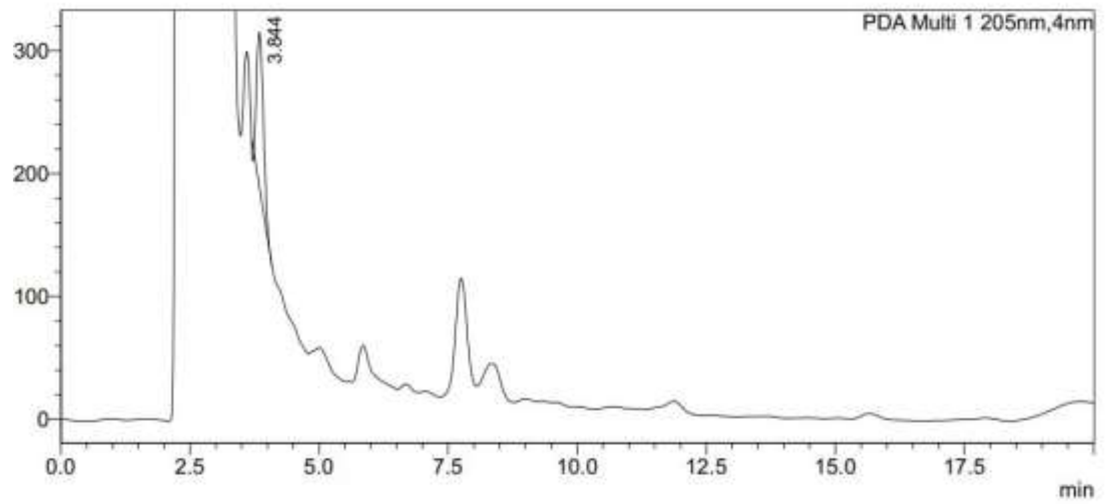

Figure 2: HPLC chromatogram of leaves treated with equal amount of ethanol. The chromatogram represents chemo-profile of one sample only. The experiment was repeated three times.

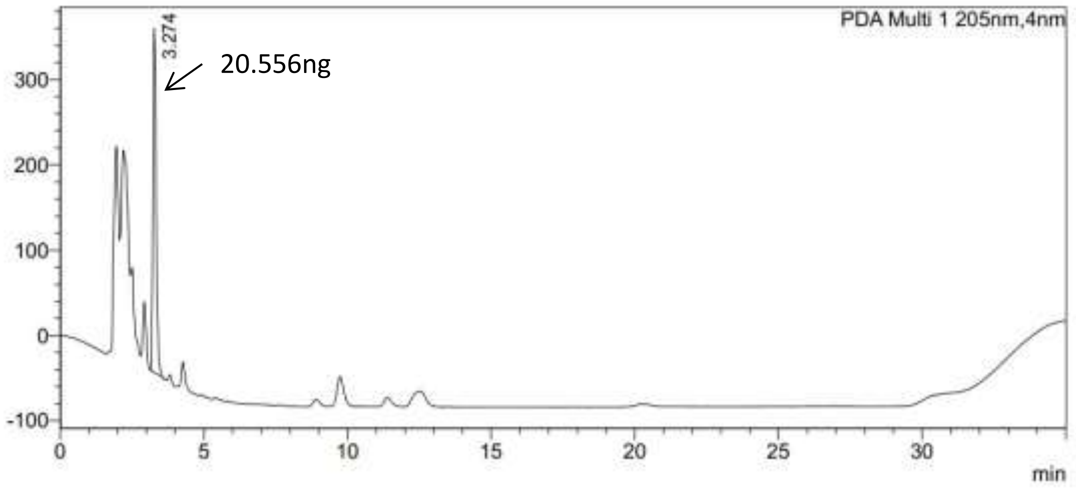

Figure 3: HPLC chromatogram of leaves treated with MeJA. Samples were harvested after 3 h of post treatment. The chromatogram represents chemo-profile of one sample only. The experiment was repeated three times.

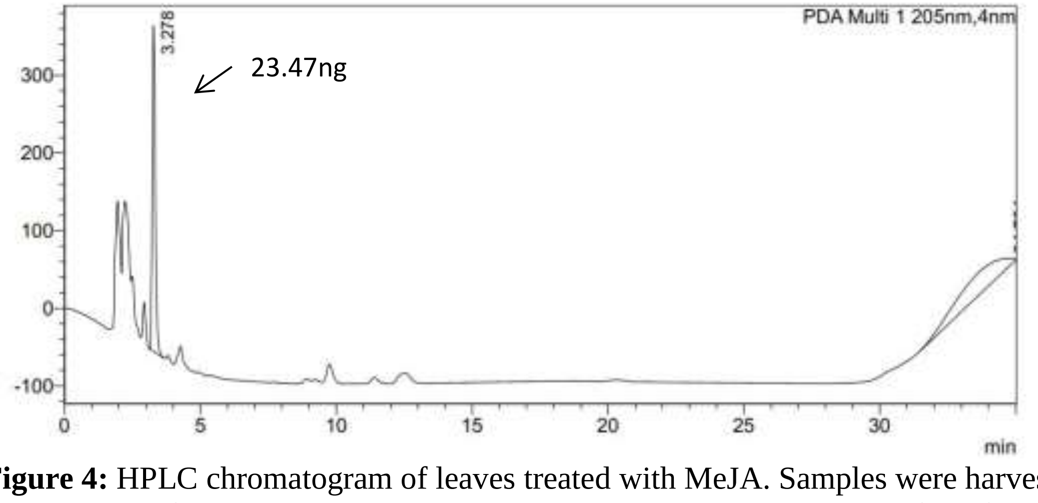

Figure 4: HPLC chromatogram of leaves treated with MeJA. Samples were harvested after 6 h of post treatment. The chromatogram represents chemo-profile of one sample only. The experiment was repeated three times.

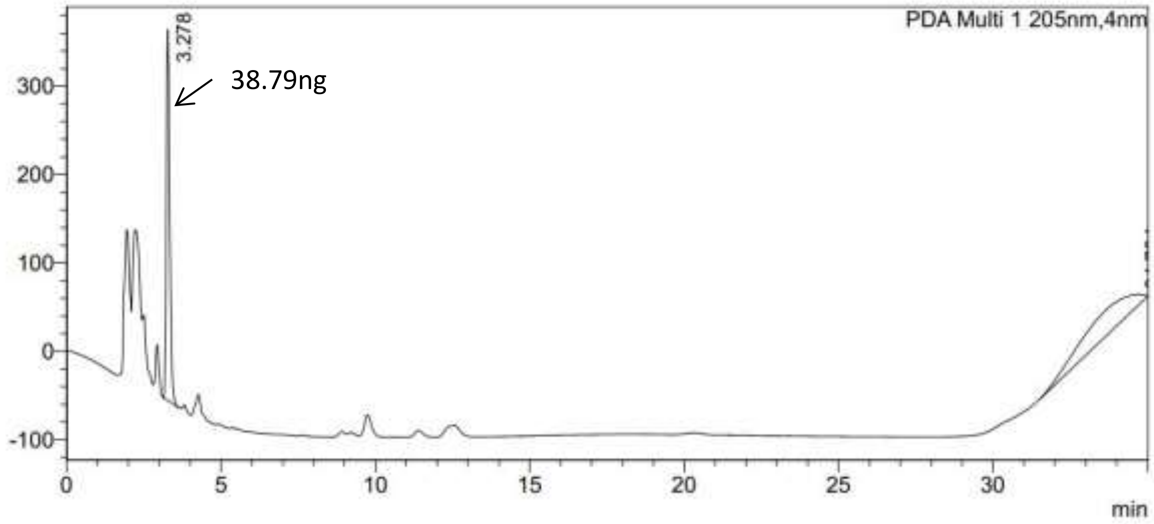

Figure 5: HPLC chromatogram of leaves treated with MeJA. Samples were harvested 12 h of post treatment. The chromatogram represents chemo-profile of one sample only. The experiment was repeated three times.

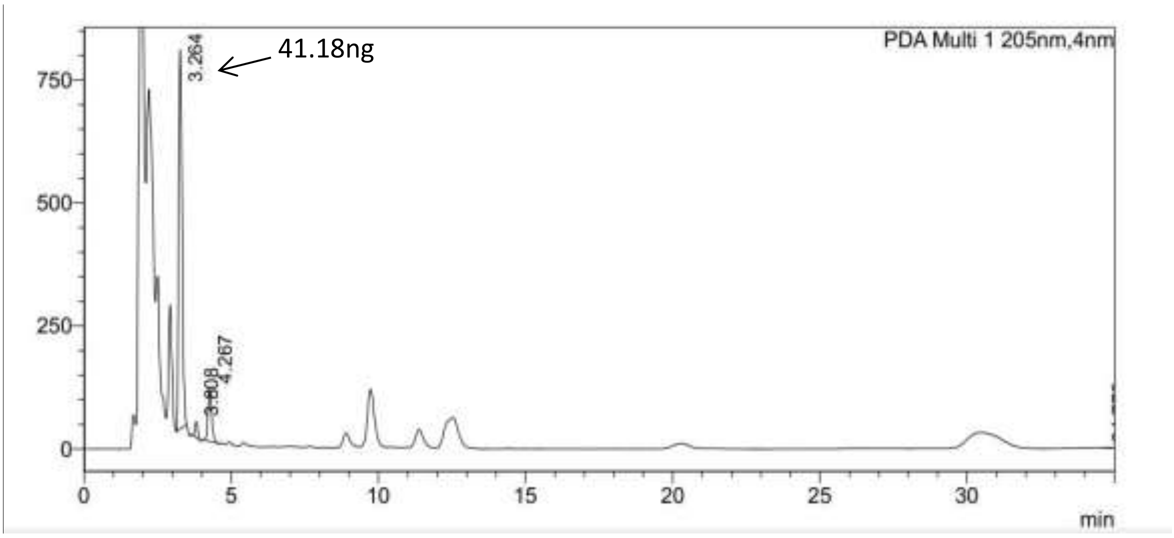

Figure 6: HPLC chromatogram of leaves treated with MeJA. Samples were harvested after 24 h of post treatment. The chromatogram represents chemo-profile of one sample only. The experiment was repeated three times.

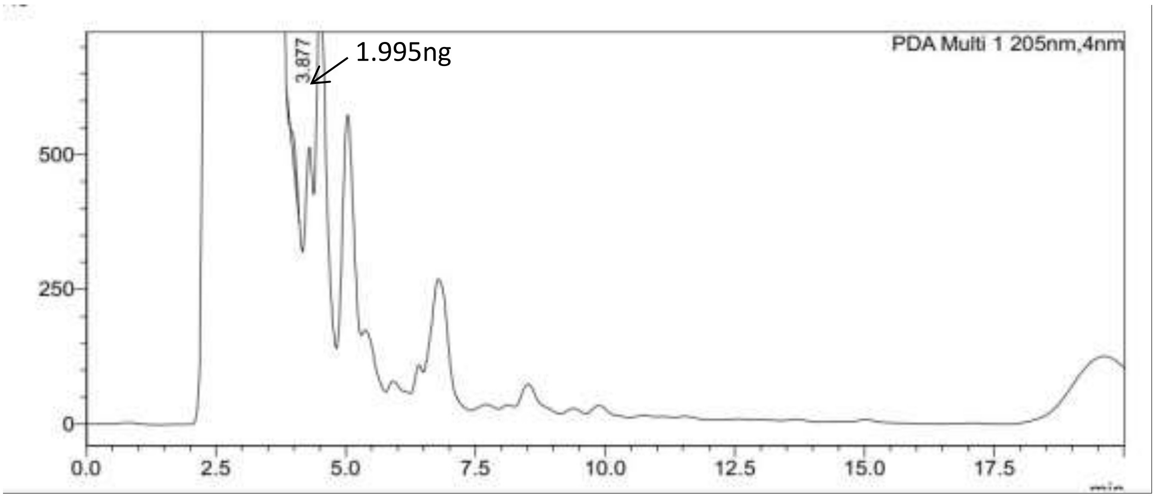

Figure 7: HPLC chromatogram of leaves treated with ABA. Samples were harvested after 3 h of post treatment. The chromatogram represents chemo-profile of one sample only. The experiment was repeated three times.

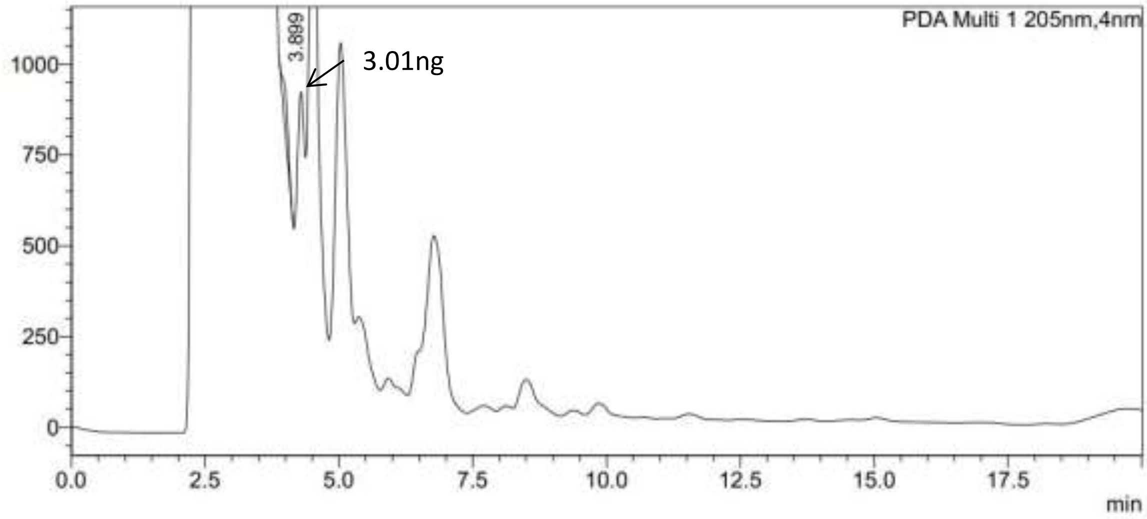

Figure 8: HPLC chromatogram of leaves treated with ABA. Samples were harvested after 6 h of post treatment. The chromatogram represents chemo-profile of one sample only. The experiment was repeated three times.

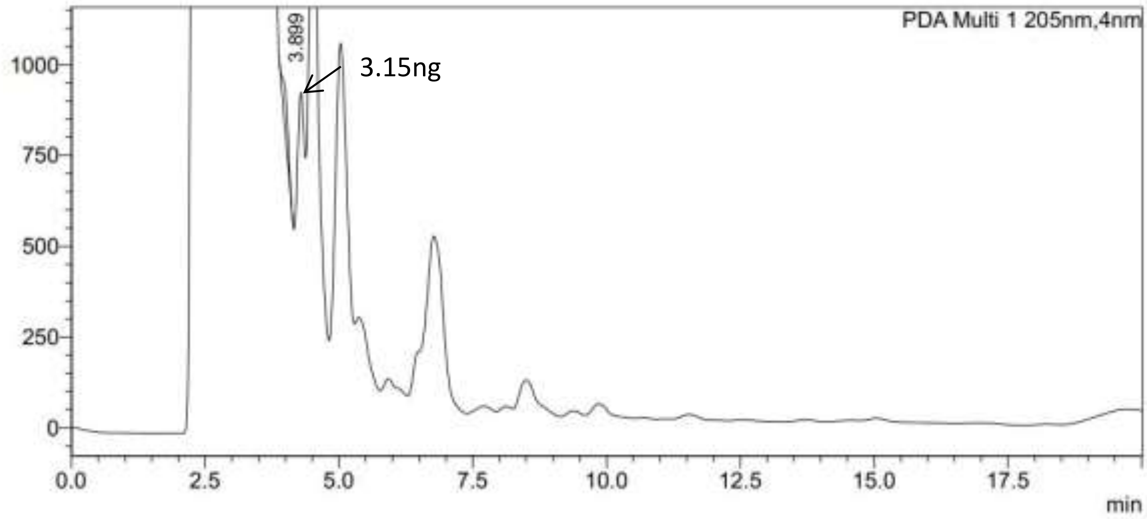

Figure 9: HPLC chromatogram of leaves treated with ABA. Samples were harvested after 12 h of post treatment. The chromatogram represents chemo-profile of one sample only. The experiment was repeated three times.

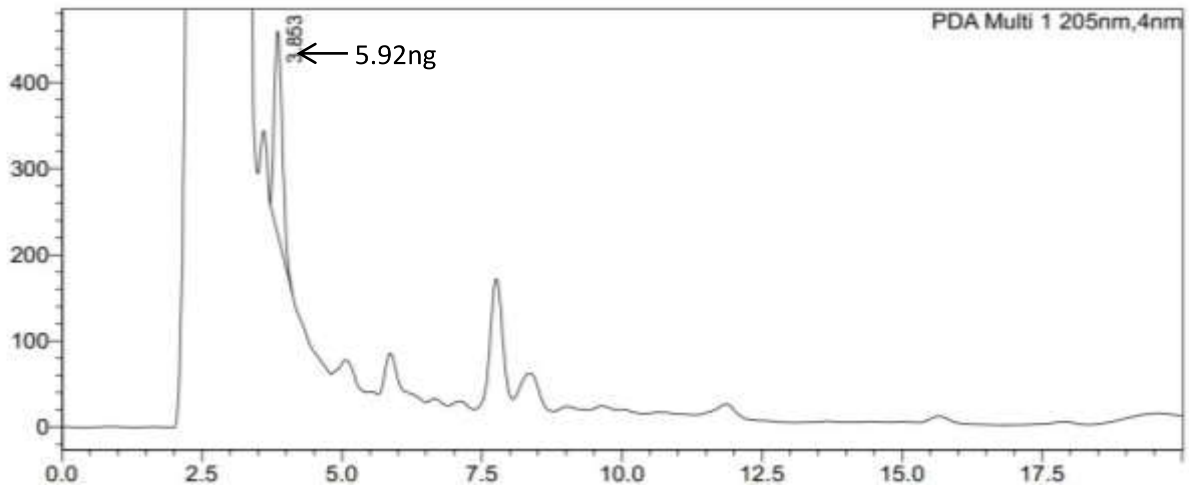

Figure 10: HPLC chromatogram of leaves treated with ABA. Samples were harvested after 24 h of post treatment. The chromatogram represents chemo-profile of one sample only. The experiment was repeated three times.

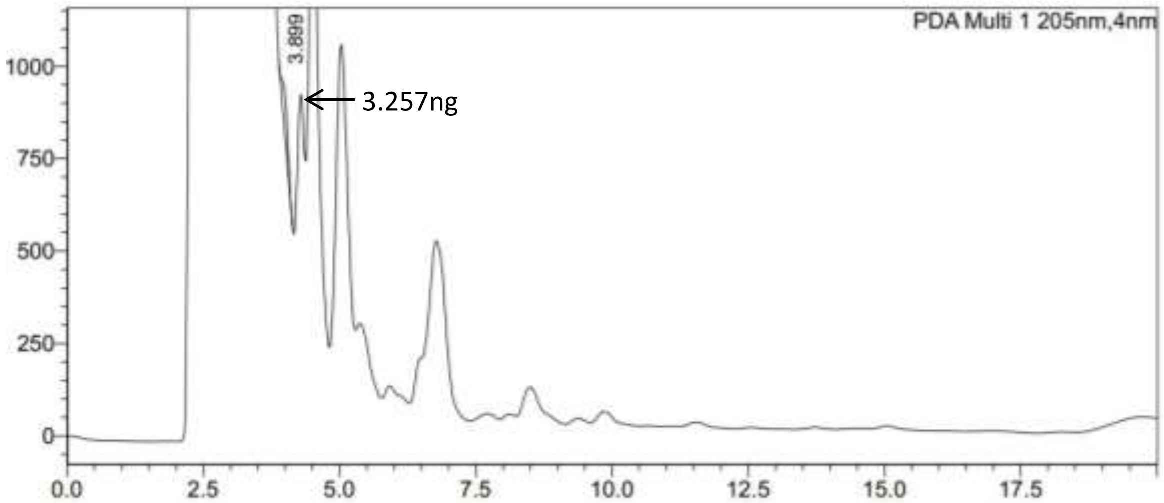

Figure 11: HPLC chromatogram of cold treated plants. Samples were harvested after 3 h of post treatment. The chromatogram represents chemo-profile of one sample only. The experiment was repeated three times.

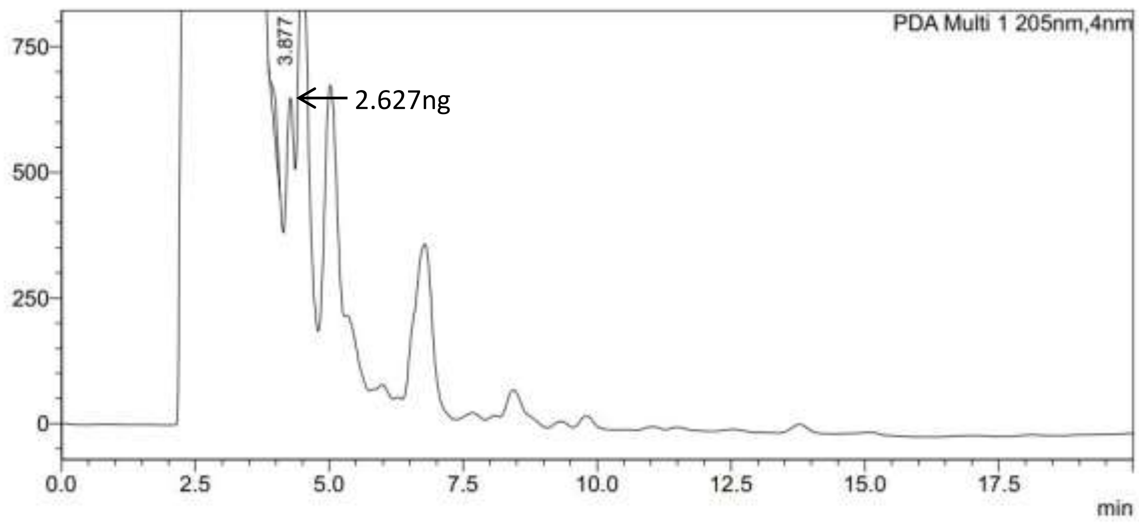

Figure 12: HPLC chromatogram of cold treated plants. Samples were harvested after 6 h of post treatment. The chromatogram represents chemo-profile of one sample only. The experiment was repeated three times.

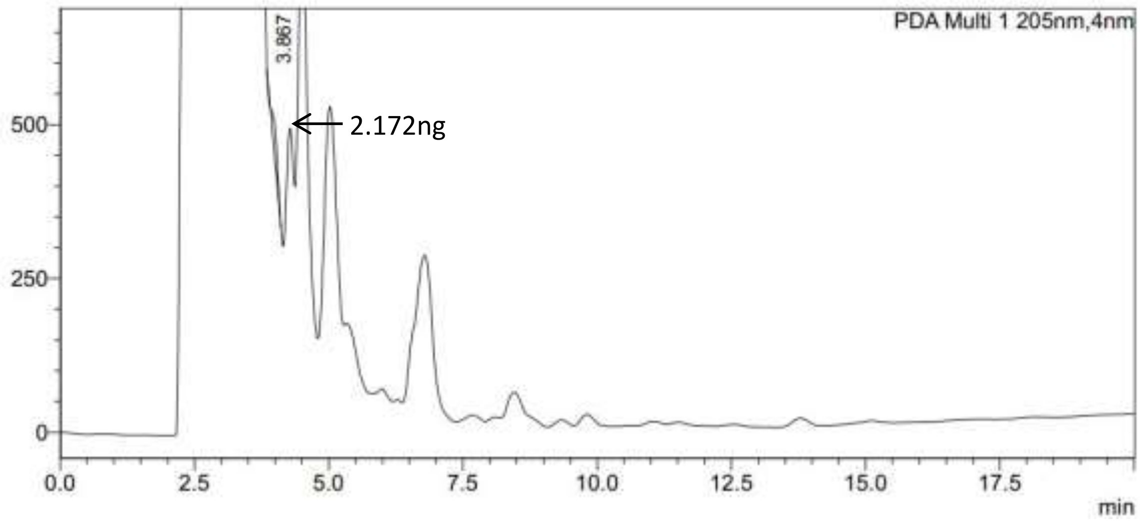

Figure 13: HPLC chromatogram of cold treated plants. Samples were harvested after 12 h of post treatment. The chromatogram represents chemo-profile of one sample only. The experiment was repeated three times.

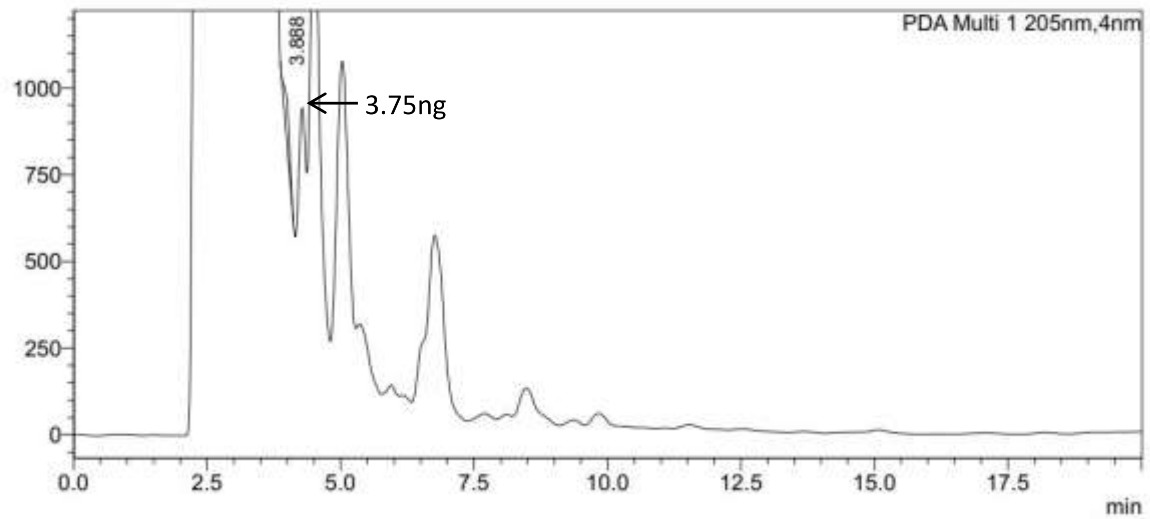

Figure 14: HPLC chromatogram of cold treated plants. Samples were harvested after 24 h of post treatment. The chromatogram represents chemo-profile of one sample only. The experiment was repeated three times.

-
