# Supplementary material for: Gene Silencing and Over-Expression Studies in Concurrence With Promoter Specific Elicitations Reveal the Central Role of WsCYP85A69 in Biosynthesis of Triterpenoids in Withania somnifera (L.) Dunal
Source: Front Plant Sci. 2019 Jul 5;10:842. doi: 10.3389/fpls.2019.00842 (PMC6624744; doi:10.3389/fpls.2019.00842)
Supplement: FILE S3 [file Data_Sheet_3.pdf]

a) Chromatograms of leaves infiltered with over-expression construct

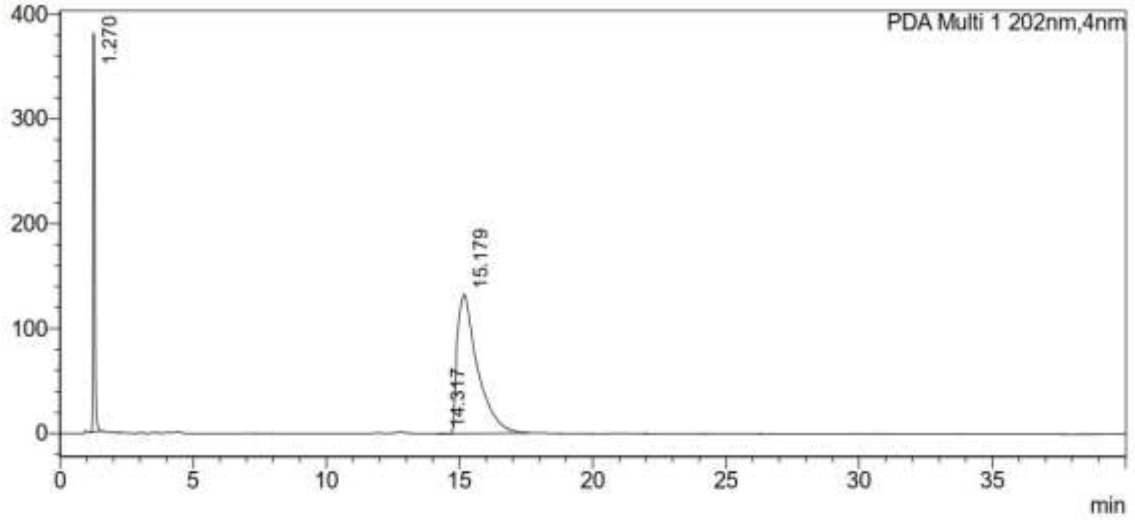

Figure 1: HPLC chromatogram of marker (stigmasterol)

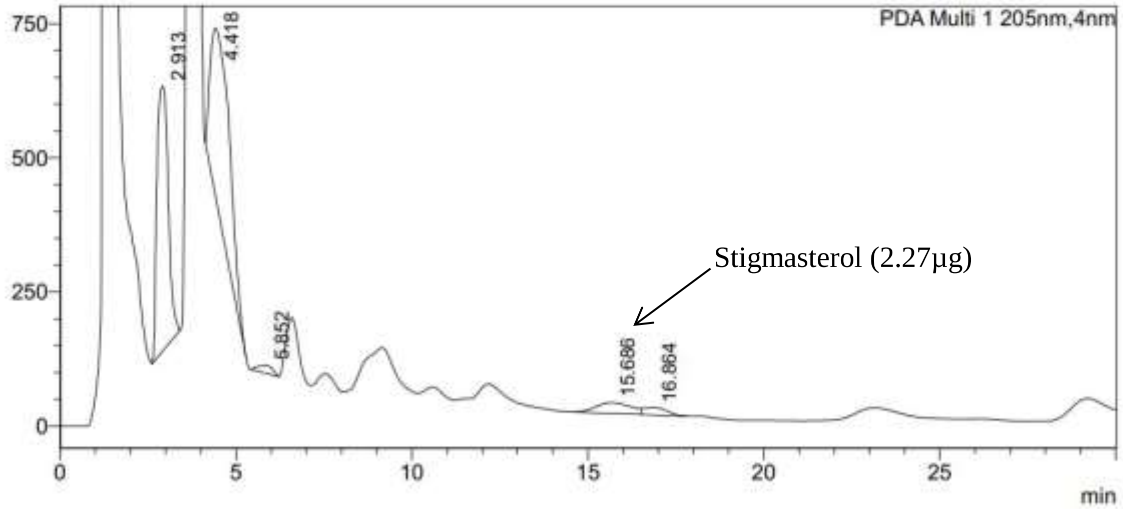

Figure 2: HPLC chromatogram of control leaves. The chromatogram represents chemo-profile of one sample only. The experiment was repeated three times.

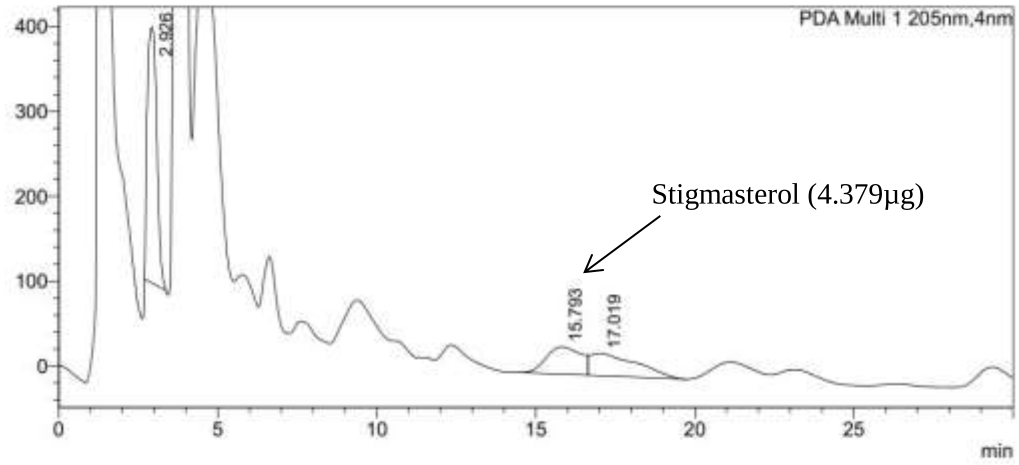

Figure 3: HPLC chromatogram of leaves infiltered with *WsCYP85A69*-pCAMBIA1302 construct, displayed higher accumulation of stigmasterol. The chromatogram represents chemo-profile of one sample only. Experiment was repeated three times.

b) Chromatograms of leaves infiltered with silencing constructs

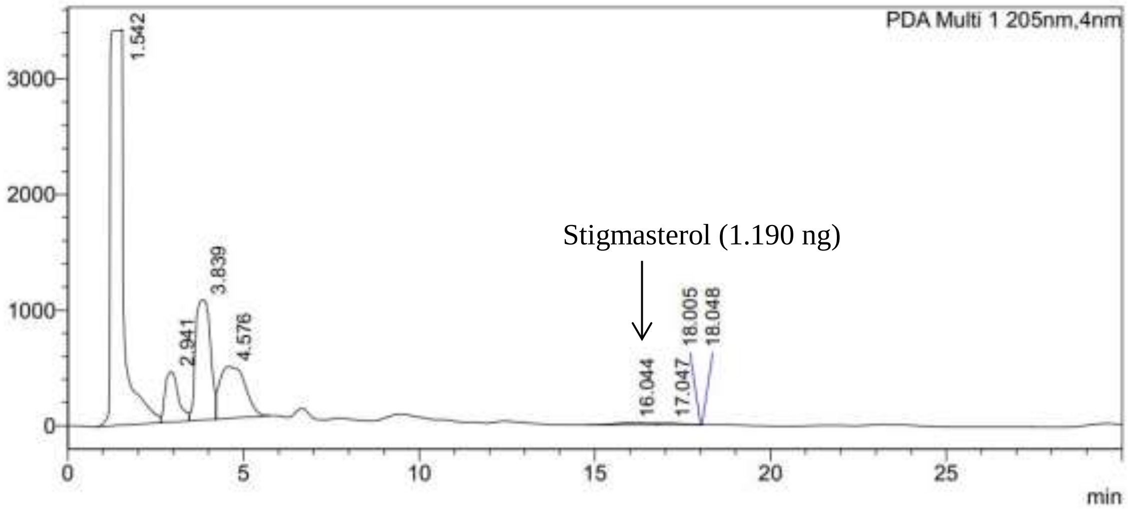

Figure 1: HPLC chromatogram of leaves infiltered with mi*WsCYP85A69-1*-pBI121 construct, displayed reduction in stigmasterol accumulation. The chromatogram represents chemo-profile of one sample only. Experiment was repeated three times.

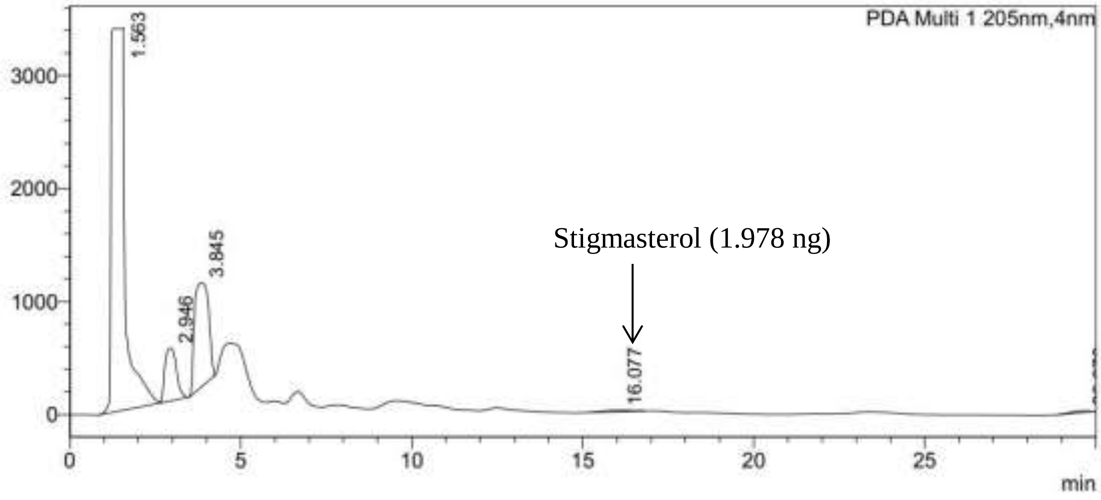

Figure 2: HPLC chromatogram of leaves infiltered with mi*WsCYP85A69-2*-pBI121 construct, displayed reduction in stigmasterol accumulation. The chromatogram represents chemo-profile of one sample only. Experiment was repeated three times.
