# Supplementary material for: Gene Silencing and Over-Expression Studies in Concurrence With Promoter Specific Elicitations Reveal the Central Role of WsCYP85A69 in Biosynthesis of Triterpenoids in Withania somnifera (L.) Dunal
Source: Front Plant Sci. 2019 Jul 5;10:842. doi: 10.3389/fpls.2019.00842 (PMC6624744; doi:10.3389/fpls.2019.00842)
Supplement: FILE S4 [file Data_Sheet_4.pdf]

a) HPLC chromatograms of leaves infiltered with over-expression construct

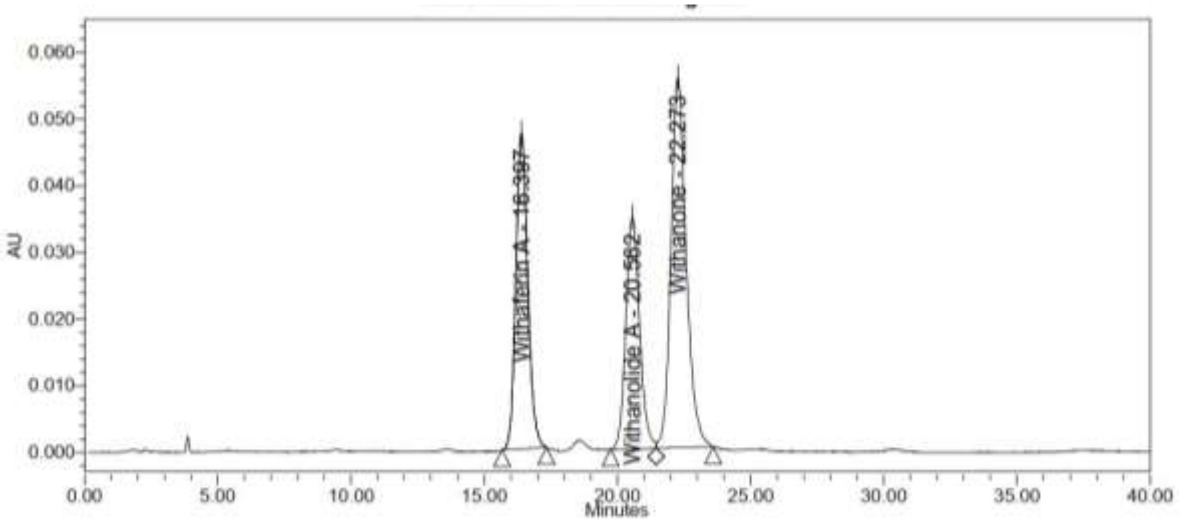

Figure 1: HPLC chromatogram of marker (WS-I, WS-II and WS-III).

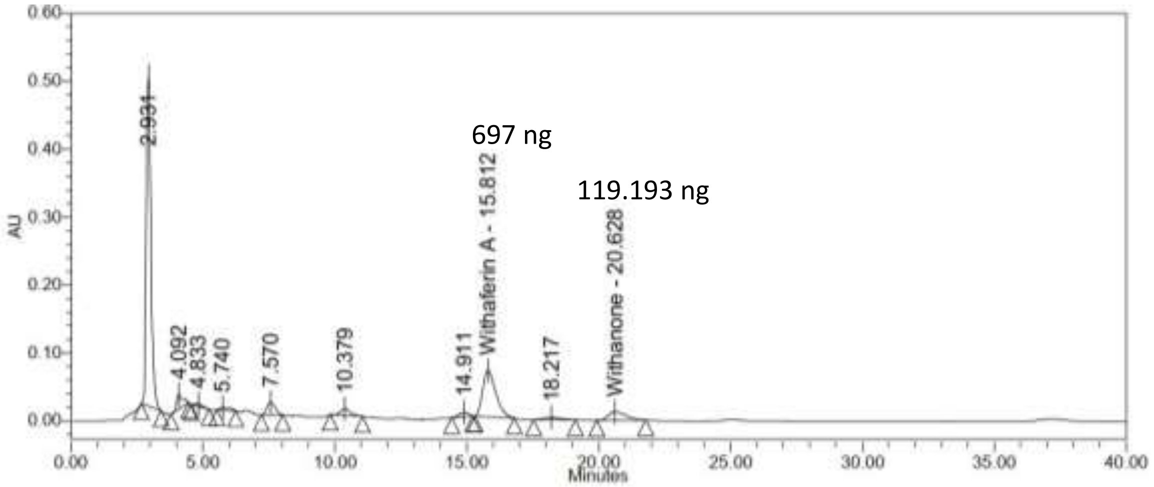

Figure 2: HPLC chromatogram of leaves of *W. somnifera.* The chromatogram represents chemo-profile of one sample only. Experiment was repeated three times.

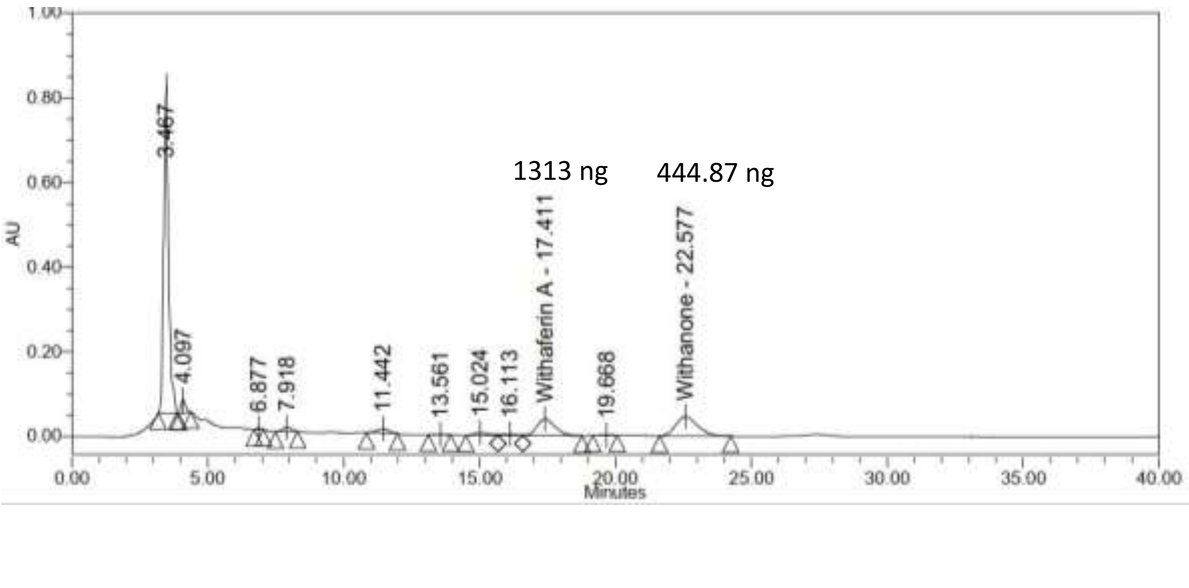

Figure 3: HPLC chromatogram of leaves infiltered with over-expression construct *WsCYP785A69-pCAMBIA1302*. The chromatogram represents chemo-profile of one sample only. Experiment was repeated three times.

b) Chromatograms of leaves infiltered with silencing constructs

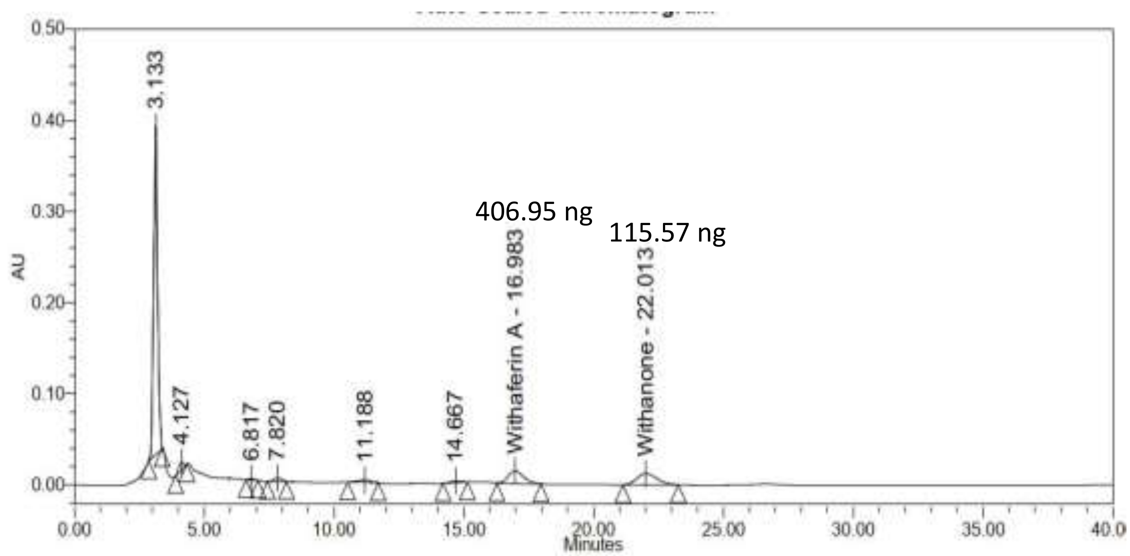

Figure 1: HPLC chromatogram of leaves infiltered with silencing construct (*WsCYP95A69-aMIR1*). The chromatogram represents chemo-profile of one sample only. Experiment was repeated three times.

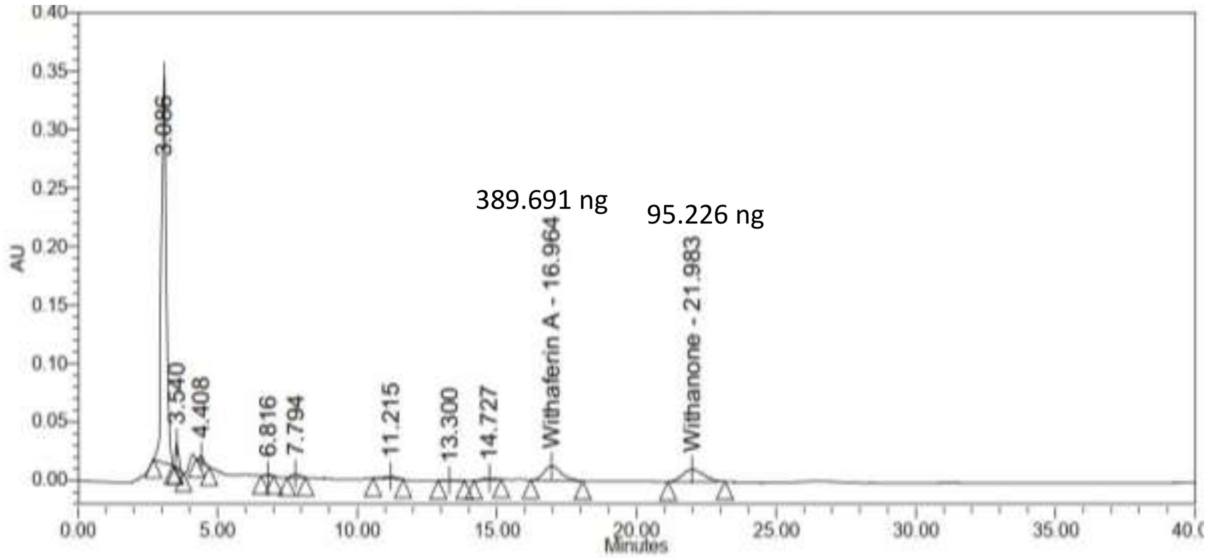

Figure 2: HPLC chromatogram of leaves infiltered with silencing construct (*WsCYP95A69-aMIR1*). The chromatogram represents chemo-profile of one sample only. Experiment was repeated three times.
