# Supplementary material for: Gene Silencing and Over-Expression Studies in Concurrence With Promoter Specific Elicitations Reveal the Central Role of WsCYP85A69 in Biosynthesis of Triterpenoids in Withania somnifera (L.) Dunal
Source: Front Plant Sci. 2019 Jul 5;10:842. doi: 10.3389/fpls.2019.00842 (PMC6624744; doi:10.3389/fpls.2019.00842)
Supplement: FILE S5 [file Data_Sheet_5.PDF]

>*Capsicum annuum*
ATGGCCTTTTTCTTAGTTTTTCTTGCTTCCTTTTTTGGGCTGTGCATCTTTAGTACTGCTTTATTAAGAT
GGAACCAAGTCAAGTACAACAAGAAGAATTTGCCCCCTGGTACTATGGGTTGGCCACTTTTTGGTGAGAC
TACTGAGTTTCTTAAGCTTGGCCCTTGCTTCATGAAAAACCAAAGAGCCAGATATGGGAGTTTTTTCAAG
TCACACATACTTGGGTGTCCAACAATAGTATCAATGGATCCAGAGTTGAATAGATATATATTAGTGAATG
AAGCAAAAGGACTGGTCCCAGGATACCCACAATCTATGCTAGACATTTTAGGTAAATGCAATATTGCAGC
TGTCAATGGTTCAGCTCACAAGTACATGAGGGGTGCATTGTTATCTTTAATTAGCCCTACCATGATCAGA
GACCAACTTTTGCCTAAAATTGATGAGTTCATGAGATCCCACTTAGCCAATTGGGATAACAAAGTCATTG
ATATTCAAGAAAAAACCAATAAGATGGCATTTCTGTCATCATTGAAGCAAATTGCTGGGATTGAATCTAG
CTCTTTGGCTCAAGAATTCATGCCTGAATTCTTCAAGCTAGTACTGGGAACTCTTTCACTACCCATCAAT
CTTCCCAAGACCAACTATCATCGCGGATTTCAGGCCAGGAAAATAATTGTGAGCCTACTAAGGACACTCA
TAGAAGAGAGGAGAGCTTCGAAGCAAATTCAACATGATATGCTTGGTTACCTAATGAATGAAGAAGCAAA
TCGATTCAAATTAACAGATGATGAGATGATTGATTTGATCATAACCATTTTGTACTCTGGATATGAAACT
GTTTCCACTACTTCTATGATGGCTGTCAAATATCTTCATGATCATCCTAAAGTTCTTGAAGAGCTCAGAA
AAGAACATATGGCAATTAGAGAAAAGAAAAAACCTGAAGATCCTATCGATTACAATGACTACAAGGCAAT
GCGGTTCACACGAGCTGTGATTTTAGAAACCTCCAGGTTAGCAACAATAGTAAATGGGGTCCTGAGAAAA
ACTACTCAAGATATGGAAATAAATGGGTACGTCATTCCTAAAGGATGGAGAATATACGTATACACGAGGG
AGTTGAATTACGATCCAAGGAGTTATCCTGATCCATATACATTCAATCCATGGAGATGGATGGATAAGAG
CCTGGAACACCAAAATTCGTTCTTAGTATTTGGAGGTGGTACTAGACAATGTCCTGGAAAAGAGCTTGGT
GTAGCAGAAATTTCTACATTTCTTCATTACTTCGTAACAAAGTACAGATGGGAAGAAGTAGGAGGAGATA
AACTGATGAAGTTTCCAAGAGTTGAGGCACCAAATGGTCTACGAATTAGAGTTTCAACTAATTAA
>*Solanum lycopersicum*
ATGGCCTTCTTCTTTGTTTTCCTTTCTTCCTTTTTTGGCCTATGCATCTTTTGTACTGCTTTATTAAGAT
GGAATCAAGTCAAGTATAACAACAAAAACTTGCCCCCTGGTACTATGGGTTGGCCACTTTTTGGTGAAAC
TACTGAGTTTCTTAAACTTGGTCCAAGTTTCATGAAAAACCAAAGAGCCAGATATGGGAGTTTTTTCAAA
TCACACATACTTGGTTGTCCAACAATTGTTTCAATGGATCCAGAACTGAATAGATATATACTTGTGAATG
AGGCAAAAGGACTGGTCCCAGGATACCCACAGTCTATGCTAGATATTTTAGGTAAATGTAATATTGCAGC
TGTCAATGGTTCAGCTCACAAGTACATGAGGGGTGCATTGTTATCTCTAATTAGCCCTACAATGATCAGA
GACCAACTTTTGCCCAAAATTGATGAGTTCATGAGATCCCACTTGAACAACTGGGATAATAAAGTTGTTG
ACATTCAAGAAAAAACCAATAAGATGGCATTTCTATCATCGTTGAAGCAAATTGCTGGGATTGAATCTAC
CTCTTTAGCTCAAGAATTCATGCCTGAATTCTTCAATCTAGTACTAGGAACTCTTTCACTACCAATCAAT
CTTCCCAACACCAACTATCATCGCGGATTTCAGGCAAGGAAAATTATTGTGAACTTATTAAGAACACTAA
TTGAAGAGAGAAGAGCTTCGAAGCAAATTCAACACGATATGCTTGGTTATCTGATGAATGAAGAAGCAAG
TCGATTCAAATTAACAGATGATGAGATGATTGATTTGATTATAACTATTTTGTACTCTGGCTATGAAACT
GTTTCCACTACTTCTATGATGGCTGTTAAATATCTTCATGATCATCCAAAAGTTCTTGAAGAACTCAGAA
AAGAACACATGGCTATTAGAGAAAAGAAAAAACCTGAGGATCCTATCGATTACAACGATTACAAGGCAAT
GCGGTTCACACGAGCTGTGATTTTAGAGACCTCCAGGCTAGCAACAATAGTAAATGGGGTTTTGAGAAAA
ACAACTCAAGATATGGAAATAAATGGGTATATCATTCCTAAAGGATGGAGAATATACGTATATACGAGGG
AGTTGAATTACGATCCAAGACTTTATCCTGATCCATATACATTCAATCCATGGAGATGGATGGATAAGAG
CCTGGAACACCAAAACTCATTTTTGGTATTTGGAGGTGGTACTAGACAATGTCCTGGAAAGGAACTTGGT
GTAGCAGAAATTTCTACATTTCTTCATTACTTCGTAACAAAATACAGATGGGAAGAAGTAGGAGGAGATA
AACTGATGAAGTTTCCAAGAGTTGAAGCACCAAATGGTCTACGGATTAGAGTTTCAACTCACTAA
>*Solanum tuberosum*
ATGGCCTTCTTCTTTGTTTTCCTTTCTTCCTTTTTTGGCCTATGCATCTTTTGTACTGCTTTATTAAGAT
GGAATCAAGTCAAGTATAACAACAAAAACTTGCCCCCTGGTACTATGGGTTGGCCACTTTTTGGTGAAAC
TACTGAGTTTCTTAAACTTGGTCCAAGTTTCATGAAAAACCAAAGAGCCAGATATGGGAGTTTTTTCAAA
TCACACATACTTGGTTGTCCAACAATTGTTTCAATGGATCCAGAACTGAATAGATATATACTTGTGAATG
AGGCAAAAGGACTGGTCCCAGGATACCCACAGTCTATGCTAGATATTTTAGGTAAATGTAATATTGCAGC
TGTCAATGGTTCAGCTCACAAGTACATGAGGGGTGCATTGTTATCTCTAATTAGCCCTACAATGATCAGA
GACCAACTTTTGCCCAAAATTGATGAGTTCATGAGATCCCACTTGAACAACTGGGATAATAAAGTTGTTG
ACATTCAAGAAAAAACCAATAAGATGGCATTTCTATCATCGTTGAAGCAAATTGCTGGGATTGAATCTAC
CTCTTTAGCTCAAGAATTCATGCCTGAATTCTTCAATCTAGTACTAGGAACTCTTTCACTACCAATCAAT
CTTCCCAACACCAACTATCATCGCGGATTTCAGGCAAGGAAAATTATTGTGAACTTATTAAGAACACTAA
TTGAAGAGAGAAGAGCTTCGAAGCAAATTCAACACGATATGCTTGGTTATCTGATGAATGAAGAAGCAAG
TCGATTCAAATTAACAGATGATGAGATGATTGATTTGATTATAACTATTTTGTACTCTGGCTATGAAACT
GTTTCCACTACTTCTATGATGGCTGTTAAATATCTTCATGATCATCCAAAAGTTCTTGAAGAACTCAGAA
AAGAACACATGGCTATTAGAGAAAAGAAAAAACCTGAGGATCCTATCGATTACAACGATTACAAGGCAAT
GCGGTTCACACGAGCTGTGATTTTAGAGACCTCCAGGCTAGCAACAATAGTAAATGGGGTTTTGAGAAAA
ACAACTCAAGATATGGAAATAAATGGGTATATCATTCCTAAAGGATGGAGAATATACGTATATACGAGGG
AGTTGAATTACGATCCAAGACTTTATCCTGATCCATATACATTCAATCCATGGAGATGGATGGATAAGAG
CCTGGAACACCAAAACTCATTTTTGGTATTTGGAGGTGGTACTAGACAATGTCCTGGAAAGGAACTTGGT

GTAGCAGAAATTTCTACATTTCTTCATTACTTCGTAACAAAATACAGATGGGAAGAAGTAGGAGGAGATA
AACTGATGAAGTTTCCAAGAGTTGAAGCACCAAATGGTCTACGGATTAGAGTTTCAACTCACTAA
>*Nicotiana tabacum*
ATGGCTTTCATCTTAGTTTTTCTTGCTTTCTTTTTTGGGCTGTGTATCTTCAGTACTGCTTTATTGAGAT
GGAATCAGGTCAAATATAACAAGAAAAGCCTTCCTCCTGGTACTATGGGTTGGCCACTTTTTGGTGAGAC
TACAGAATTTCTTAAGCAAGGTCCAAGCTTCATGAAAAATCAAAGAGCCAGATTTGGGAGTTTTTTCAAA
TCACACATACTTGGATGTCCAACAATAGTATCAATGGATTCAGAGTTAAATAGATACATATTGGTGAATG
AGGCAAAAGGACTGGTCCCAGGATATCCACAGTCTATGTTAGATATATTAGGAAAATGTAATATTGCAGC
TGTACATGGTTCTGCTCACAAGTACATGAGGGGTGCATTGTTATCTCTAATTAGCCCTACTATGATCAGA
GACCAACTTTTGCCTAAAATTGATGAGTTTATGAGATCCCACTTAACCAATTGGGATAGCAAAGTCATTG
ACATACAAGAAAAAACCAATAAGATGGCATTTTTGTCATCATTGAAGCAAATTGCTGGGATTGAATCTAC
CTCTTTAGCTCAAGAATTCATGCCTGAATTCTTCAAGCTAGTTTTAGGGACTCTTTCACTTCCTATCAAC
CTTCCCAACACCAACTACGGACGCGGATTGCAGGCAAGGAAAATTATTGTCAGCCTACTAAGGACACTAA
TAGAAGAGAGAAGAGCTTCAAAAGAAATCCAACATGATATGCTTGGTTACCTTATGAATGAAGAAGCAAA
TAGATATAAATTAACAGATGATGAAATGATTGATTTAATCATAACCATTTTGTATTCTGGATATGAAACT
GTTTCCACTACTTCAATGATGGCTGTTAAATATCTTCATGATCATCCTAAAGTTCTTGAAGAACTAAGAA
AAGAACACTTGGCTATTAGAGAAAAGAAAAAACCGGAGGATCCTATTGATTACAATGATTACAAGGCAAT
GCGGTTCACACGAGCAGTGATCCTTGAGACCTCCAGATTAGCAACAATAGTAAATGGAGTTCTAAGGAAA
ACCACTCAAGATATGGAAATAAATGGATACATTATTCCCAAAGGATGGAGAATATACGTATACACGAGGG
AGTTGAATTATGATCCAAGGCTTTATCCTGATCCTTATGCATTCAACCCATGGAGATGGCTGGATAAGAG
CCTGGAAAACCAAAATTCGTTCTTGGTATTTGGTGGAGGTACTAGACAATGTCCTGGGAAAGAACTTGGT
GTAGCAGAAATTTCTACATTTCTTCATTACTTTGTTACAAAATACAAATGGGAAGAAGTAGGAGGAGATA
AACTGATGAAATTTCCCAGAGTTGAAGCACCAAATGGTCTACGGATTAGAGTTTCAACTTACTAA
>*Nicotiana sylvestris*
CACAGCACACGCTTGGCCAGCTATTATGACTTAAAATTCTCAGAACTAAAGGAAGTCCATACCATTTAGA
CTTTGTCTGTGAGGTGGTTGGAACTTCATTGGTAGCTCTACATCAATGGCTTTCTTCTTAGTTTTTCTTG
CTTTCTTTTTTGGGCTGTGCATCTTCAGTACTGCTTTATTGAGATGGAATCAGGTCAAATATAACAAGAA
AAGCCTTCCTCCTGGTACTATGGGTTGGCCACTTTTTGGTGAGACTACAGAATTTCTTAAGCAAGGTCCA
AGCTTCATGAAAAATCAAAGAGCCAGATTTGGGAGTTTTTTCAAATCACACATACTTGGATGTCCAACAA
TAGTATCAATGGATTCAGAGTTAAATAGATACATATTGGTGAATGAGGCAAAAGGACTGGTCCCAGGATA
TCCACAGTCTATGTTAGATATATTAGGAAAATGTAATATTGCAGCTGTACATGGTTCTGCTCACAAGTAC
ATGAGGGGTGCATTGTTATCTCTAATTAGCCCTACTATGATCAGAGACCAACTTTTGCCTAAAATTGATG
AGTTTATGAGATCCCACTTAACCAATTGGGATAGCAAAGTCATTGACATACAAGAAAAAACCAATAAGAT
GGCATTTTTGTCATCATTGAAGCAAATTGCTGGGATTGAATCTACCTCTTTAGCTCAAGAATTCATGCCT
GAATTCTTCAAGCTAGTTTTAGGGACTCTTTCACTTCCTATCAACCTTCCCAACACCAACTACGGACGCG
GATTGCAGGCAAGGAAAATTATTGTCAGCCTACTAAGGACACTAATAGAAGAGAGAAGAGCTTCAAAAGA
AATCCAACATGATATGCTTGGTTACCTTATGAATGAAGAAGCAAATAGATATAAATTAACAGATGATGAA
ATGATTGATTTAATCATAACCATTTTGTATTCTGGATATGAAACTGTTTCCACTACTTCAATGATGGCTG
TTAAATATCTTCATGATCATCCTAAAGTTCTTGAAGAACTAAGAAAAGAACACTTGGCTATTAGAGAAAA
GAAAAAACCGGAGGATCCTATTGATTACAATGATTACAAGGCAATGCGGTTCACACGAGCTGTGATCCTT
GAGACCTCCAGATTAGCAACAATAGTAAATGGAGTTCTAAGGAAAACCACTCAAGATATGGAAATAAATG
GATACATTATTCCCAAAGGATGGAGAATATACGTATACACGAGGGAGTTGAATTATGATCCAAGGCTTTA
TCCTGATCCTTATGCATTCAACCCATGGAGATGGCTGGATAAGAGCCTGGAAAACCAAAATTCGTTCTTG
GTATTTGGTGGAGGTACTAGACAATGTCCTGGGAAAGAACTTGGTGTAGCAGAAATTTCTACATTTCTTC
ATTACTTTGTTACAAAATACAAATGGGAAGAAGTAGGAGGAGATAAACTGATGAAATTTCCCAGAGTTGA
AGCACCAAATGGTCTACGGATTAGAGTTTCAACTTACTAACTATTATTTCATGAATGTACAGAGGCAAAA
AAAAAAGGCCCCGAAAATATTTGTAGGCAGTAATCTGAGAGTAACAGGGATGTATATCTTATTGTTCCCG
CTAACACGATGATGAATTAAACACATTTTTTGCAGAGA
>*Lycopersicon esculentum*
ATGGCCTTCTTCTTAATTTTTCTTTCATCCTTTTTTGGCCTATGTATCTTTTGTACTGCTTTATTAAGAT
GGAATCAAGTCAAGTATAACCAAAAAAACTTGCCCCCTGGTACTATGGGTTGGCCACTTTTTGGTGAAAC
TACTGAGTTTCTTAAACTTGGTCCAAGTTTCATGAAAAACCAAAGAGCCAGATATGGGAGTTTTTTTAAA
TCACACATACTTGGTTGTCCAACAATTGTTTCAATGGATTCAGAACTGAACAGATATATACTAGTGAATG
AAGCGAAAGGACTGGTCCCAGGATACCCACAGTCTATGATAGATATTTTAGGAAAATGTAATATTGCAGC
TGTCAATGGTTCAGCTCACAAGTACATGAGGGGTGCATTGTTATCCCTAATTAGCCCTACAATGATCAGA
GACCAACTTTTGCCTAAAATTGATGAGTTTATGAGATCCCACTTAACCAATTGGGATAATAAAGTTATTG
ACATTCAAGAGAAAACCAATAAGATGGCATTTCTATCATCGTTGAAGCAAATTGCTGGTATTGAATCTAC
CTCTTTAGCTCAAGAATTCATGTCTGAATTTTTCAATCTAGTGCTAGGCACTCTTTCACTACCTATCAAT
CTTCCAAACACCAACTATCATCGCGGATTTCAGGCAAGGAAAATTATTGTGAACTTATTACGAACACTCA
TAGAAGAGAGAAGAGCTTCAAAGGAAATTCAACATGATATGCTTGGTTACCTGATGAATGAGGAAGCAAC
ACGATTCAAATTAACAGATGATGAGATGATTGATTTAATTATAACTATTTTGTACTCTGGATATGAAACT

GTTTCCACCACTTCTATGATGGCTGTGAAATATCTTCATGATCATCCAAAAGTTCTTGAAGAACTTAGAA
AAGAACACATGGCTATTAGAGAAAAGAAAAAACCTGAGGATCCTATCGATTACAACGATTACAGGTCAAT
GCGGTTCACACGAGCTGTGATTTTAGAGACCTCCAGGTTAGCAACAATAGTAAATGGGGTTTTGAGAAAA
ACAACTCAAGATATGGAAATAAATGGGTATATCATTCCTAAAGGATGGAGAATATACGTATATACAAGGG
AGTTGAATTACGATCCAAGACTTTATCCTGATCCATATTCGTTCAATCCATGGAGATGGATGGATAAGAG
CCTGGAACACCAAAACTCATTTTTGGTATTTGGAGGTGGTACTAGACAATGTCCTGGAAAGGAACTTGGT
GTAGCAGAAATTTCCACATTTCTTCATTACTTCGTAACAAAATACAGAAATAGGTGGAGATAAACTGATG
AAATTCCCAAGAGTTGAAGCACCAAATGGTCTACGGATTAGAGTTTCAGCTCACTAA
>*Sesamum indicum*
CTCTGCATCTTGAGTATCGCATTACTGAGATGGAATGAGGTAAGGTACAGGAAGAAAGGATTACCTCCTG
GTACCATGGGATGGCCAGTTTTTGGAGAGACCACTGAGTTTCTAAAGCAAGGTCCAGGCTTCATGAAAAA
CCAGAGATCAAGGTTTGGGAGTTTTTTCAAATCCCATATACTGGGCTGTCCTACCATAGTTTCGATGGAT
CCAGAGTTAAACAGATATATCCTGGTGAATGAGGCAAAAGGCCTTGTCCCTGGCTATCCACAGTCAATGT
TAGATATTTTAGGGAAATGCAACATTGCAGCTGTCCACGGTTCTGCCCACAAGCATATGAGAGGGGCATT
ACTTGCTCTTATTAGCCCTACCATGATTAAAGATCAGCTGTTGCCAAAAATCGACGATTTCATGAGATCC
CATCTGAGCGACTGGAACAACAAAATTGTTGATATTCAAGAGAAAACAAAAGAGATGGCGTTTCTCTCAT
CATTGAGACAAATAGCAAGCAAGGAAGCCAGCTCAATATCCCAATCTTTCATGCCTGAATTCTTTAAGCT
AGTACTGGGAACTCTTTCACTCCCTATCAACCTTCCCAACACAAACTATCATCGCGGGTTTCAGGCAAGA
CAACACATTACAAGCTTGTTGAGAAAACTGGTAGAAGAGAGAAGAGCTTCTGGAGAAAATCAAGAGGACA
TGCTTGGTTTTCTAGTAGGTGAAGAAAATAAATATAAGCTGAGTGATGAGGAGATGATAGACTTAATCAT
CACAATCTTGTATTCTGGGTATGAGACCGTATCTACTACTTCTATGATGGCTGTCAAGTATCTTCATGAC
CATCCAAAAGTACTTGAAGAATTAAGGAAAGAACATATGGCAATAAGAGATAGAAAAAAGCCTGAGGATC
CTATTCATTACAATGACTACAAATCAATGCGTTTCACACGTGCTGTCATATATGAGACATCAAGACTAGC
TACTATCGTGAACGGAGTTTTGAGGAAAACAACAAGAGATATGGAAATAAATGGATATCTGATTCCCAAG
GGATGGAGGATATATGTATACACAAGGGAGGTCAATTACGATCCATGCCTTTATCCTGATCCATTGACCT
TCAACCCATGGAGATGGCTGGACAAAGGCCTAGAAAATCAACACCATTTCTTGATATTCGGAGGAGGCAC
TAGACAGTGTCCTGGAAAGGAACTCGGACTGGCCGAAATTTCCACGTTCCTTCACTATTTTGTGACAAAG
TACAAATGGGAAGAAGTAGGGGGAGACAAGCTAATGAAATTCCCAAGGGTTGAAGCGCCTAACGGACTTC
GTATTAGGGTTTCGACTCACTAA
>*Ipomea nil*
TTCTTGGCCTCTGCATCCTCAACACAGCTTTGCTGAAATGGAACGATGTGAAGTACAGGATGATGAAAAG
CTTGCCTCCTGGAACCATGGGTTGGCCTGTTTTTGGAGAAACCACAGAGTTCTTGAAACAGGGCCCCAAC
TTCATGAAAAACCAGAGAGCAAGATATGGGTGTTTTTTCAAATCCCACATCCTGGGTTGTCCCACCATTG
TTTCAATGGATCCAGAGATGAACAGATATATTCTGGGAAACGAAGCCAAAGGGCTTGTCCCTGGCTACCC
ACAATCCATGCTTGACATTTTGGGGAAATGCAATATCGCAGCTGTTCATGGCTCTGCTCATAAGTACATG
AGAGGGGCACTCTTATCTCTCATCAGCCCTACCATGATCAGAGATCAGCTCTTGCCTAAAATTGATGAGT
TCATGAGATCCTACGTCAGCACCTGGGATGGACATGTCATTGACATCCAACAAAACACCAATAAGATGGC
TTTTCTGTCAGCTCTGAAGCAAATTGCTGGGATTGAATCCACCTCCATAGCTCAAGAATTCATGCCTGAG
TTCTTTAACCTTGTTCTGGGGACCCTTTCCCTCCCTATAAACCTTCCCAACACAAACTATCGCCGTGGGT
TTCAGGCAAGGAAGAACATTGTGTGCTTACTGAAAAAACTCATAGAAGAGAGGAGAGCTTCAGGGGAAAA
ACAACAAGACATGCTGGGATTCTTGATGAATGAAGATGAAAACCGATATAAACTAACGGATGATGAAATG
GTTGATCTCATCATAACAATCTTGTACTCTGGGTACGAGACTGTTTCCACCACTTGTATGATGGCTGTAA
AGTACCTCCATGATCATCCTAGAGCTCTAGAAGAGCTAAGGAAAGAGCATATGGAGATCAGAGCAAAGAA
GGGTCAGAATGATCCCATCAACTACAATGACTACAAATCCATGCGATTCACACGTGCTGTGATCTTTGAG
ACATCTCGGCTGGCAACAATAGTGAATGGGGTGCTGAGGAAGACAACTCGGGATATGGAACTCAATGGTT
ATATTATACCAAAAGGATGGAGAATATATGTGTATACAAGAGAGGTTAATTATGATCCAAGACTCTACCC
TGATCCATATTCTTTCAATCCATGGAGATGGCTGGATAAAGGCTTAGAGAACCAAAATAACTTTCTGGTT
TTTGGAGGAGGCACTAGGCAGTGTCCTGGAAAGGAACTTGGAATTGCAGAAATCTCAACATTCCTTCACT
ATTTTGTAACTAGATACAGATGGGAAGAAGTGGGGGGAGAGAAGCTGATAAAGTTTCCAAGAGTTGAAGC
ACCAAATGGGCTACACATCAGAGTTTCATCCTACTA
>*Olea europaea*
AGAATCACAGATTTCCCATCCAAAATTCAACTATTCACCCAAAAACAAATAGTTATGGCTGAAATTTTGA
TGAATTGGTAACTGAATCAAATTTCCAAAATACCCAACTAAAAGATAGGTTCTTTAGGTATTGAATGGCT
ATCTGATAAAGTTGTTGCCCCATAATAGTTAGTCACGAAGAATTCCTATTCAATTTTGTCCCTGCTTTTT
TTCTTTTTAATTTGAGGTTAAAGATTAGGTCCCTACCCTCTCGTGCAGAGTTTTGGCGTATCATGCCTCT
CTATACACGTACATATTGGATTTCTATAGTCTATATAAACTCAGACGCACACATGACCAGCATAAACTAC
AGAAAAAAGCCCTCTCTCTCTCTCTCTCACAAGTTGGTGGAGCTTGTAGGAAGAATCAATGGCTGTCCTA
ATTGTAATTCTTGGGGTATTTTTTGGGCTCTGTATTCTCAGTACCGCTTTACTCAGATGGAATGACGTCA
GGTACAGAAAGAAAGGATTGCCCCCTGGAACTATGGGGTGGCCAGTTTTTGGAGAGACAACTGAGTTTCT
AAAACAAGGTCCTAGCTTCATGAAGAACCAAAGATCAAGGTATGGGAGTTTTTTCAAATCACACTTATTG
GGTTGTCCTACCATCGTTTCAATGGATCCAGAGGTAAATAGATATATTCTAGTGAATGAAGCAAAAGGCC

TTGTCCCTGGCTATCCTCAGTCGATGCTAGACATCTTAGGGAAATGTAACATTGCAGCTGTCCATGGTTC
TGCTCACAAGCACATGAGAGGGGCATTGCTTGCACTCATCAGCCCCACCATGATCAAACAACAACTTCTG
CCAAAAATCGATGATTTTATGAGATCCCATTTGAGCAATTGGGATAACAAAAACATAGACATTCAAGAGA
AATCGAAAGAGATGGCTTTTCTGTCATCGCTGAGGCAAATAGCAAGCAATGAAGCCAGCTCAATATCTCA
AGCATTCATGCCTGAATTCTTCAAGCTAGTACTAGGAACTCTTTCACTGCCTATAAATCTTCCCAATACA
AACTATCATCGTGGATTCCAGGCAAGGAAAAATATTACAAGCTTGTTGAGAAAGCTTGTGGAAGAAAGAA
GAGCTTCAGGAGAAACTCAAAATGACATGCTTGGTTTCCTCATTAATGGAGGAGAGAAGAAATTTAATCT
AAGTGATGAGGAGATGATTGATTTGATCATCACAATTTTGTATTCTGGATATGAGACTGTATCAACTACT
TCAATGATGGCAGTTAAATATCTTCATGATCATCCAAAAGTACTCGAAGAATTAAGAAAAGAGCATGTGG
CAATTAGAGAAAAGAAAAGGCCAGAGGATCCTATAGATTATAATGACTACAAATCAATGTGCTTTACACG
TGCGGTTATCTATGAAACCTCAAGAATGGCCACAATAGTGAATGGAGTTTTGAGGAAAACGACTAATGAT
ATGGAAATAAATGGATTTGTTATTCCCGAAGGATGGAGGATATATGTTTACACGAGAGAGATTAACTATG
ATCCACACCTTTATCCTGATCCATTAACCTTCAATCCATGGAGATGGCTGGACAAAGGCCCAGAGTACCA
AAACTATTTCTTCATATTTGGAGGAGGCACTAGGCAGTGTCCTGGAAAGGAGCTTGGATTGGCAGAAATT
TCTACATTCCTTCATTACTTTGTGACAAGATACAATTGGAAAGAAATGGGGGGAGATGAGCTGATGAAAT
TTCCAAGGGTCGAAGCACCAAATGGACTTCAAATTAGGGTTTCAAGTAACTAATAATCATTGAATCAATG
TACAGAAAAATAACATAGCAGATTCAGATAGATATCAATCCTGTCCAGCTAATCCATTATAAAACTGAAA
ACCATTTTTGCTGTCTATGAAATCAGAACACAATAATTACGCTACAGAGAAACATTAATCAATCTGACTG
CGAAATGCAATACAACTGAAACTACGATAGTTAGAACACGAATCAATCGATCTGGAAAAATTTCGATTTT
AATATATTTATAGAGAATTTACCGTTTTGTTCATGAGGAACGGCAATTAATCAACTATAAAACGGTGACG
CAGATTTTATCGGTTTGTACCATACGGAATGATGCATCTGCAATTGAATTTTCTAATTGTTTTGCTCCAG
CTGCTGAACT
